# Exploratory Analysis of Commercial Olive-Based Dietary Supplements Using Untargeted and Targeted Metabolomics

**DOI:** 10.3390/metabo10120516

**Published:** 2020-12-19

**Authors:** Mar Garcia-Aloy, Nelli Groff, Domenico Masuero, Mauro Nisi, Antonio Franco, Furio Battelini, Urska Vrhovsek, Fulvio Mattivi

**Affiliations:** 1 Metabolomics Unit, Research and Innovation Centre Fondazione Edmund Mach, Food Quality and Nutrition Department, 38098 San Michele all’Adige, Italy; mar.garcia@fmach.it (M.G.-A.); nelligroff@gmail.com (N.G.); domenico.masuero@fmach.it (D.M.); urska.vrhovsek@fmach.it (U.V.); 2 Agraria Riva del Garda S.C.A., 38066 Riva del Garda, Italy; mauro@agririva.it (M.N.); furio@agririva.it (F.B.); 3 Ethifenol S.R.L., 24128 Bergamo, Italy; af@ethifenol.com; 4 Department of Cellular, Computational and Integrative Biology—CIBIO, University of Trento, 38123 Povo, Italy

**Keywords:** olive-based products, dietary supplements, foodomics, chemical composition, metabolomics, mass spectrometry

## Abstract

The market of olive-based dietary supplements (OBDS) is composed of a broad range of natural extracts claiming different health effects and often sold without a clear statement on their chemical composition. The aim of this survey was to characterize the chemical profiles of 14 commercially available OBDS. As many as 378 compounds were tentatively annotated in the analyzed samples. Although for most of metabolites the annotation at level I was prevented due to the lack of the analytical standard, the spectra obtained from high-resolution tandem mass spectrometry (MS/MS) measurements were very informative, allowing annotation of dozens of metabolites at level II or III. A targeted method allowed the quantification of 26 selected compounds. A large qualitative and quantitative variability was observed. The products obtained from buds by glyceric maceration were those with the lowest concentrations of all the quantified elements. The dose of 5 mg of hydroxytyrosol, corresponding to the European Food Safety Authority (EFSA) health claim, was only reached by four products, all of them originating from the olive fruit or the leaves. If we also take into consideration oleuropein, two additional products provide this daily amount. This work demonstrates the high complexity and diversity in the composition of OBDS.

## 1. Introduction

The current market for dietary supplements makes a wide range of products available to consumers. The market value of such products in the European Union (EU) reached 7.2 billion euros in 2015, with Italy being the leading country with 1.4 billion euros, projected to increase to 1.6 billion euros in 2020. Italy was followed by Germany (967 m€), Russia (888 m€), the UK (737 m€), and France (684 m€) [1]. Their consumption is probably enhanced by the fact that the sale of these products is usually accompanied by messages that highlight their composition (i.e., nutrition claims) and/or their beneficial effects on human health (i.e., health claims), and does not require an explicit prescription and/or supervision from a health professional. In Europe, food supplements are regulated according to national laws in harmony with EU Directive 2002/46/EC, whereas their labeling, in terms of nutrition and health claims, is governed by Regulation (EC) No 1924/2006. Only authorized claims are allowed, following the evaluation of the relevant scientific evidence by the European Food Safety Authority (EFSA). These claims represent an important opportunity in the marketing strategy of products containing doses of active ingredients of proven efficacy for the target population. One of these claims refers to the beneficial effects of olive oil, specifically stating that “olive oil polyphenols contribute to the protection of blood lipids from oxidative stress” [2]. This claim is allowed for olive oils that contain at least 5 mg of hydroxytyrosol and its derivatives (e.g., oleuropein complex and tyrosol) per 20 g of olive oil. However, although such a condition refers to olive oil as a food constituent, the EFSA later confirmed that the use of hydroxytyrosol extract as functional ingredient has not been associated with adverse effects and is safe for human health [3].

In line with this, there is a considerable variety of diverse commercial olive-based dietary supplements (OBDS) already available in the market. They differ in terms of administration (i.e., capsules, pills, liquid or solid extracts, etc.) and parts of the plant used (i.e., fruit, leaves, buds, etc.), claiming different health effects and often sold without a clear statement on their chemical composition. It is difficult for consumers to find their way around this jungle. The facility with which consumers can acquire these products makes a comprehensive knowledge of their composition necessary.

In the last few years, olive oil by-products such as leaves, pomace, olive mill wastewaters or stones have attracted increasing attention as an alternative source of olive phenolic compounds, which have an already recognized potential health value [4]. A motivation for the valorization of these waste products is to promote and implement the circular economy principle in the industry and move towards a sustainable agriculture. One of the exploitation field of these by-products could be the production of functional foods, food supplements or nutraceuticals. A functional food has been defined as “a food, which beneficially affects one or more target functions in the body, beyond adequate nutritional effects, in a way that is relevant to either an improved state of health and well-being and/or reduction of risk of disease.” [5]. On the other hand, food supplements have been defined as “concentrated sources of nutrients or other substances with a nutritional or physiological effect that are marketed in “dose” form (e.g., pills, tablets, capsules, liquids in measured doses) that are intended to correct nutritional deficiencies, maintain an adequate intake of certain nutrients, or to support specific physiological functions” [6]; whereas nutraceuticals are mostly referred to as pharma-foods and considered to be in the grey area after diet, but before drugs, with a specific health added value for the prevention or treatment of pathological conditions [7]. In parallel, the National Institutes of Health (NIH) of the United States of America (USA) define “dietary supplements” as those products intended to supplement the diet that are not medicines and that are not intended to treat, diagnose, mitigate, prevent, or cure diseases [8]. However, this area is not completely regulated in either Europe or the USA, and there is an open discussion on the need of improving the regulatory framework [7].

In the case of the OBDS currently present in the market, even if their origin is always the olive tree, their composition could differ accordingly to the part of the plant used. Therefore, the aim of this study was to identify and quantify the main compounds present in a range of different OBDS. Untargeted metabolomics was employed to profile the composition of OBDS in order to capture their chemical complexity. Targeted quantitative analysis was conducted for a series of identified components for which commercial standards were available. We analyzed 14 OBDS bought through the internet and in different local shops. This work provides valuable information to the community about the composition and diversity of these products.

## 2. Results

### 2.1. Untargeted Metabolomics: Identified Compounds

After processing the files, 487 features were detected in negative and 527 in positive mode, which were annotated to a total of 378 unique compounds. Out of the 378 constituents, 202 were annotated at different levels of confidence (Table 1): 15 were identified at level I, 66 at level II, and 121 at level III. Of these, 21 were unknown compounds assigned to a generic class based on their fragmentation patterns (all of them assigned to level III). The remaining 176 compounds present in the samples were not assigned (Table S1, level IV or V, depending on whether the molecular formula could be found or not) [9]. Most of the annotated compounds were polyphenols or secoiridoid-derived constituents, although some polar metabolites, such as organic acids, sugars, sugar alcohols and sugar acids, were also observed. Table S2 lists the main fragment ions generated by the tandem mass spectrometry (MS/MS) experiments in both ionization modes for all detected compounds. In the table we report the fragments with a relative intensity >10% together with the corresponding diagnostic ions (when available).

Organic acids constituted 6 out of 180 identified compounds (C001–C006). These included the quinic, malic, citric, isocitric and succinic acids. All of them were identified from the comparison of their retention times (RTs) and fragmentation patterns with their respective analytical standards. Additionally, gluconic acid (level II) was annotated based on the match of its MS^2^ pattern with the one reported in the mzCloud database.

Six sugars were also annotated (C007–C012). They were a 6-carbon sugar alcohol, one sugar acid, and one each of a mono-, di-, tri- and tetra-saccharide. With the exception of both sugar acids, all their RT and MS^2^ spectra were compared with the analytical standards of the corresponding different isomers. For each compound, both the RT and MS^2^ spectra of each isomer were mostly identical. For that reason, the specific isomer for each of the compounds could not be specified. Therefore, in order to establish their exact identity, it would be necessary to use another analytical technique capable of separating the different isomers.

As already mentioned, and as expected, the vast majority of compounds were in the polyphenols and secoiridoids categories. They will be discussed together due to their high interrelationship, often as constituents of larger compounds comprising subunits of both types of molecules. As expected, among them there was hydroxytyrosol (C050), which was also confirmed by comparison with the chemical standard. Additionally, 3,4-dihydroxyphenylglycol (the hydroxylated derivative of hydroxytyrosol, C049) and tyrosol glucoside (C065, also known as salidroside) were also identified by comparison with their chemical standards. Two hydroxytyrosol glucosides were also identified (C051-C052). Both compounds presented the same fragmentation profile as hydroxytyrosol. That is, the ions at m/z −153.0557, −135.0452 and −123.0452 in negative mode, and the fragments at m/z +155.0703 and +137.0597 in the positive mode. A similar MS^2^ profile showed hydroxytyrosol rutinose (C053), the spectrum of which in negative mode showed the part of the hydroxytyrosol characteristic ions corresponding to the loss of the rhamnose moiety at m/z −315.1085. In the positive mode, the diagnostic fragments were +301.1283 (loss of hexose), +265.1070 (loss of hexose and two water groups) and +309.1180 (loss of a dehydrated hydroxytyrosol moiety). Compounds C054–C057 could be dimers of hydroxytyrosol. All isomers presented diagnostic fragments in their MS^2^ spectra, but they were not always the same. Among them, there were the aforementioned characteristic ions of hydroxytyrosol, together with the fragments obtained by the loss of one or two CH_2_O moieties (i.e., the ions at m/z −275.0925 and −245.0819, respectively) or by the loss of a water unit at m/z −287.0925. The small differences in the fragmentation pattern are probably related to how the two hydroxytyrosol molecules were joined.

A total of 15 compounds (C017–C031) were tentatively identified as apigenin-, luteolin- and quercetin-derived flavonoids. MS^2^ experiments revealed that, in most cases, the ion corresponding to the aglycone was the main fragment. Flavonoid rutinosides also showed the ion fragment derived from the loss of the rhamnose moiety in the positive mode. The level II of identification was assigned when the pseudo-MS^3^ of the corresponding aglycone was available and it matched with the data from mzCloud, whereas others were assigned as level III since there was not enough information to confirm the fragmentation pattern of the aglycones. It is biologically plausible that they are these aglycones (and not any other potential isomer) since they have already been detected in olive matrices [10].

Another major class of polyphenols was that of cinnamic acid derivatives (C032–C048). The main fragment in negative ion mode for both caffeic acid hexoside (C032) and rutinoside (C033) was the ion corresponding to deprotonated caffeic acid (−179.0350), which differs from the precursor ion matched with the loss of a hexose and a rutinose moiety, respectively. On the other hand, the MS^2^ spectra of caffeic acid ethyl ester (C034), as well as those for both caffeoyl-threnoic acid isomers (C035–C036) were composed by the characteristic fragment ions of caffeic acid in negative mode (i.e., m/z at −179.0350, −161.0244, −135.0452). Two features eluting at 336 s from negative ion mode dataset clustered together, one of them corresponding to the dehydrated form of caffeic acid (−161.0244). Indeed, the MS^2^ spectrum in negative mode of the ion with the highest m/z value (−477.1402), which matched the C_23_H_26_O_11_ molecular formula, was composed by the ion derived from the loss of the caffeoyl moiety of the compound (−315.1085), as well as by the aforementioned characteristic ion of caffeic acid dehydrated (−161.0244). The MS^2^ spectrum in positive also included the latter ion (+163.03899), as well as the ion formed by the loss of a water together caffeic (+299.1126) and hydroxytyrosol (+325.09184) moieties, respectively, a part of the ions generated by the loss of one (+443.1334) and two (+461.1443) water moieties. Therefore, considering this information, this compound was tentatively annotated as calceolarioside B (C037), a caffeoyl phenylethanoid glycoside which consists of a molecule of caffeic acid linked to a molecule of hydroxytyrosol and another of glucose. On the other hand, the feature at m/z −359.0773 corresponded to the C_18_H_16_O_8_ molecular formula. This formula could match either rosmarinic acid (C038) (ester of caffeic acid and 3,4-dihydroxyphenyllactic acid) or caffeic-dihydrocaffeic acid. The MetFrag fragmentator only retrieved a potential match of all 4 observed ions in MS^2^ for rosmarinic acid (for caffeic-dihydrocaffeic acid –197.0456 did not match). Additionally, the observed MS^2^ spectrum also matched that reported for rosmarinic acid in the MassBank database (spectrum PR040215). The MS^2^ spectrum of coumaroylquinic acid (C039) agreed with that reported in the MassBank database (PR309014), while the spectra of (neo)chlorogenic acids (C041–C042) coincided with the information contained in mzCloud. The compounds C43 and C44 exhibited the same deprotonated ion at m/z 623.1977, relative to the formula C_29_H_36_O_15_. Both peaks displayed the fragments at m/z −461.1663 and −315.1083, corresponding to the neutral loss of a caffeic acid and a further loss of a rhamnose. The first eluting peak was identified as verbascoside using an authentic standard, and the second as isoverbascoside, known to elute afterward under reverse-phase (RP) chromatographic conditions [11]. The MS^2^ of the molecular ion of hydroxy-verbascoside (C045) (−639.1931) yielded to the main daughter ion at m/z −621.1825, corresponding to the water loss, and three minor fragments at m/z −529.1563, corresponding to the loss of a catechol unit, m/z −487.1457 corresponding to the loss of a dehydrated hydroxytyrosol unit, and m/z −459.1508 associated with the loss of a dehydrated caffeoyl moiety [12]. On the other hand, for both methyl-hydroxy-verbascoside isomers (C046–C047), the ion at m/z −621.1825 could be formed by the loss of a methyl unit, and that at m/z −459.1508 by the further loss of the caffeoyl group [10]. The same diagnostic ions are observed for the MS^2^ of dimethyl-hydroxy-verbascoside (C048).

Five features with the same accurate mass at m/z 539.1770 in negative mode but eluting at different RTs were also detected in the study samples (C068–C072). This value corresponds to the molecular formula C_25_H_32_O_13_, which matches oleuropein (a glycoside of hydroxytyrosol and elenolic acid), one of the main compounds in olives and their derived products. With the exception of the first peak, all of them showed the same fragment ions at m/z −377.1242, −345.0980, −307.0823, −275.0561 and −275.0925. The ion at m/z −377.1243 corresponds to the aglycone, while the other ones are formed by the consecutive losses of other parts of the molecule after the cleavage of the glucosidic bond, matching what is reported in the bibliography for oleuropein and oleuropein aglycone [13]. Oleuropein identity was confirmed by the comparison of its RT and fragmentation pattern with the corresponding analytical standard. Although the first peak presented the same ion at m/z −539.1770, any characteristic fragment for oleuropein was observed. In contrast, its MS^2^ spectrum included the m/z value corresponding to elenolic acid glucoside (−403.1246), as well as some of the characteristic fragment ions corresponding to the loss of the hexose moiety (−223.0612) and a further loss of a CO_2_ group (−179.07414) [10]. Since the oleuropein molecule is composed of an hydroxytyrosol, elenolic acid and glucose, we hypothesize that the observed difference in the MS^2^ spectrum could be related with a particular structure of this isomeric form, which led to the fragmentation of the part of the molecule corresponding to the elenolic acid moiety. Three isomers of oleuropein glucoside (C073–C075, i.e., diglycosides of hydroxytyrosol and elenolic acid) were also detected as some of the main compounds in the study samples. Again, the MS^2^ spectrum of the first isomeric form was the only one that did not show the characteristic fragmentation profile of oleuropein, but among their fragments there were the ions −539.1770 and −315.1085, corresponding to the oleuropein and hydroxytyrosol glucoside moieties, respectively. Finally, four isomers of oleuropein aglycone (C076–C079) were also annotated, all of them eluting after all oleuropein isomers. In this case, all MS^2^ spectra contained the characteristic fragments of oleuropein, and the main ion of the first two isomers gave a base peak at m/z 241.0718, which corresponds to the elenolic acid moiety [13]. Hydroxyoleuropein (C080) also showed the same fragmentation pattern as oleuropein (i.e., loss of hexose −393.1191, hexose and C_4_H_6_O moiety −323.0772, and additional loss of methyl group −291.0510), the loss of the water moiety (−537.1614) being the main fragment ion. The two late eluting isomeric forms of methyl-oleuropein-aglycones (C081–C083) showed similar MS^2^ profiles, characterized by the loss of the methyl and hydroxytyrosol moiety that led to the fragments at m/z −359.1136 and −255.0874, respectively, whereas the loss of both moieties occurred at m/z −223.0612. The ion at m/z −211.0976 corresponded to the loss of hydroxytyrosol and CO_2_ moiety. Finally, hydroxy-methyl-oleuropein (C084) also showed the fragment corresponding to the loss of hexose (−407.1348), in addition to that corresponding to the loss of the methyl group (−537.1614) and the one of dehydrated form of elenolic acid part (−233.0612). Another important group of annotated compounds with several isomeric and derived forms was that corresponding to decarboxy-methyl oleuropein aglycone (DOA). Eight isomeric forms and four derived compounds (some of them also with more than one isomer) of this constituent were observed (C086–C108). In general, their fragmentation patterns were characterized by the inclusion of some of the diagnostic ions of hydroxytyrosol (in negative: −153.0557, −123.0452 and −135.0452; in positive: +155.0703 and +137.0597) and/or decarboxymethyl elenolic acid dialdehyde (DEDA), in negative: −183.0663, −165.0557 and −139.0765; in positive: +185.0808 and +167.0703). Some of them also showed the ion led by the loss of hydroxytyrosol moiety, which were the ions at m/z −199.0612 of hydroxy-DOA, −201.0768 of hydrated-DOA −197.0819 for methyl-DOA and −229.1081 for acetal of DOA.

Two isomeric forms of ligstroside (C109–C110) and also ligstroside aglycone (C112) were also annotated. Ligstrosides are the tyrosol-derived compounds equivalent to oleuropein (instead of hydroxytyrosol moiety). All of them presented the characteristic fragments for ligstroside reported in the literature [13,14] (i.e., ions at m/z −361.1293, −291.0874 and −259.0976).

Two features presented the deprotonated pseudo-molecular ion at m/z 389.1089, which corresponded to the molecular formula C_16_H_22_O_11_ (C115–C116). Both compounds presented the fragment ions characteristic for oleoside and its isomeric form secologanoside at m/z −345.1191, −227.0561, −209.0455 and −183.0663. The first peak was annotated as oleoside since it has been reported that it elutes firstly in RP chromatographic conditions [10], whereas the second one was annotated as secologanoside due to the absence of the fragment −227.0561 and also since the fragment with the highest intensity was that at m/z −345.1191 [10]. Regarding caffeoyl- and coumaroyl-oleoside (C118, C120), the MS^2^ spectra of both of them included the ion led by the loss of the caffeoyl- and coumaroyl-moiety, respectively at m/z −389.1089, which corresponds to the deprotonated ion of oleoside. Additional loss of CO_2_ led to the fragments at m/z −345.1191. In both cases the base peak was that corresponding to the loss of CO_2_ moiety at m/z −507.1508 and −491.1559, respectively. For caffeoyl-oleoside glucose (C119), its base peak was that corresponding to the loss of the hexose moiety at m/z −551.1406, including also the aforementioned characteristic ions at −345.1191 and −507.1508.

The compounds C123–C127 showed a deprotonated ion at m/z −241.0718 and/or a protonated one at m/z +243.0863 compatible with the molecular formula C_11_H_14_O_6_. These compounds were identified as the different isomeric forms of elenolic acid based on the fragmentation mechanism in negative mode proposed by Kanakis and coworkers [13], which was characterized by the ion at m/z −209.0457 led by the loss of a CH_3_OH moiety, which further originated the fragments at m/z −165.0561, −139.0404 and −121.0300 after the consecutive losses of CO_2_, C_2_H_2_ and H_2_O. Two other characteristic ions were the ones at m/z −127.0405 and −95.0509. Both isomeric forms of elenolic acid glucoside (C128–C129) were annotated based on what is reported by Klen et al. [10]. The fragment at m/z −371.0984 corresponds to a neutral loss of the methyl group, while the fragment at m/z −223.0612 to the elimination of the hexose and a water moiety, giving rise to the m/z −179.0714 by the additional loss of CO_2_. Both hydroxy-elenolic acids (C132–C133) followed the same initial fragmentation mechanism of elenolic acid proposed by Kanakis et al. [13], i.e., the loss of the methyl group led the fragment at m/z −225.0406, which further lost a CO_2_ moiety giving the ion at −181.05083. An additional loss of another CO_2_ moiety originated the fragment at −137.0611, whereas the loss of just only one CO_2_ was the responsible for the ion at m/z −213.0767. The six isomeric forms of decarboxy-hydroxy-elenolic acid (C134–C139) presented the characteristic fragment ion proposed by Lozano-Sanchez and coworkers [15] at m/z −169.0870 generated by the neutral loss of a CO_2_ moiety, although in only two cases this was the highest fragment and in another two it was a very low-intensity ion. In these later cases, the highest fragment was the one at m/z −151.0763, corresponding to the additional loss of a water moiety. Another characteristic fragment of all of these isomers was that at m/z −139.0765, produced by the loss of a CO_2_ and CH_2_O moiety.

All four isomers of DEDA (C159–C162) presented the same fragmentation pattern. In negative mode, up to 2 losses of CO_2_ moiety (−139.0765 and −95.0866), and in positive up to 2 water losses (+167.0703 and +149.0597) and a further CO_2_ loss (+121.0648). The same scheme was observed for hydroxy-DEDA (C163). Also the three isomers of the hydrated product of DEDA (C164–C167) showed the same fragments: loss of CO_2_ (−157.0870), loss of H_2_O (−183.0663) and loss of CO_2_ and H_2_O (−139.0765). In addition, five peaks (C171–C175) were identified as isomers of DEDA alditol. In negative ion mode they showed the loss of the alditol moiety and the subsequent characteristic loss of CO_2_, giving the ions at m/z −183.0663 and −139.0765, respectively. The ion yield from the loss of the alditol moiety was also observed in positive ion mode (+185.0808), together with the fragments corresponding to the subsequent loss of two moieties of H_2_O and one of CO (ion at m/z +121.0648).

The MS^2^ spectrum of loganin (C176) in positive mode showed the ion yield from the loss of hexose in (+229.1071). This one, as well as that of all four annotated aglycones (C178–C181), was also characterized by the loss of methyl moiety (+197.0808) and further loss of CO (+169.0859) or COCH_2_ (+155.0703), loss of water (+211.0965) and further loss of methyl (+179.0703), CO (+151.0754) and another water (+133.0648).

Finally, the structural elements of some unknown compounds were tentatively deduced based on tandem mass spectra. For example, the compounds categorized as hydroxytyrosol derivatives (C182–C189) showed a neutral loss equivalent to an hydroxytyrosol moiety (136.0524) or the characteristic fragment of hydroxytyrosol (−135.0452, +137.0597). On the other hand, all constituents named as DEDA derivatives (C190–C197) showed a fragment corresponding to (de)protonated DEDA (−183.0663, +185.0808) and the ion derived from the loss of a CO_2_ (−139.0765) or water (−165.0557, +167.0703) moiety. Lastly, elenolic derivatives (C198–C202) were annotated as such since they presented at least one of the characteristic fragments of elenolic acid in negative ionization mode (i.e., m/z at −209.0457, −165.0561, −121.0300, −95.0509).

**Table 1:** Annotated compounds in study samples.

C	Compound	Formula	RT	Ions	LI [Ref]
C001	Gluconic acid	C_6_H_12_O_7_	54	195.051 [M-H]^−^	II (mzCloud)
C002	Quinic acid	C_7_H_12_O_6_	54	191.0562 [M-H]^−^; 192.0595 ^13^C[M-H]^−^; 279.0487 [add-1]^−^; 289.0331 [add-2]^−^; 371.12 [add-3]^−^; 373.1354 [add-4]^−^; 374.1389 ^13^C[add-4]^−^; 383.1196 [add-5]^−^; 384.123 ^13^C[add-5]^−^; 533.1725 [add-6]^−^	I (std)
C003	Malic acid	C_4_H_6_O_5_	56	133.0145 [M-H]^−^; 115.004 [M-H-H_2_O]^−^	I (std)
C004	Isocitric acid	C_6_H_8_O_7_	57	191.0199 [M-H]^−^; 192.023 ^13^C[M-H]^−^; 173.0095 [M-H-H_2_O]^−^; 129.0197 [M-H-H_2_O-CO_2_]^−^; 111.0091 [M-H-CH_4_O_4_]^−^; 87.0091 [M-H-C_3_H_4_O_4_]^−^; 85.0298 [M-H-C_2_H_2_O_5_]^−^; 210.0609 [M+NH_4_]^+^; 230.9903 [M+K]^+^	I (std)
C005	Citric acid	C_6_H_8_O_7_	71	191.0197 [M-H]^−^; 192.0229 ^13^C[M-H]^−^; 111.0091 [M-H-CH_4_O_4_]^−^; 87.0091 [M-H-C_3_H_4_O_4_]^−^; 210.0609 [M+NH_4_]^+^	I (std)
C006	Succinic acid	C_4_H_6_O_4_	77	117.0196 [M-H]^−^	I (std)
C007	Sugar alcohol	C_6_H_14_O_6_	54	181.0718 [M-H]^−^; 217.0486 [M+Cl]^−^; 219.0457 (2)^13^C[M+Cl]^−^; 227.0773 [M-H+HCOOH]^−^; 183.0864 [M+H]^+^; 184.0898 ^13^C[M+H]^+^; 200.1129 [M+NH_4_]^+^; 205.0683 [M+Na]^+^; 221.0423 [M+K]^+^; 222.0457 ^13^C[M+K]^+^; 281.0635 [M+H+CH_3_COOK]^+^; 165.0758 [M+H-H_2_O]^+^; 147.0652 [M+H-2(H_2_O)]^+^; 222.0609 [+]; 223.0404 [+]; 249.0373 [+]; 383.0954 [+]	I (std)
C008	Pentose acid	C_5_H_10_O_6_	53	165.0407 [M-H]^−^; 135.0302 [M-H-CH_2_O]^−^	III
C009	Hexose	C_6_H_12_O_6_	53	179.0563 [M-H]^−^; 180.0596 ^13^C[M-H]^−^; 215.033 [M+Cl]^−^; 225.0617 [M-H+HCOOH]^−^; 226.0651 ^13^C[M-H+HCOOH]^−^; 161.0458 [M-H-H_2_O]^−^; 143.0353 [M-H-2(H_2_O)]^−^; 113.0248 [M-H-2(H_2_O)-CH_2_O]^−^; 101.0247 [M-H-2(H_2_O)-COCH_2_]^−^; 198.0973 [M+NH_4_]^+^; 203.0527 [M+Na]^+^; 219.0267 [M+K]^+^; 145.0495 [M+H-2(H_2_O)]^+^; 127.0389 [M+H-3(H_2_O)]^+^; 85.0282 [M+H-2(H_2_O)-C_2_H_4_O_2_]^+^; 180.0867 [+]	I (std)
C010	Di-hexose	C_12_H_22_O_11_	54	341.109 [M-H]^−^; 377.0857 [M+Cl]^−^; 387.1145 [M-H+HCOOH]^−^; 360.1503 [M+NH_4_]^+^; 365.1057 [M+Na]^+^; 381.0796 [M+K]^+^; 325.1131 [M+H-H_2_O]^+^; 326.1166 ^13^C[M+H-H_2_O]^+^	I (std)
C011	Tri-hexose	C_18_H_32_O_16_	53	549.1673 [M-H+HCOOH]^−^; 522.2027 [M+NH_4_]^+^; 527.1577 [M+Na]^+^; 543.1321 [M+K]^+^	I (std)
C012	Tetra-hexose	C_24_H_42_O_21_	51	711.2202 [M-H+HCOOH]^−^	I (std)
C013	Glycerol	C_3_H_8_O_3_	62	93.0544 [M+H]^+^; 93.0545 [M+H]^+^	II (Metlin)
C014	Trihydroxy-octadecadienoic acid	C_18_H_32_O_5_	469	327.2176 [M-H]^−^; 346.259 [M+NH_4_]^+^	II (mzCloud)
C015	Trihydroxyoctadecenoic acid (I)	C_18_H_34_O_5_	465	329.2334 [M-H]^−^; 331.2481 [M+H]^+^	II (mzCloud)
C016	Trihydroxyoctadecenoic acid (II)	C_18_H_34_O_5_	492	329.2334 [M-H]^−^	II (mzCloud)
C017	Apigenin glucoside (I)	C_21_H_20_O_10_	329	431.0984 [M-H]^−^; 433.1128 [M+H]^+^	III
C018	Apigenin glucoside (II)	C_21_H_20_O_10_	363	431.0986 [M-H]^−^; 433.1128 [M+H]^+^; 434.1162 ^13^C[M+H]^+^	III
C019	Apigenin rutinoside (I)	C_27_H_30_O_14_	319	577.156 [M-H]^−^; 578.1594 ^13^C[M-H]^−^; 623.1616 [M-H+HCOOH]^−^; 579.1706 [M+H]^+^; 580.1741 ^13^C[M+H]^+^; 581.1764 (2)^13^C[M+H]^+^	III
C020	Apigenin rutinoside (II)	C_27_H_30_O_14_	350	623.1618 [M-H+HCOOH]^−^; 579.1706 [M+H]^+^; 580.1738 ^13^C[M+H]^+^	III
C021	Apigenin rhamnosyl acetyl-glucoside (I)	C_29_H_32_O_15_	336	621.1811 [M+H]^+^	III
C022	Apigenin rhamnosyl acetyl-glucoside (II)	C_29_H_32_O_15_	340	621.1811 [M+H]^+^	III
C023	Apigenin rhamnosyl acetyl-glucoside (III)	C_29_H_32_O_15_	367	619.1664 [M-H]^−^; 620.1698 ^13^C[M-H]^−^; 665.1722 [M-H+HCOOH]^−^; 666.1753 ^13^C[M-H+HCOOH]^−^; 621.1812 [M+H]^+^; 622.1844 ^13^C[M+H]^+^; 623.187 (2)^13^C[M+H]^+^	III
C024	Apiin	C_26_H_28_O_14_	299	563.1403 [M-H]^−^	III
C025	Methoxy-apigenin glucoside	C_22_H_22_O_11_	370	463.1234 [M+H]^+^	III
C026	Luteolin	C_15_H_10_O_6_	429	285.0402 [M-H]^−^	II (mzCloud)
C027	Luteolin glucoside (I)	C_21_H_20_O_11_	336	447.0933 [M-H]^−^; 448.0967 ^13^C[M-H]^−^; 493.0988 [M-H+HCOOH]^−^; 449.1078 [M+H]^+^; 450.111 ^13^C[M+H]^+^; 287.055 [M+H-hexose]^+^	II (mzCloud)
C028	Luteolin glucoside (II)	C_21_H_20_O_11_	362	447.0933 [M-H]^−^; 449.1077 [M+H]^+^; 450.111 ^13^C[M+H]^+^	III
C029	Luteolin rutinoside	C_27_H_30_O_15_	327	593.1505 [M-H]^−^; 595.1655 [M+H]^+^	III
C030	Quercetin glucoside	C_21_H_20_O_12_	333	463.088 [M-H]^−^; 465.1026 [M+H]^+^	II (mzCloud)
C031	Quercetin rutinoside	C_27_H_30_O_16_	324	609.1461 [M-H]^−^; 610.1493 ^13^C[M-H]^−^; 611.1606 [M+H]^+^	III
C032	Caffeic acid hexoside	C_15_H_18_O_9_	251	341.0878 [M-H]^−^	II (MassBank)
C033	Caffeic acid rutinoside	C_21_H_28_O_13_	236	487.1457 [M-H]^−^; 533.151 [M-H+HCOOH]^−^; 506.1867 [M+NH_4_]^+^	III
C034	Caffeic acid ethyl ester	C_11_H_12_O_4_	445	207.0662 [M-H]^−^	III
C035	Caffeoyl-threonic acid (I)	C_13_H_14_O_8_	238	297.0613 [M-H]^−^	II [16,17,18]
C036	Caffeoyl-threonic acid (II)	C_13_H_14_O_8_	276	297.0615 [M-H]^−^; 179.035 [caffeic-H]^−^; 135.0453 [caffeic-H-CO_2_]^−^	II [16,17,18]
C037	Calceolarioside B	C_23_H_26_O_11_	336	477.1402 [M-H]^−^; 161.0245 [caffeic-H-H_2_O]^−^	II [19]
C038	Rosmarinic acid	C_18_H_16_O_8_	371	359.0773 [M-H]^−^	II (MassBank)
C039	Coumaroylquinic acid	C_16_H_18_O_8_	292	337.0929 [M-H]^−^; 339.1077 [M+H]^+^	II (MassBank)
C040	Cinnamic acid hexoside	C_15_H_18_O_7_	350	309.0977 [M-H]^−^	III
C041	Neochlorogenic acid	C_16_H_18_O_9_	208	353.0876 [M-H]^−^; 355.1026 [M+H]^+^	II (mzCloud)
C042	Chlorogenic acid	C_16_H_18_O_9_	259	353.0878 [M-H]^−^; 354.091 ^13^C[M-H]^−^; 707.1827 [2M-H]^−^; 708.186 ^13^C[2M-H]^−^; 191.0561 [quinic-H]^−^; 355.1025 [M+H]^+^; 356.106 ^13^C[M+H]^+^; 372.1292 [M+NH_4_]^+^; 163.039 [caffeic+H-H_2_O]^+^	II (mzCloud)
C043	Verbascoside	C_29_H_36_O_15_	331	623.1978 [M-H]^−^; 624.2012 ^13^C[M-H]^−^; 625.2035 (2)^13^C[M-H]^−^; 659.1745 [M+Cl]^−^; 642.2388 [M+NH_4_]^+^; 643.2425 ^13^C[M+NH_4_]^+^; 479.1546 [M+H-rhamnose]^+^; 480.158 ^13^C[M+H-rhamnose]^+^; 471.1497 [M+H-hydroxytyrosol-H_2_O]^+^; 163.039 [caffeic+H-H_2_O]^+^; 325.0919 [caffeic acid glucoside+H-H_2_O]^+^; 326.0954 ^13^C[caffeic acid glucoside+H-H_2_O]^+^	I (std)
C044	Isoverbascoside	C_29_H_36_O_15_	345	623.1979 [M-H]^−^; 624.2013 ^13^C[M-H]^−^; 479.1548 [M+H-rhamnose]^+^; 325.092 [caffeic acid glucoside +H-H_2_O]^+^	II [10]
C045	Hydroxy-verbascoside	C_29_H_36_O_16_	297	639.1929 [M-H]^−^; 640.1961 ^13^C[M-H]^−^; 641.1985 (2)^13^C[M-H]^−^; 658.2339 [M+NH4]^+^; 325.092 [caffeic acid glucoside+H-H_2_O]^+^	II [12]
C046	Methyl-hydroxy-verbascoside (I)	C_30_H_38_O_16_	325	653.2084 [M-H]^−^	II [10]
C047	Methyl-hydroxy-verbascoside (II)	C_30_H_38_O_16_	354	653.2088 [M-H]^−^; 607.2031 [M-H-H_2_O-CO]^−^	III [10]
C048	Dimethyl-hydroxy-verbascoside	C_31_H_40_O_16_	346	667.2243 [M-H]^−^; 668.2276 ^13^C[M-H]^−^; 686.2658 [M+NH_4_]^+^	III [10]
C049	3,4-Dihydroxyphenylglycol	C_8_H_10_O_4_	74	169.0507 [M-H]^−^; 151.0402 [M-H-H_2_O]^−^	I (std)
C050	Hydroxytyrosol	C_8_H_10_O_3_	170	153.0558 [M-H]^−^; 154.0592 ^13^C[M-H]^−^; 307.1187 [2M-H]^−^; 308.1221 ^13^C[2M-H]^−^; 189.0325 [M+Cl]^−^; 199.0613 [M-H+HCOOH]^−^; 123.0455 [M-H-CH_2_O]^−^; 124.0489 ^13^C[M-H-CH_2_O]^−^	I (std)
C051	Hydroxytyrosol glucoside (I)	C_14_H_20_O_8_	80	361.114 [M-H+HCOOH]^−^; 317.1234 [M+H]^+^; 155.0703 [M+H-hexose]^+^; 137.0597 [M+H-hexose-H_2_O]^+^	II [20,21]
C052	Hydroxytyrosol glucoside (II)	C_14_H_20_O_8_	160	315.1084 [M-H]^−^; 316.1116 ^13^C[M-H]^−^; 316.1118 ^13^C[M-H]^−^; 631.2245 [2M-H]^−^; 631.2241 [2M-H]^−^; 351.0853 [M+Cl]^−^; 361.114 [M-H+HCOOH]^−^; 334.1498 [M+NH_4_]^+^; 335.1533 ^13^C[M+NH_4_]^+^	II [20,21]
C053	Hydroxytyrosol rutinoside	C_20_H_30_O_12_	216	461.1662 [M-H]^−^; 462.1697 ^13^C[M-H]^−^; 497.1432 [M+Cl]^−^; 507.1717 [M-H+HCOOH]^−^; 480.2075 [M+NH_4_]^+^	III
C054	Dimer of hydroxytyrosol (I)	C_16_H_18_O_6_	154	305.103 [M-H]^−^; 351.1084 [M-H+HCOOH]^−^; 324.1445 [M+NH_4_]^+^	III
C055	Dimer of hydroxytyrosol (II)	C_16_H_18_O_6_	187	305.1028 [M-H]^−^	III
C056	Dimer of hydroxytyrosol (III)	C_16_H_18_O_6_	248	305.103 [M-H]^−^; 324.1443 [M+NH_4_]^+^	III
C057	Dimer of hydroxytyrosol (IV)	C_16_H_18_O_6_	310	305.103 [M-H]^−^; 324.1443 [M+NH_4_]^+^	III
C058	Hydroxytyrosol-oxidised	C_8_H_8_O_3_	169	153.0546 [M+H]^+^; 123.044 [M+H-CH_2_O]^+^	III
C059	Lactone (ester with hydroxytyrosol) (I)	C_17_H_22_O_6_	383	321.1343 [M-H]^−^; 367.1397 [M-H+HCOOH]^−^; 340.1757 [M+NH_4_]^+^	II [13]
C060	Lactone (ester with hydroxytyrosol) (II)	C_17_H_22_O_6_	392	321.1342 [M-H]^−^; 323.149 [M+H]^+^; 340.1756 [M+NH_4_]^+^	II [13]
C061	Lactone (ester with hydroxytyrosol) (III)	C_17_H_22_O_6_	411	345.1335 [M+Na]^+^; 346.137 ^13^C[M+Na]^+^; 121.0647 [TYR+H-H_2_O]^+^; 165.0546 [coumaric+H]^+^	II [13]
C062	Lactone (ester with hydroxytyrosol) (IV)	C_17_H_22_O_6_	448	321.1344 [M-H]^−^; 185.082 [M-H-hydroxytyrosol]^−^; 323.149 [M+H]^+^; 324.1524 ^13^C[M+H]^+^; 340.1756 [M+NH_4_]^+^	II [13]
C063	Lactone glucoside (ester with hydroxytyrosol) (I)	C_23_H_32_O_11_	250	485.2016 [M+H]^+^	III
C064	Lactone glucoside (ester with hydroxytyrosol) (II)	C_23_H_32_O_11_	354	483.1871 [M-H]^−^; 502.2281 [M+NH_4_]^+^	III
C065	Tyrosol glucoside	C_14_H_20_O_7_	225	299.1138 [M-H]^−^; 335.0905 [M+Cl]^−^; 345.119 [M-H+HCOOH]^−^; 346.1224 ^13^C[M-H+HCOOH]^−^; 301.1285 [M+H]^+^; 618.2756 [2M+NH_4_]^+^; 318.1549 [M+NH_4_]^+^; 319.1584 ^13^C[M+NH_4_]^+^	I (std)
C066	Homogentisic acid	C_8_H_8_O_4_	139	167.0351 [M-H]^−^	II (mzCloud)
C067	Homovanillyl alcohol	C_9_H_12_O_3_	280	169.0859 [M+H]^+^; 170.0893 ^13^C[M+H]^+^; 186.1125 [M+NH_4_]^+^; 214.1438 [M+C_2_H_8_N]^+^	III
C068	Oleuropein isomer (I)	C_25_H_32_O_13_	372	539.177 [M-H]^−^; 540.1803 ^13^C[M-H]^−^; 558.2181 [M+NH_4_]^+^; 361.1285 [M+H-hexose-H_2_O]^+^; 137.0596 [OHTYR+H-H_2_O]^+^	III
C069	Oleuropein	C_25_H_32_O_13_	381	539.1769 [M-H]^−^; 540.18 ^13^C[M-H]^−^; 575.1533 [M+Cl]^−^; 721.2502 [4M+3H]^3+^; 722.2537 ^13^C[4M+3H]^3+^; 558.2182 [M+NH_4_]^+^; 559.2215 ^13^C[M+NH_4_]^+^; 586.25 [M+C_2_H_8_N]^+^; 379.139 [M+H-hexose]^+^; 380.1424 ^13^C[M+H-hexose]^+^; 361.1283 [M+H-hexose-H_2_O]^+^; 362.1319 ^13^C[M+H-hexose-H_2_O]^+^; 363.1343 (2)^13^C[M+H-hexose-H_2_O]^+^; 347.1131 [M+H-hexose-CH_3_OH]^+^; 329.1025 [M+H-hexose-H_2_O-CH_3_OH]^+^; 523.1811 [M+H-H_2_O]^+^; 137.0596 [OHTYR+H-H_2_O]^+^; 138.063 ^13^C[OHTYR+H-H_2_O]^+^; 165.0546 [coumaric+H]^+^	I (std)
C070	Oleuropein isomer (II)	C_25_H_32_O_13_	387	539.1771 [M-H]^−^	II [10,13]
C071	Oleuropein isomer (III)	C_25_H_32_O_13_	392	539.177 [M-H]^−^; 540.18 ^13^C[M-H]^−^; 575.1534 [M+Cl]^−^; 541.1915 [M+H]^+^; 542.1948 ^13^C[M+H]^+^; 543.1976 (2)^13^C[M+H]^+^; 558.2181 [M+NH_4_]^+^; 559.2212 ^13^C[M+NH_4_]^+^; 379.1388 [M+H-hexose]^+^; 380.1424 ^13^C[M+H-hexose]^+^; 361.1283 [M+H-hexose-H_2_O]^+^; 362.1318 ^13^C[M+H-hexose-H_2_O]^+^; 137.0596 [OHTYR+H-H_2_O]^+^; 165.0546 [coumaric+H]^+^	II [10,13]
C072	Oleuropein isomer (IV)	C_25_H_32_O_13_	404	539.1771 [M-H]^−^; 558.2181 [M+NH_4_]^+^; 379.1389 [M+H-hexose]^+^	II [10,13]
C073	Oleuropein glucoside (I)	C_31_H_42_O_18_	324	701.2294 [M-H]^−^	III
C074	Oleuropein glucoside (II)	C_31_H_42_O_18_	349	701.2295 [M-H]^−^; 702.2329 ^13^C[M-H]^−^; 747.2352 [M-H+HCOOH]^−^; 720.2706 [M+NH_4_]^+^; 721.2743 ^13^C[M+NH_4_]^+^; 541.1914 [M+H-hexose]^+^; 379.1389 [M+H-2(hexose)]^+^; 361.1258 [M+H-2(hexose)-H_2_O]^+^	II [10,13]
C075	Oleuropein glucoside (III)	C_31_H_42_O_18_	374	701.2294 [M-H]^−^	II [10,13]
C076	Oleuropein aglycone (I)	C_19_H_22_O_8_	436	377.1243 [M-H]^−^; 378.1276 ^13^C[M-H]^−^; 413.1011 [M+Cl]^−^; 396.1655 [M+NH_4_]^+^; 361.1283 [M+H-H_2_O]^+^; 362.1317 ^13^C[M+H-H_2_O]^+^	II [10,13]
C077	Oleuropein aglycone (II)	C_19_H_22_O_8_	451	377.1243 [M-H]^−^	II [10,13]
C078	Oleuropein aglycone (III)	C_19_H_22_O_8_	493	377.1243 [M-H]^−^; 378.1276 ^13^C[M-H]^−^; 755.256 [2M-H]^−^; 756.2594 ^13^C[2M-H]^−^; 413.1009 [M+Cl]^−^; 423.1299 [M-H+HCOOH]^−^; 307.0824 [M-H-C_4_H_6_O]^−^; 275.0926 [frag]^−^; 379.1388 [M+H]^+^; 380.1424 ^13^C[M+H]^+^; 396.1654 [M+NH_4_]^+^; 397.1689 ^13^C[M+NH_4_]^+^; 401.1207 [M+Na]^+^	II [10,13]
C079	Oleuropein aglycone (IV)	C_19_H_22_O_8_	504	377.1243 [M-H]^−^; 379.1388 [M+H]^+^	II [10,13]
C080	Hydroxy-oleuropein	C_25_H_32_O_14_	326	555.1718 [M-H]^−^; 556.175 ^13^C[M-H]^−^; 591.1483 [M+Cl]^−^; 574.213 [M+NH_4_]^+^; 377.1233 [M+H-hexose-H_2_O]^+^	III
C081	Methyl-oleuropein aglycone (I)	C_20_H_24_O_8_	329	151.0766 [frag]^−^	III
C082	Methyl-oleuropein aglycone (II)	C_20_H_24_O_8_	522	391.14 [M-H]^−^	III
C083	Methyl-oleuropein aglycone (III)	C_20_H_24_O_8_	546	391.14 [M-H]^−^	III
C084	Hydroxy-methyl-oleuropein	C_26_H_34_O_14_	376	569.1875 [M-H]^−^; 570.191 ^13^C[M-H]^−^; 571.193 (2)^13^C[M-H]^−^; 605.1642 [M+Cl]^−^; 588.2286 [M+NH_4_]^+^; 589.2322 ^13^C[M+NH_4_]^+^; 377.1233 [M+H-hexose-CH_3_OH]^+^	III
C085	Demethyloleuropein	C_24_H_30_O_13_	320	525.1613 [M-H]^−^	III
C086	Decarboxy-methyl oleuropein aglycone (DOA) (I)	C_17_H_20_O_6_	357	321.1334 [M+H]^+^	III
C087	DOA (II)	C_17_H_20_O_6_	362	321.1335 [M+H]^+^	III
C088	DOA (III)	C_17_H_20_O_6_	368	321.1335 [M+H]^+^	III
C089	DOA (IV)	C_17_H_20_O_6_	407	319.1187 [M-H]^−^; 320.122 ^13^C[M-H]^−^; 321.1336 [M+H]^+^; 338.1601 [M+NH_4_]^+^; 303.1229 [M+H-H_2_O]^+^	III
C090	DOA (V)	C_17_H_20_O_6_	422	319.1187 [M-H]^−^; 321.1334 [M+H]^+^; 322.1367 ^13^C[M+H]^+^; 338.1599 [M+NH_4_]^+^; 366.1911 [M+C_2_H_8_N]^+^	III
C091	DOA (VI)	C_17_H_20_O_6_	442	319.1186 [M-H]^−^; 320.1219 ^13^C[M-H]^−^; 639.2447 [2M-H]^−^; 640.2479 ^13^C[2M-H]^−^; 355.0954 [M+Cl]^−^; 365.1242 [M-H+HCOOH]^−^; 183.0663 [DEDA-H]^−^; 321.1334 [M+H]^+^; 322.1369 ^13^C[M+H]^+^; 663.2416 [2M+Na]^+^; 338.16 [M+NH_4_]^+^; 339.1634 ^13^C[M+NH_4_]^+^; 343.1153 [M+Na]^+^; 138.063 ^13^C[OHTYR+H-H_2_O]^+^	III
C092	DOA linked to hydroxytyrosol (I)	C_25_H_28_O_8_	453	457.1855 [M+H]^+^	III
C093	DOA linked to hydroxytyrosol (II)	C_25_H_28_O_8_	460	455.1713 [M-H]^−^; 457.1856 [M+H]^+^	III
C094	DOA linked to hydroxytyrosol (III)	C_25_H_28_O_8_	470	455.1712 [M-H]^−^; 303.123 [DOA+H-H2O]^+^	III
C095	Hydroxy-DOA	C_17_H_20_O_7_	387	335.1137 [M-H]^−^; 336.1169 ^13^C[M-H]^−^; 671.2348 [2M-H]^−^; 672.2381 ^13^C[2M-H]^−^; 673.2426 (2)^13^C[2M-H]^−^; 199.0613 [M-H-OHTYR]^−^; 337.1284 [M+H]^+^; 338.132 ^13^C[M+H]^+^; 354.1549 [M+NH_4_]^+^; 355.1584 ^13^C[M+NH_4_]^+^; 359.1104 [M+Na]^+^; 319.1181 [M+H-H_2_O]^+^	III
C096	Hydrated-DOA (I)	C_17_H_22_O_7_	347	337.1292 [M-H]^−^; 338.1324 ^13^C[M-H]^−^; 675.2658 [2M-H]^−^; 373.1059 [M+Cl]^−^; 201.0768 [M-H-OHTYR]^−^; 339.1439 [M+H]^+^; 340.1473 ^13^C[M+H]^+^; 356.1703 [M+NH_4_]^+^; 357.1738 ^13^C[M+NH_4_]^+^; 384.2016 [M+C_2_H_8_N]^+^	III
C097	Hydrated-DOA (II)	C_17_H_22_O_7_	362	337.1292 [M-H]^−^; 338.1325 ^13^C[M-H]^−^; 675.266 [2M-H]^−^; 201.0769 [M-H-OHTYR]^−^; 339.144 [M+H]^+^; 356.1706 [M+NH_4_]^+^	III
C098	Hydrated-DOA (III)	C_17_H_22_O_7_	386	337.129 [M-H]^−^; 319.1187 [M-H-H_2_O]^−^; 356.1705 [M+NH_4_]^+^	III
C099	Hydrated-DOA linked to hydroxytyrosol (I)	C_25_H_30_O_9_	423	473.1816 [M-H]^−^	III
C100	Hydrated-DOA linked to hydroxytyrosol (II)	C_25_H_30_O_9_	453	473.1816 [M-H]^−^; 474.1848 ^13^C[M-H]^−^; 492.2226 [M+NH_4_]^+^	III
C101	Hydrated-DOA linked to hydroxytyrosol (III)	C_25_H_30_O_9_	484	473.1817 [M-H]^−^; 519.1873 [M-H+HCOOH]^−^; 475.1962 [M+H]^+^; 492.2226 [M+NH_4_]^+^; 493.2259 ^13^C[M+NH_4_]^+^	III
C102	Hydrated-DOA linked to hydroxytyrosol glucoside	C_31_H_40_O_14_	386	635.2347 [M-H]^−^; 654.276 [M+NH_4_]^+^	III
C103	Methyl-DOA	C_18_H_22_O_6_	471	333.1345 [M-H]^−^; 335.1491 [M+H]^+^; 336.1526 ^13^C[M+H]^+^; 352.1756 [M+NH_4_]^+^	III
C104	Acetal of DOA (I)	C_19_H_26_O_7_	462	365.1607 [M-H]^−^	III
C105	Acetal of DOA (II)	C_19_H_26_O_7_	470	365.1607 [M-H]^−^; 366.1641 ^13^C[M-H]^−^; 731.3288 [2M-H]^−^; 401.1375 [M+Cl]^−^; 411.1663 [M-H+HCOOH]^−^; 229.1083 [M-H-OHTYR]^−^; 384.2019 [M+NH_4_]^+^; 389.1573 [M+Na]^+^	III
C106	Acetal of DOA (III)	C_19_H_26_O_7_	487	365.1609 [M-H]^−^	III
C107	Acetal of DOA (IV)	C_19_H_26_O_7_	506	365.1607 [M-H]^−^	III
C108	Acetal of DOA linked to hydroxytyrosol	C_27_H_34_O_9_	516	501.2129 [M-H]^−^; 502.2162 ^13^C[M-H]^−^; 520.2539 [M+NH_4_]^+^	III
C109	Ligstroside (I)	C_25_H_32_O_12_	411	523.182 [M-H]^−^; 524.1854 ^13^C[M-H]^−^; 525.1878 (2)^13^C[M-H]^−^; 559.1591 [M+Cl]^−^; 569.1875 [M-H+HCOOH]^−^; 361.1294 [M-H-hexose]^−^; 542.2232 [M+NH_4_]^+^; 543.2266 ^13^C[M+NH_4_]^+^; 363.1442 [M+H-hexose]^+^	II [13,14]
C110	Ligstroside (II)	C_25_H_32_O_12_	422	569.1873 [M-H+HCOOH]^−^; 542.2231 [M+NH_4_]^+^; 543.2265 ^13^C[M+NH_4_]^+^	II [13,14]
C111	Ligstroside glucoside	C_31_H_42_O_17_	344	685.2344 [M-H]^−^; 731.2403 [M-H+HCOOH]^−^; 732.2436 ^13^C[M-H+HCOOH]^−^; 704.2757 [M+NH_4_]^+^; 507.1861 [M+H-hexose-H_2_O]^+^	III
C112	Ligstroside aglycone	C_19_H_22_O_7_	535	363.144 [M+H]^+^; 380.1706 [M+NH_4_]^+^; 385.1259 [M+Na]^+^	II [14]
C113	Decarboxymethyl ligstroside aglycone	C_17_H_20_O_5_	382	349.1293 [M-H+HCOOH]^−^; 305.1386 [M+H]^+^; 306.142 ^13^C[M+H]^+^	III
C114	Hydroxy-decarboxymethyl-ligstroside aglycone	C_17_H_20_O_6_	431	319.1187 [M-H]^−^; 320.122 ^13^C[M-H]^−^; 321.1336 [M+H]^+^; 338.1601 [M+NH_4_]^+^	III
C115	Oleoside	C_16_H_22_O_11_	172	389.1088 [M-H]^−^; 390.1122 ^13^C[M-H]^−^; 779.2248 [2M-H]^−^; 435.1144 [M-H+HCOOH]^−^; 391.1237 [M+H]^+^; 392.1273 ^13^C[M+H]^+^; 408.1502 [M+NH_4_]^+^	II [10]
C116	Secologanoside	C_16_H_22_O_11_	253	389.1089 [M-H]^−^; 390.1121 ^13^C[M-H]^−^; 779.2249 [2M-H]^−^; 780.2281 ^13^C[2M-H]^−^; 391.1237 [M+H]^+^; 798.266 [2M+NH_4_]^+^; 408.1501 [M+NH_4_]^+^; 229.0707 [M+H-hexose]^+^; 211.0602 [M+H-hexose-H_2_O]^+^	II [10]
C117	Oleoside aglycone	C_10_H_12_O_6_	192	227.0562 [M-H]^−^; 229.0708 [M+H]^+^	III
C118	Caffeoyl-oleoside	C_25_H_28_O_14_	354	551.1405 [M-H]^−^; 552.1436 ^13^C[M-H]^−^; 553.1551 [M+H]^+^; 554.1585 ^13^C[M+H]^+^	III
C119	Caffeoyl-oleoside glucoside	C_31_H_38_O_19_	328	713.1932 [M-H]^−^	III
C120	Coumaroyl-oleoside	C_25_H_28_O_13_	379	535.1459 [M-H]^−^; 536.149 ^13^C[M-H]^−^; 537.1602 [M+H]^+^; 538.1635 ^13^C[M+H]^+^	III
C121	Dimethyl-hydroxy-octenoyloxy-secologanoside (I)	C_26_H_38_O_13_	400	559.2379 [M+H]^+^	II [13]
C122	Dimethyl-hydroxy-octenoyloxy-secologanoside (II)	C_26_H_38_O_13_	421	557.2239 [M-H]^−^; 558.2272 ^13^C[M-H]^−^; 559.2383 [M+H]^+^; 560.2418 ^13^C[M+H]^+^	II [13]
C123	Elenolic acid (I)	C_11_H_14_O_6_	264	243.0865 [M+H]^+^	III
C124	Elenolic acid (II)	C_11_H_14_O_6_	270	241.0718 [M-H]^−^; 243.0864 [M+H]^+^; 225.0759 [M+H-H_2_O]^+^	II [13]
C125	Elenolic acid (III)	C_11_H_14_O_6_	297	241.0718 [M-H]^−^; 243.0864 [M+H]^+^; 288.1444 [M+C_2_H_8_N]^+^	II [13]
C126	Elenolic acid (IV)	C_11_H_14_O_6_	318	241.0718 [M-H]^−^	II [13]
C127	Elenolic acid (V)	C_11_H_14_O_6_	371	241.0718 [M-H]^−^; 242.0751 ^13^C[M-H]^−^; 483.151 [2M-H]^−^; 484.1541 ^13^C[2M-H]^−^; 195.0663 [M-H-H_2_O-CO]^−^; 243.0864 [M+H]^+^; 244.0898 ^13^C[M+H]^+^; 260.1131 [M+NH_4_]^+^; 225.0758 [M+H-H_2_O]^+^; 211.0602 [M+H-CH_3_OH]^+^	II [13]
C128	Elenolic acid glucoside (I)	C_17_H_24_O_11_	280	403.1247 [M-H]^−^; 404.128 ^13^C[M-H]^−^; 449.1301 [M-H+HCOOH]^−^; 422.1658 [M+NH_4_]^+^; 423.1693 ^13^C[M+NH_4_]^+^; 243.0865 [M+H-hexose]^+^; 225.0758 [M+H-hexose-H_2_O]^+^; 151.039 [M+H-hexose-H_2_O-C_2_H_4_O_2_-CH_2_]^+^; 165.0546 [M+H-hexose-H_2_O-C_2_H_4_O_2_]^+^	II [10]
C129	Elenolic acid glucoside (II)	C_17_H_24_O_11_	300	403.1246 [M-H]^−^; 404.1278 ^13^C[M-H]^−^; 449.1299 [M-H+HCOOH]^−^; 405.1393 [M+H]^+^; 422.1658 [M+NH_4_]^+^; 225.0759 [M+H-hexose-H_2_O]^+^	II [10]
C130	Elenolic acid diglucoside	C_23_H_34_O_16_	257	611.1827 [M-H+HCOOH]^−^; 584.2185 [M+NH_4_]^+^	III
C131	Desoxy-elenolic acid	C_11_H_14_O_5_	307	225.077 [M-H]^−^; 451.1612 [2M-H]^−^; 271.0824 [M-H+HCOOH]^−^	III
C132	Hydroxy-elenolic acid (I)	C_11_H_14_O_7_	347	257.0666 [M-H]^−^; 259.0814 [M+H]^+^	III
C133	Hydroxy-elenolic acid (II)	C_11_H_14_O_7_	361	257.0666 [M-H]^−^	III
C134	Decarboxy-hydroxy-elenolic acid (I)	C_10_H_14_O_5_	145	213.0768 [M-H]^−^	II [15]
C135	Decarboxy-hydroxy-elenolic acid (II)	C_10_H_14_O_5_	197	213.0768 [M-H]^−^	II [15]
C136	Decarboxy-hydroxy-elenolic acid (III)	C_10_H_14_O_5_	228	213.0768 [M-H]^−^	II [15]
C137	Decarboxy-hydroxy-elenolic acid (IV)	C_10_H_14_O_5_	234	213.0768 [M-H]^−^	II [15]
C138	Decarboxy-hydroxy-elenolic acid (V)	C_10_H_14_O_5_	239	213.0769 [M-H]^−^; 427.1611 [2M-H]^−^; 151.0766 [M-H-H_2_O-CO_2_]^−^; 215.0915 [M+H]^+^; 232.1181 [M+NH_4_]^+^; 155.0703 [M+H-C_2_H_4_O_2_]^+^	II [15]
C139	Decarboxy-hydroxy-elenolic acid (VI)	C_10_H_14_O_5_	253	213.0769 [M-H]^−^; 215.0916 [M+H]^+^	II [15]
C140	Decarboxy-hydroxy-elenolic acid linked to hydroxytyrosol (I)	C_18_H_22_O_7_	230	349.1292 [M-H]^−^; 368.1706 [M+NH_4_]^+^	III
C141	Decarboxy-hydroxy-elenolic acid linked to hydroxytyrosol (II)	C_18_H_22_O_7_	330	368.1705 [M+NH_4_]^+^	III
C142	Decarboxy-hydroxy-elenolic acid linked to hydroxytyrosol (III)	C_18_H_22_O_7_	370	349.1293 [M-H]^−^; 395.1347 [M-H+HCOOH]^−^; 213.0769 [decarboxy-hydroxy-elenolic-H]^−^; 351.1439 [M+H]^+^; 368.1704 [M+NH_4_]^+^	III
C143	Decarboxy-hydroxy-elenolic acid linked to hydroxytyrosol (IV)	C_18_H_22_O_7_	390	349.1293 [M-H]^−^; 350.1325 ^13^C[M-H]^−^; 699.2659 [2M-H]^−^; 385.106 [M+Cl]^−^; 213.0769 [decarboxy-hydroxy-elenolic-H]^−^; 151.0766 [frag]^−^; 351.1439 [M+H]^+^; 352.1474 ^13^C[M+H]^+^; 373.1258 [M+Na]^+^; 333.1336 [M+H-H_2_O]^+^	III
C144	Decarboxy-hydroxy-elenolic acid linked to hydroxytyrosol (V)	C_18_H_22_O_7_	398	349.1293 [M-H]^−^; 351.1439 [M+H]^+^	III
C145	Methyl-elenolic acid (I)	C_12_H_16_O_6_	428	257.1021 [M+H]^+^	III
C146	Methyl-elenolic acid (II)	C_12_H_16_O_6_	459	257.1021 [M+H]^+^; 225.0758 [M+H-CH_3_OH]^+^	III
C147	Aldehydic form of decarboxymethyl elenolic acid (I)	C_10_H_16_O_5_	243	217.1071 [M+H]^+^; 234.1337 [M+NH_4_]^+^; 262.1651 [M+C_2_H_8_N]^+^; 199.0966 [M+H-H_2_O]^+^	III
C148	Aldehydic form of decarboxymethyl elenolic acid (II)	C_10_H_16_O_5_	255	217.1071 [M+H]^+^; 218.1105 ^13^C[M+H]^+^; 234.1337 [M+NH_4_]^+^; 262.1651 [M+C_2_H_8_N]^+^; 187.0965 [M+H-CH_2_O]^+^	III
C149	Aldehydic form of decarboxymethyl elenolic acid (III)	C_10_H_16_O_5_	289	215.0925 [M-H]^−^	III
C150	Aldehydic form of decarboxymethyl elenolic acid glucoside	C_16_H_26_O_10_	286	377.1453 [M-H]^−^; 396.1865 [M+NH_4_]^+^; 199.0965 [M+H-hexose-H_2_O]^+^	III
C151	Elenolic acid dialdehyde epimer linked to hydroxytyrosol glucoside	C_25_H_34_O_13_	371	541.1926 [M-H]^−^; 560.2338 [M+NH_4_]^+^; 363.1441 [M+H-hexose-H_2_O]^+^	III
C152	Elenolic acid dialdehyde epimer linked to hydroxytyrosol (I)	C_19_H_24_O_8_	415	379.1399 [M-H]^−^	III
C153	Elenolic acid dialdehyde epimer linked to hydroxytyrosol (II)	C_19_H_24_O_8_	448	379.1399 [M-H]^−^; 381.1544 [M+H]^+^	III
C154	Hydrated product of methyl-decarboxy-hydroxy-elenolic acid (I)	C_11_H_18_O_6_	240	245.1032 [M-H]^−^	III
C155	Hydrated product of methyl-decarboxy-hydroxy-elenolic acid (II)	C_11_H_18_O_6_	316	245.103 [M-H]^−^	III
C156	Hydrated product of methyl-decarboxy-hydroxy-elenolic acid (III)	C_11_H_18_O_6_	340	245.1031 [M-H]^−^	III
C157	Cannizzaro-like product of elenolic acid dialdehyde (I)	C_11_H_16_O_7_	54	278.1238 [M+NH_4_]^+^	III
C158	Cannizzaro-like product of elenolic acid dialdehyde (II)	C_11_H_16_O_7_	199	259.0822 [M-H]^−^; 215.0561 [M-H-C_2_H_4_O]^−^	III
C159	DEDA (I)	C_9_H_12_O_4_	154	202.1074 [M+NH_4_]^+^	II [15,22]
C160	DEDA (II)	C_9_H_12_O_4_	221	202.1074 [M+NH_4_]^+^	II [15,22]
C161	DEDA (III)	C_9_H_12_O_4_	260	183.0663 [M-H]^−^; 367.1399 [2M-H]^−^; 185.0808 [M+H]^+^; 186.0842 ^13^C[M+H]^+^; 202.1075 [M+NH_4_]^+^; 230.1388 [M+C_2_H_8_N]^+^	II [15,22]
C162	DEDA (IV)	C_9_H_12_O_4_	292	183.0663 [M-H]^−^; 184.0695 ^13^C[M-H]^−^; 367.1399 [2M-H]^−^; 368.1431 ^13^C[2M-H]^−^; 229.0718 [M-H+HCOOH]^−^; 185.0808 [M+H]^+^; 186.0842 ^13^C[M+H]^+^; 369.1548 [2M+H]^+^; 202.1075 [M+NH_4_]^+^; 207.0629 [M+Na]^+^; 388.1283 [4M+H+K]^2+^; 139.0767 [M-H-CO_2_]^−^; 167.0703 [M+H-H_2_O]^+^; 121.0647 [M+H-H_2_O-HCOOH]^+^; 122.0681 ^13^C[M+H-H_2_O-HCOOH]^+^	II [15,22]
C163	Hydroxy-DEDA	C_9_H_12_O_5_	222	199.0611 [M-H]^−^; 200.0644 ^13^C[M-H]^−^; 201.0758 [M+H]^+^; 218.1024 [M+NH_4_]^+^; 183.0652 [M+H-H_2_O]^+^	II [13]
C164	DEDA hydrated (I)	C_9_H_14_O_5_	115	201.0768 [M-H]^−^; 203.0915 [M+H]^+^; 220.1181 [M+NH_4_]^+^	II [15]
C165	DEDA hydrated (II)	C_9_H_14_O_5_	124	201.0768 [M-H]^−^	III
C166	DEDA hydrated (III)	C_9_H_14_O_5_	135	201.0768 [M-H]^−^; 403.161 [2M-H]^−^; 203.0914 [M+H]^+^; 220.1181 [M+NH_4_]^+^	II [15]
C167	DEDA hydrated (IV)	C_9_H_14_O_5_	167	201.0769 [M-H]^−^; 202.0802 ^13^C[M-H]^−^; 203.0915 [M+H]^+^	II [15]
C168	DEDA ester (I)	C_10_H_14_O_4_	262	197.082 [M-H]^−^; 395.1713 [2M-H]^−^; 151.0403 [M-H-C_2_H_6_O]^−^	III
C169	DEDA ester (II)	C_10_H_14_O_4_	327	197.0819 [M-H]^−^; 199.0965 [M+H]^+^	III
C170	DEDA ester (III)	C_10_H_14_O_4_	343	199.0965 [M+H]^+^	III
C171	DEDA alditol (I)	C_15_H_24_O_9_	194	347.1348 [M-H]^−^; 366.1761 [M+NH_4_]^+^	III
C172	DEDA alditol (II)	C_15_H_24_O_9_	205	347.1348 [M-H]^−^; 349.1496 [M+H]^+^; 366.1762 [M+NH_4_]^+^	III
C173	DEDA alditol (III)	C_15_H_24_O_9_	222	348.1383 ^13^C[M-H]^−^; 695.277 [2M-H]^−^; 349.1496 [M+H]^+^; 366.1761 [M+NH_4_]^+^; 367.1797 ^13^C[M+NH_4_]^+^	III
C174	DEDA alditol (IV)	C_15_H_24_O_9_	228	347.1346 [M-H]^−^; 348.1381 ^13^C[M-H]^−^; 695.2768 [2M-H]^−^; 696.2802 ^13^C[2M-H]^−^; 547.2035 [add]^−^; 349.1495 [M+H]^+^; 350.1529 ^13^C[M+H]^+^; 366.176 [M+NH_4_]^+^; 367.1795 ^13^C[M+NH_4_]^+^; 331.139 [M+H-H_2_O]^+^	III
C175	DEDA alditol (V)	C_15_H_24_O_9_	246	393.1403 [M-H+HCOOH]^−^; 366.1761 [M+NH_4_]^+^	III
C176	Loganin	C_17_H_26_O_10_	282	435.1508 [M-H+HCOOH]^−^; 436.1544 ^13^C[M-H+HCOOH]^−^; 391.16 [M+H]^+^; 408.1866 [M+NH_4_]^+^; 229.1071 [M+H-hexose]^+^; 211.0966 [M+H-hexose-H_2_O]^+^	II [23]
C177	Loganin aglycone (I)	C_11_H_16_O_5_	301	229.1071 [M+H]^+^; 230.1105 ^13^C[M+H]^+^; 246.1337 [M+NH_4_]^+^; 274.1651 [M+C_2_H_8_N]^+^; 211.0966 [M+H-H_2_O]^+^; 197.0809 [M+H-CH_3_OH]^+^	II [23]
C178	Loganin aglycone (II)	C_11_H_16_O_5_	327	229.1071 [M+H]^+^; 274.1651 [M+C_2_H_8_N]^+^; 211.0965 [M+H-H_2_O]^+^; 197.0808 [M+H-CH_3_OH]^+^; 179.0703 [M+H-H_2_O-CH_3_OH]^+^	II [23]
C179	Loganin aglycone (III)	C_11_H_16_O_5_	335	229.1071 [M+H]^+^; 199.0965 [M+H-CH_2_O]^+^	II [23]
C180	Loganin aglycone (IV)	C_11_H_16_O_5_	380	229.1072 [M+H]^+^; 211.0966 [M+H-H_2_O]^+^	III
C181	Hydrated product of loganin	C_17_H_28_O_11_	210	407.1558 [M-H]^−^; 408.1592 ^13^C[M-H]^−^; 357.1193 [frag]^−^; 426.197 [M+NH_4_]^+^; 229.1072 [M+H-hexose-H_2_O]^+^	III
C182	Hydroxytyrosol derivative 01	C_8_H_10_O_3_	118	155.0702 [M+H]^+^	III
C183	Hydroxytyrosol derivative 02	C_19_H_28_O_12_	211	447.1508 [M-H]^−^; 466.1918 [M+NH_4_]^+^	III
C184	Hydroxytyrosol derivative 03 (I)	C_17_H_24_O_6_	361	323.15 [M-H]^−^	III
C185	Hydroxytyrosol derivative 03 (II)	C_17_H_24_O_6_	375	323.15 [M-H]^−^; 325.1647 [M+H]^+^	III
C186	Hydroxytyrosol derivative 04	C_22_H_24_O_11_	370	463.1246 [M-H]^−^	III
C187	Hydroxytyrosol derivative 05	C_15_H_20_O_5_	373	279.1237 [M-H]^−^; 280.127 ^13^C[M-H]^−^; 559.2549 [2M-H]^−^; 315.1007 [M+Cl]^−^; 325.1292 [M-H+HCOOH]^−^; 143.0715 [M-H-OHTYR]^−^	III
C188	Hydroxytyrosol derivative 06	C_25_H_26_O_8_	450	453.1556 [M-H]^−^	III
C189	Hydroxytyrosol derivative 07	C_21_H_30_O_7_	540	393.1919 [M-H]^−^; 394.1953 ^13^C[M-H]^−^; 257.1395 [M-H-OHTYR]^−^	III
C190	DEDA derivative 01	C_23_H_32_O_11_	225	483.1873 [M-H]^−^; 484.1907 ^13^C[M-H]^−^	III
C191	DEDA derivative 02	C_16_H_22_O_9_	256	357.1192 [M-H]^−^; 358.1225 ^13^C[M-H]^−^; 359.134 [M+H]^+^; 376.1604 [M+NH_4_]^+^	III
C192	DEDA derivative 03 (I)	C_23_H_32_O_12_	224	499.1822 [M-H]^−^	III
C193	DEDA derivative 03 (II)	C_23_H_32_O_12_	299	518.223 [M+NH_4_]^+^	III
C194	DEDA derivative 04 (I)	C_18_H_24_O_8_	400	367.1397 [M-H]^−^	III
C195	DEDA derivative 04 (II)	C_18_H_24_O_8_	414	367.1396 [M-H]^−^	III
C196	DEDA derivative 05	C_18_H_24_O_7_	426	351.1448 [M-H]^−^	III
C197	DEDA derivative 05 linked to hydroxytyrosol	C_26_H_32_O_9_	488	487.1974 [M-H]^−^	III
C198	Elenolic acid derivative 01	C_6_H_10_O_2_	75	159.0665 [M-H+HCOOH]^−^; 132.1018 [M+NH_4_]^+^	III
C199	Elenolic acid derivative 02 (I)	C_11_H_12_O_7_	57	274.0925 [M+NH_4_]^+^	III
C200	Elenolic acid derivative 02 (II)	C_11_H_12_O_7_	144	255.0509 [M-H]^−^	III
C201	Elenolic acid derivative 03	C_26_H_36_O_13_	407	555.2084 [M-H]^−^	III
C202	Elenolic acid derivative 04	C_23_H_26_O_12_	410	493.1352 [M-H]^−^; 495.1497 [M+H]^+^	III

Abbreviations: C, compound number; DEDA, decarboxymethyl elenolic acid dialdehyde; LI, level of identification (according to [9,24]); ref, reference; RT, retention time (in seconds).

### 2.2. Targeted Metabolomics

A targeted method was developed for the quantitative determination of a range of identified compounds occurring in the analyzed products. The separation method was developed based on a previously published method [25]. The gradient was optimized to allow the separation of isomeric compounds, although in some cases this could not be achieved. This methodology included both a diode array detector (DAD) and a single quadrupole mass spectrometer (MS). When possible, quantification was performed using the corresponding pure standard. When this was not feasible, the chemical standard most similar to the compound of interest was used (as indicated in the methodology section). Tyrosol was also added in the quantification method, although it was not identified through the untargeted analysis, because we expected to find this compound in these samples but it is already known that it is usually not well resolved with the generic methods used in untargeted LC-MS methods. Table 2 shows the methodological data concerning the quantification, both indicating the detector used (DAD or MS), the ranges covered (LDR, linear dynamic range), the values of the curves (slope, offset and coefficient of determination), and the values of the method validation. The calibration curves for each standard were linear over a broad concentration range and with a coefficient of determination (R^2^) >0.972. The widest range of linearity was for verbascoside. The precision of the instrument was assessed by analysing the sample “G” multiple times, both intra-day and inter-day. We selected this sample class because it was the one in which most of the compounds were quantified in the highest values. Samples were prepared and analyzed by the same operator and results were evaluated using the relative standard deviation (% RSD) of the peak areas. Intra-day and inter-day precision were assessed by injecting the same sample either on the same day or on 5 consecutive days, respectively. Repeatability was evaluated by the same operator preparing different samples (n = 7) and injecting them on the same day. As expected, intra-day variability was lower than inter-day variability for most of the compounds. All values were <15%, with the exception of the inter-day variability for citric acid and 3,4-dihydroxyphenylglycol.

A total of 26 compounds were included in the targeted analyses. The quantitative data of analyzed samples are provided in Table 3. The ranges of concentrations varied widely between samples and among the different compounds. Total quantified content ranged from 0.25 to 172.81 mg/g, being ≤1 mg/g for buds-based supplements and for product “O”, between 2 and 4 mg/g for liquid extract products and higher values for dry extracts. Quinic acid, sorbitol and tyrosol were the only compounds with quantifiable levels within buds-based products. Among the liquid or dry extract supplements, the only compounds that were observed in quantifiable levels were hydroxytyrosol and verbascoside.

### 2.3. Data Visualization and Analysis

**Figure 1:**
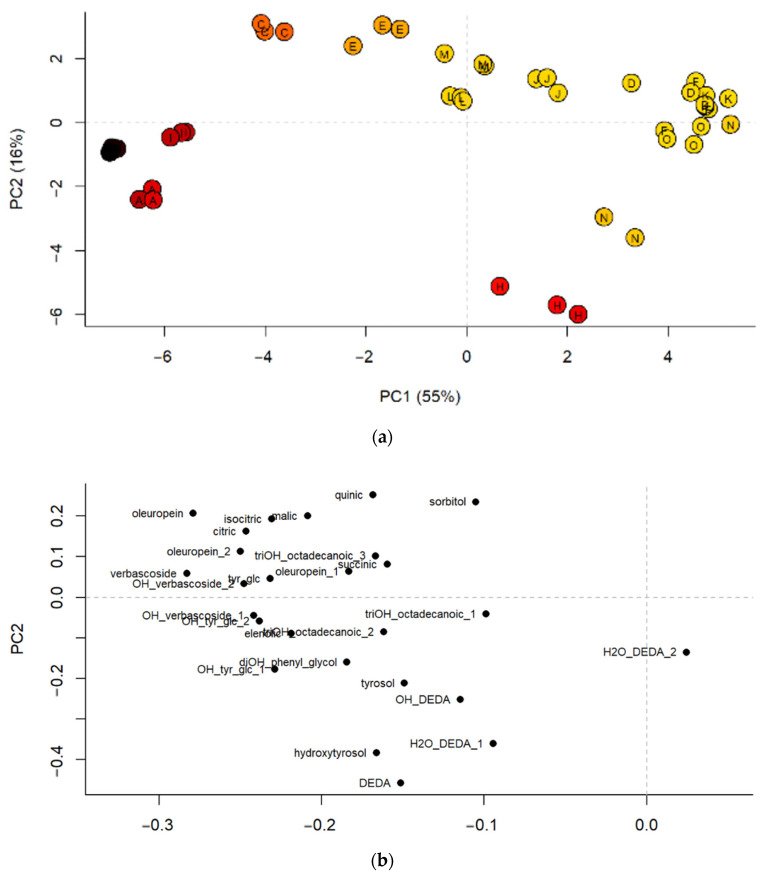
Principal component analysis (PCA) score (**a**) and loading (**b**) plot from targeted data. Samples in the score plot (**a**) are colored according to the total amount of quantified compounds, with a scale from black to yellow through red, in descending order.

Unsupervised principal component analysis (PCA) was applied based on the quantified compounds to obtain an overview of the potential similarities and differences among study samples (Figure 1). 

The first two components of the PCA model accounted for 71% of variance. First of all, it can be observed that, for most of products, the three replicates of each product are close to each other, indicating a correct preparation, sample analysis and data processing procedure. However, this was not the case for product “N”, for which one sample was quite far from the other two. Further inspection of the data and the chromatograms revealed that the injection of this sample probably failed, somehow. The samples were coloured according to the total amount of quantified compounds, with a scale from black to yellow through red, indicating the total amount of quantified compounds in descending order. The graph on the first two main components shows how the PC1 separates the samples mainly according to their concentration. This coincided with the fact that on the loadings plot, practically all the compounds were located on the same side of the graph, where the samples with a greater number of quantified compounds were located. In parallel, the scores plot also shows the existence of some groups of samples. It can be observed that the samples are mainly clustered according to the type of extraction, which coincides with their concentration, with the exception of sample “O”. That is, on the right side of the plot there are the products obtained by glyceric maceration of buds (K-D-F), together with the product “O”, which is a dry extract from leaves and fruit. They were the samples with the lowest amounts of quantified compounds. Products J-M-L were also clustered together in the same region of the scores plot. All of them were liquid extracts from leaves with intermediate values of quantified compounds. The rest of the samples were products obtained by dry extraction divided in further “subgroups” along the scores plot of the first two components. For on the right side there were the products “C” and “E” which were dry extracts from olive leaves (with or without fruit), but also including other plant extracts (see Table 4 for further details). On the left side of the plot there was a cluster made of products “G”, “A” and “I”, that were those with the highest concentrations of quantified compounds. Finally, there were 2 other products that were not clustered together with any other one. They were products “H” and “N”. Some particularities of these products were that product “H” was the one with the highest amounts of hydroxytyrosol (Table 3), over 4 times more than the next products with the highest amounts of hydroxytyrosol (products “A” and “I”). As regards the product “N”, it contained generally very low quantities of quantified compounds, with the sole exception of hydroxytyrosol.

The PCA performed with the quantified compounds confirmed a sample arrangement very similar to that shown by the untargeted analysis (Figure S1), indicating that the compounds selected for quantification were representative of the general metabolic profile of the products under analysis.

**Figure 2:**
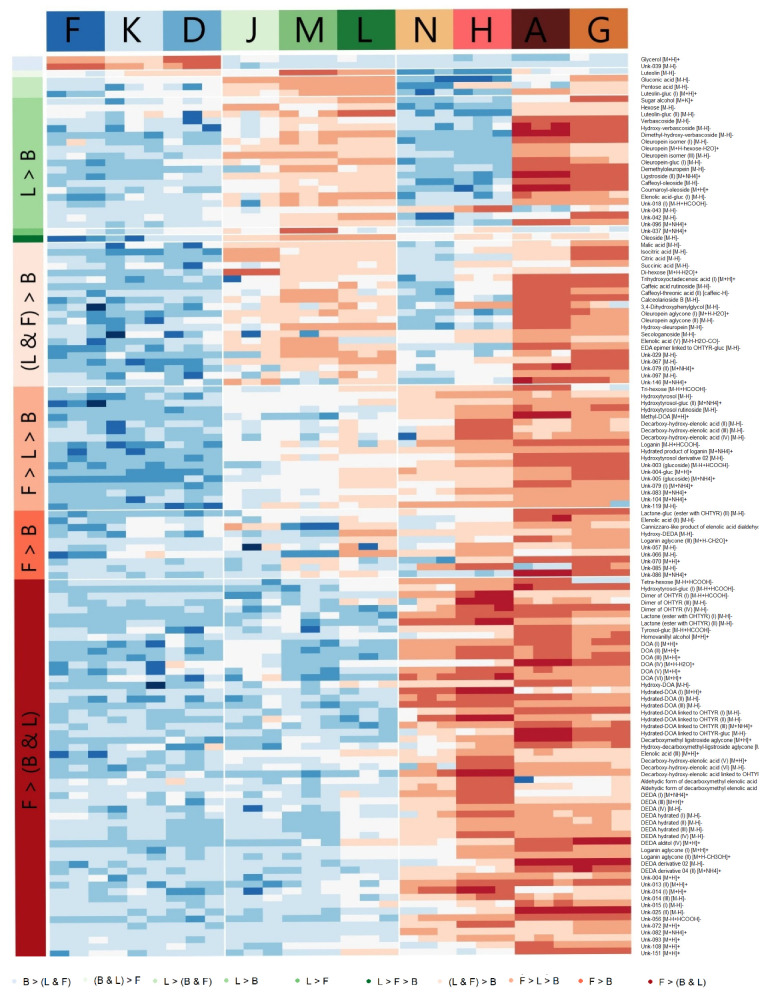
The heat map of discriminant features according to the part of the plant used was selected employing the most intensive feature of each compound. Blue and orange cells correspond to low- and high-metabolite levels, respectively. Columns are samples (codified as indicated in Table 4), and rows are compounds colored by behavior distribution among products’ classes. Product classes refer to buds-based products (B), leave-based products (L) and fruit-based products (F). The symbology of rows’ groups (e.g., “F > L > B”) indicates the behavior of the compound distribution among the products’ classes according to the Kruskal–Wallis test.

Since another factor that seems to contribute to the distribution of samples within the score plot is the part of the olive plant used, we wondered if there could be any compound peculiar to this attribute. In order to have the most homogeneous groups possible, the selected samples were K-D-F for buds (all glyceric macerates), J-M-L for leaves (all liquid extracts) and N-H-A-G for fruit (all dry extracts). A total of 357 features resulted as statistically significant, most of them being characteristic of fruit-based products (Figure 2 and Figure S2). Indeed, there were only two compounds peculiar to the buds-based products. They were glycerol and unknown 039. We suspect that the glycerol comes from the extraction process and not from the matrix. On the other hand, we could not find any specific compound for leave-based products. Among their characteristic compounds, there were high levels of luetolin for products “M” and “L”, but not for the “J” product; or gluconic and pentose acids, but some fruit-based products also presented considerable amounts of them. As already mentioned, most of the compounds that presented statistically significant differences according to the part of the plant used were characteristic of fruit-based products. Many of these compounds were of the family of phenyl alcohols and secoiridoids, such as hydroxytyrosol, decarboxymethyl oleuropein aglycone or decarboxymethyl elenolic acid dialdehyde. Figure S3 shows the box-plots of the examples just mentioned. However, in the light of these results it is not possible to know to what extent the observed differences are related to the part of the plant used or to the extraction method. It is also important to keep in mind that the small sample size available (3–4 products/group) and the high heterogeneity observed even within a group could be making it difficult to find highly specific markers for a particular class of products.

Even so, we wanted to go one step further to try to extract as much information as possible from the acquired data. First, we asked ourselves to what extent the amount of compounds present in the products could be somehow associated with the part of the plant used. To do this, we first made a pie chart to visualize the proportion of compounds detected in each product based on the total sum of intensities. Since the use of one type of data or another is associated with a number of advantages and limitations, we wanted to use all available data. That is to say, the data from the targeted analysis (which presents quantitative data, but not all the compounds present), the untargeted data (which encompasses a greater number of the compounds present in the products, but with semi-quantitative data) and using only the data from the ions that had been identified in the untargeted experiment. As can be seen in Figure S4, all the data matrices used presented a similar output, which basically showed once again how the fruit-based products were those with a higher amount of compounds present, while the buds-based products showed much lower values and the products based on a mixture of fruit and leaves were in an intermediate situation between the fruit-only and leaf-only products.

Finally, we wondered if the proportion of phenolic and non-phenolic compounds might also be varying according to the type of plant part used. So, we made pie charts again, but this time one graph per product showing the proportion of phenolic or non-phenolic compounds (Figure S5). Again, a difference could be clearly seen according to the part of the plant used. While buds-based products presented a higher proportion of non-phenolic compounds, the fruit-based products were those with higher proportions of (poly)phenolic compounds, with the leaf-based products presenting an intermediate situation, and the fruit- and leaf-based products an intermediate situation between the fruit only products and the leaf only products. Again, this observation was very similar between the targeted and untargeted data.

Lastly, the wide heterogeneity of the products included in this study in terms of pharmaceutical form, recommended dose, etc., must also be taken into account (Table 4). It is precisely for this reason that we also intended to examine and interpret the results obtained transforming the quantified quantities per gram of product by considering the recommended daily intake by the respective producers (indicated in Table 4). These results showed how for some products, in which the amount of some compounds was of little relevance (when compared to that present in other products), the situation changed radically (Table S3) when the variable “amount of supplement taken daily” was considered. Hydroxytyrosol is an illustrative example of this phenomenon. As already commented and deciphered by the results from targeted analysis, considering initially only the compositional profile of the different products, the supplement “H” had the highest values, over 4 times more than the next one in terms of mg of hydroxytyrosol per gram of product. However, it is with supplements “J” and “N” that the highest levels of intake are reached (around 10 mg/day). More specifically, the values changed most markedly with integrator “J”, for which the intake dose is much higher (70 mL, which in terms of weight in g corresponds to 72.80 g) than for the other products, for which the recommended intake dose is always below 3 g. Only 4 products (“J”, “N”, “I” and “A”) reached the EFSA recommended daily dosage of hydroxytyrosol (5 mg/day) by themselves, with values ranging between 6 and 11 mg/day, considering recommended doses and quantified values. There are two other products (“C” and “E”) which, while providing less than 5 mg/day of hydroxytyrosol as such, do exceed this value in the form of oleuropein, which is also taken into account when evaluating the amount of hydroxytyrosol provided since it is hydrolyzed to hydroxytyrosol.

## 3. Discussion

A comprehensive high-performance liquid chromatography–high resolution mass spectrometry (HPLC-HR-MS) fingerprinting of different commercially available OBDS showed that they are characterized as being highly complex mixtures. The most abundant signals were subjected to annotation processes. They corresponded to a large number of phenolic compounds typical of the olive tree. The characteristic metabolites of this plant include compounds such as tyrosol and hydroxytyrosol, both in their free form and esterified to form secoiridoid structures (such as ligstroside, oleuropein and analogues), as well as their catabolites produced by oxidation, hydrolysis and/or hydration reactions, as well as loss of carboxylic and carboxymethyl groups [15]. The dominant proportion of compounds identified in this work belongs to the subclass of secoiridoids. The high resolving power of the mass analyzer allowed the identification and classification of most of these compounds based on the observation of specific and characteristic fragments and/or neutral losses. Other compounds, not specific markers of the olive tree were also identified. These were mainly sugars and organic acids. The analysis of the distribution of compounds in the different products grouped by the part of the plant used showed that most of the discriminant compounds were found at higher concentrations in fruit-based products. The ratio of phenolic vs non-phenolic compounds differed according to the part of the plant used, with buds-based products being those with the highest proportion of non-phenolic compounds, and the fruit-based products the ones whose composition consisted mostly of (poly)phenolic compounds. The quantification of compounds for which standards were available confirmed the huge variations among different products, providing a range of their concentration within each group of products (buds, leaf and fruit).

Quantifiable levels of hydroxytyrosol were only observed in non-buds-based supplements. The quantified range was between 0.05 and 88 mg/g. The recommended daily intake dose varies significantly and largely modulates the amount provided daily. Regarding the toxicity of hydroxytyrosol, few studies have been conducted on this topic [26]. A reference value can only be found in a 2006 revision [27], illustrating the results of studies conducted on the toxicity of olive extract. It is reported here as the daily intake of hydroxytyrosol and its precursors is considered safe up to levels greater than 1200 mg/day (1.2 g). The intake of supplement “J”, which provides the highest amount of hydroxytyrosol when considering the recommended dose of consumption, leads to a dietary intake 100 times lower than the proposed safety limit. Additionally, this compound can be considered safe, considering that no study has ever highlighted allergies, or interactions with the diet, due to the intake of this compound [3,26].

This work demonstrates the high complexity and heterogeneity in the composition of the OBDS that are available in the market and easily accessible to consumers and also that high-resolution mass spectrometry with accurate mass measurements provides valuable information for compound identification supplying precious data about structural information. It also highlights the need to better classify the various OBDS into homogenous subgroups, providing compositional data that can assist in building models of classification. These results show that we are faced with a myriad of products, often indiscriminately classified as functional foods, nutraceuticals or food supplements, and marketed claiming the most diverse biological activities, confusing the consumer. The evidence reported in this work suggests that it is of the utmost importance to define a proper and unequivocal classification of olive-based dietary supplements, separating those obtained from different tissues.

## 4. Materials and Methods

### 4.1. Solvents and Standards

HPLC-grade methanol, acetonitrile and formic acid were purchased from Sigma-Aldrich (St. Louis, MO, US). A Milli-Q system from Millipore (Bedford, MA, USA) was used for purifying demineralized water. The PVDF (polyvinylidene fluoride) syringe filters were also supplied by Millipore. The standards used were as follows: citric acid, isocitric acid, mannose, dulcitol, oleuropein, raffinose, stachyose, salidroside, sorbitol, tyrosol and 3,4-dihydroxyphenylglycol purchased from Sigma-Aldrich (St. Louis, MO, US); malic acid, fructose, glucose and sucrose purchased from Carlo Erba (Milan, Italy); succinic acid, galactinol, galactose and lactose purchased from Honeywell Fluka (Milan, Italy); quinic acid purchased from Roth (Karlsruhe, Germany); and hydroxytyrosol and verbascoside purchased from Extrasynthese (Genay Cedex, Lyon, France).

### 4.2. Study Samples

The samples subjected to analysis were OBDS acquired in various pharmacies and herbalists in Italy, or bought through the internet. The shared aspect among all of them was the use of the olive tree plant as key raw material, regardless of the part used. The specific characteristics of these samples have been collected in Table 4, in which the commercial name of the products has been coded with a randomly attributed alphabetical letter.

### 4.3. Sample Preparation

The preparation phase involved different procedures, depending on the physical state of the samples, whether liquid or solid. Each sample was prepared and analyzed in triplicate.

For liquid samples, 500 mg were weighted and brought to 5 mL with a solution of MeOH/H_2_O MilliQ 20:80 (v/v). To ensure complete solubilization, the flasks were transferred to an ultrasonic bath for 10 min. Later, the solution was filtered with 0.22 μm filters into HPLC vials. An additional, more diluted solution of each sample was prepared at a concentration of 2 g/L.

For solid samples, 500 mg were also weighted. In this case, 2 mL of MeOH/H_2_O MilliQ 20:80 (v/v) were added and shaken for 5 min. Later, the flasks were transferred to an ultrasonic bath for 10 min. At this point, they were processed by centrifugation at 4000 g for 5 min at 5 °C. The aqueous fraction was then taken away from the solid component and transferred to a 5 mL flask. The precipitate, and the possible oily fraction, were taken with 2 mL of MeOH/H_2_O MilliQ 20:80 (v/v), and then the extraction procedure was repeated in the same way as described above, for a second time. The extract was brought to volume with MeOH/H_2_O MilliQ 20:80 (v/v). After manual stirring, the solution was then transferred into HPLC vials, after a double filtration with 0.45 μm and 0.22 μm filters. Also in this case, a more diluted sample (2 g/L) was prepared.

All samples were mixed using a vortex before being subjected to instrumental analysis.

### 4.4. Untargeted Metabolomics

#### 4.4.1. Chromatographic and Mass Spectrometry Conditions

All samples (2 g/L) were analyzed using a Dionex UltiMate 3000 HPLC system coupled with a hybrid linear ion trap Fourier transform (LTQ FT) Orbitrap mass spectrometer (Thermo Fisher, Bremen, Germany).

The chromatographic system was equipped with an autosampler and quaternary gradient HPLC pump. The column used was a Kinetex C18 (150 mm × 2.1 mm I.D., particle size 2.6 μm, Phenomenex Torrance, CA, USA), equipped with the corresponding Phenomex C18 4 × 2.00 mm pre-column, maintained at 40 °C. The mobile phase used was Milli-Q water with 0.1% formic acid (solvent A) and acetonitrile with 0.1% formic acid (solvent B). The following gradient was adopted: 0–1 min 5% B; 1–7 min linear gradient 5–45% B; 7–8.5 min linear gradient 45–80% B; 8.5–10.5 min maintaining 80% B; 10.5–11% min linear gradient from 80% B to 5% B; 11–12 min maintaining 5% B. The flow rate was 350 μL/min, and the injection volume 10 μL.

The Orbitrab LTQ was equipped with an electrospray ionization (ESI) source, and operated in both positive and negative ionization modes, as well as using both the full scan (FS) and the data dependent acquisition (DDA) modes (a total of four injections per sample were done). The operating conditions of the mass spectrometer in negative (and positive) mode were: source voltage 3.5 kV (5.0 kV), heated capillary temperature 320 °C and capillary voltage −30 V (30 V). In the LTQ component of the instrument, nitrogen was used as both heated gas (70 U) and auxiliary gas (30 U), and helium as damping gas. Mass calibration was carried out just before starting the analysis using the manufacturer’s calibration solution, and ensuring a mass accuracy of <5 ppm in external calibration mode. In the FS method the mass range was from 80 to 800 Da at a mass resolution of 30,000 FWHM (m/z 400), in centroid mode. In the DDA method, during the chromatographic run both the FS and the MS/MS spectra of the three ions with the highest intensities of each FS were acquired. The resolution power for the MS/MS scans was 7500. The ions produced were generated in the LTQ trap at 35 eV collision energy using an isolation width of 1 Da.

At the beginning of the analytical sequence, 10 quality controls (QC) were injected in order to stabilize the system. The QC was a mixture obtained by taking a 30 μL aliquot from each sample tested. During the sequence a solvent (MeOH/H_2_O MilliQ 20:80 (v/v)) followed by two QCs were added every 10 samples, to verify the stability and performance of the analytical system in terms of retention time, mass accuracy, signal strength, and possible analytical variations.

#### 4.4.2. Data Processing

Raw files were converted into mzXML format using the MSconvert module of ProteoWizard software. Subsequently, files from FS experiments were processed using the version 3.11.6 of XCMS package [28] in the R platform and employing the parameters specified in Table S4. Data were processed separately for negative and positive modes.

#### 4.4.3. Compound Identification

Detected features were grouped (separately for each ionization mode) when they presented a close RT and a high sample and peak shape correlations. Later, the relationships between the different m/z values grouped together were calculated in order to establish whether any given value could be the (de)protonated ion or one of its isotopes, adducts or fragments. Information provided by adduct formation in the complementary ionization mode was also used to decipher which m/z value could be associated with the (de)protonated ion. Once the m/z value of the (de)protonated ion was known, this value, together with its isotopic pattern and MS^2^ spectra, was used to associate it with a molecular formula composed using the software Sirius [29]. The MS^2^ spectrum used was the one resulting from merging three MS^2^ spectra (when available) using the “mergeMS2spectra” function of “CluMSID” R package [30], selecting those fragments with a relative intensity >1%. Retrieved formulas were searched in the relevant bibliography, as well as in public databases (such as FooDB, Phytohub, or PhenolExplorer) to see if they matched any potential compound. In the affirmative case, the theoretical MS/MS fragmentation pattern was searched in the scientific bibliography and in public mass spectral databases (i.e., mzCloud, Metlin, and MassBank) in order to compare it with the experimental one. When this comparison matched and the corresponding analytical standard was available commercially (i.e., standards mentioned in the Section 4.1), it was purchased in order to analyze it under the same experimental conditions and verify if the RT and experimental fragmentation pattern were the same or not. In the affirmative case, and following the recommendations of the Metabolomics Society [24], they were assigned to level 1, while those compounds for which the analytical standard was not available but the theoretical MS/MS fragmentation pattern matched with that obtained experimentally in the study samples were assigned to level 2. Level 3 included those compounds for which the MS/MS comparison was done using in silico tools, such as MetFrag, or when the class of compounds could only be described on the bases of the observed fragmentation pattern, either by the observation of a specific ion or some particular neutral loss in the MS/MS spectra.

### 4.5. Targeted Metabolomics

Compounds identified in the untargeted approach for which the corresponding standard was available were included in the targeted analysis (Table S5). Quantification was undertaken without considering the chiral configuration since the column used was not specific for this characteristic. Additional compounds that were included in the method, but for which the analytical standard was not available, were expressed as oleuropein, with the exception of hydroxytyrosol glucoside which was expressed as hydroxytyrosol, hydroxyverbascoside that was expressed as verbascoside, and trihydroxyoctadecanoic acid that was expressed as isocitric acid.

#### 4.5.1. Chromatographic and Mass Spectrometry Conditions

The targeted metabolomic analysis was conducted using the Waters 2695 HPLC system (Milliford, CT, USA) equipped with the Waters 2996 DAD ultraviolet–visible (UV-Vis) detector (Milliford, CT, USA) and single quadrupole mass spectrometer with ESI Acquity qDa ionization system (Waters, Milliford, CT, USA). The method used, with the necessary adaptations for the current work, was taken from the study by M. Ricciutelli et al. (2017) [25], in which a new method for the quantification of phenolic compounds contained in extra virgin olive oil (EVOO) was developed and validated.

The chromatographic analysis was conducted using the Synergi Polar-RP (reverse phase) column, 250 × 4.6 mm 4 μm (Phenomenex, Cheshire, UK) equipped with the respective pre-column (4 × 2.00 mm). The separation was conducted in 80 min, keeping the column thermostated at 35 °C. The mobile phase consisted of 1% formic acid in water MilliQ (solvent A) and methanol/isopropanol 90:10 v/v with 0.1% formic acid (solvent B). The flow used was 1 mL/min and the injection volume 10 μL. The gradient used was modified from what was reported in the reference study. In particular there are variations in the first part, where an isocratic phase of solvent A, from 0 min to 40 min minute, was introduced. This variation aimed, as far as possible, at obtaining a better separation of polar compounds. The gradient used was the following: 0–40 min linear gradient 0–45% B; 40–60 min linear gradient 45–60% B; 60–70 min linear gradient 60–95% B; 70–73 min maintaining 95% B; 73–7.1 min linear gradient from 95% B to 0% B; 73.1–80 min maintaining 0% B.

The UV-VIS spectrum was recorded from 200 nm to 600 nm, with detection at 280 nm and 330 nm. The mass spectrometer was used with a spray voltage set to 0.8 kV in negative and positive mode. The probe temperature was set to 600 °C, and the cone voltage was set to 15V. The spectra were then collected in a mass range m/z 100–1250 Da.

#### 4.5.2. Method Development and Validation

The LDR, i.e., the range of concentrations in which the response was linear, was defined in solvent because the matrices of the present study were extremely heterogeneous. We then evaluated the limit of quantification (LOQ), which represents the lowest quantifiable metabolite concentration. For the method development a total of 12 points of the calibration curve ranging from 0.02 ppm to 100 ppm were analyzed, with the exception of hydroxytyrosol, tyrosol and verbascoside for which the calibration curve was extended by an additional 5 points to 1500 ppm. For each calibration curve, the RDL and LOQ was evaluated. Subsequently, the curve was rebuilt and the correlation coefficient calculated. The type of dilution (i.e., 100 g/L or 2 g/L) was used according to the observed concentration of each compound in each sample and the LDR, preferably selecting that of 100 g/L.

The proposed method was validated in terms of intra-day repeatability, inter-day variability, and operator reproducibility. To conduct these analyses, the sample coded with the letter G was chosen, since it was the one that had the highest number of compounds identified (see Section 2). To assess intra-day repeatability, the same solution of sample G was analyzed 7 times consecutively, whereas the inter-day variability was evaluated by the injection of the same sample G during 5 consecutive days. Finally, to assess reproducibility, a parameter that evaluates the variability induced by the operator, 7 different solutions of sample G were set up and injected on the same day. The three parameters were expressed in terms of percentage of relative standard deviation (RSD%).

### 4.6. Data Analysis and Visualization

Principal component analysis (PCA) was used to gain an overview of the obtained data, which had previously been log-transformed and Pareto-scaled. Missing values were replaced by a random very low value. R software was used to perform and visualize these analyses.

For the differential abundance analysis according to the part of the plant used, a Kruskal–Wallis test was performed and the obtained p-values were corrected using the Benjamini–Hochberg (BH) method. Adjusted *p*-values < 0.05 were considered statistically significant. In order to gain a visual overview of the results, the relative abundance of the discriminant features was represented through a heat map employing the “heatmap.2” function of the “gplots” R package. For comparative analysis across different ion peak areas, data was log-transformed and Pareto-scaled. In this plot, samples were placed in columns ordered by class, and features in rows ordered according to the behavior of distribution. Color represents relative intensity, using orange to represent high abundance and blue for low abundance.

## Figures and Tables

**Table 2:** Parameters of the calibration curves used for quantification and results of method validation.

Compound	Detector	LDR (mg/L)	Curve			Intra-Day (% RSD)	Inter-Day (% RSD)	Repeatability (% RSD)
			Intercept	Slope	R^2^			
Quinic acid	MS	10–100	2,284,463	146,048	0.989	6.78	10.87	5.38
Malic acid	MS	10–100	1,649,636	32,379	0.992	7.51	14.90	6.68
Isocitric acid	MS	0.2–50	226,679	204,797	0.993	7.74	12.54	9.38
Citric acid	MS	5–100	1,484,339	213,678	0.995	7.00	16.25	5.90
Succinic acid	MS	5–100	769,682	66,403	0.991	3.55	14.35	3.09
Sorbitol	MS	2–100	968,312	88,135	0.972	4.59	13.94	2.07
3,4-Dihydroxy-phenylglycol	MS	0.2–50	262,381	91,393	0.986	8.99	18.55	2.92
Hydroxytyrosol	MS	2–100	2,082,530	253,685	0.988	10.41	5.41	8.30
Oleuropein	MS	0.5–50	1,004,848	695,049	0.991	4.00	5.25	4.04
Tyrosol	DAD	2–1505	−16,214	5499	1.000	1.52	3.65	8.19
Tyrosol glucoside	DAD	5–100	−111	2334	1.000	2.26	2.85	9.81
Verbascoside	DAD	0.5–1536	−23,591	14,737	1.000	0.77	1.81	9.48

LDR, linear dynamic range; RSD, relative standard deviation.

**Table 3:** Range of concentrations (mg/g) of the quantified compounds in study samples.

Compound	K	D	F	J	M	L	C	O	E	I	N	H	A	G
Quinic acid	0.19–0.21	0.57–0.81	0.11–0.34	0.35–0.57	1.11–1.51	0.73–0.77	11.52–15.3	<LOQ	0.64–0.67	0.45–0.7	<LOQ	<LOQ	0.4–0.65	74.84–79.94
Malic acid	<LOQ	LOQ–0.14	<LOQ	0.18–0.22	0.75–0.82	0.48–0.54	2.86–3.49	<LOQ	0.95–1.3	0.39–0.41	<LOQ	<LOQ	0.21–0.39	19.7–22.88
Citric acid	<LOQ	ND–<LOQ	<LOQ	0.85–0.89	0.27–0.27	0.3–0.34	1.08–1.2	<LOQ	0.58–0.7	0.57–0.59	<LOQ	LOQ–0.06	0.4–0.45	19.19–21.08
Isocitric acid	ND	ND–<LOQ	ND–<LOQ	0–0.01	0.06–0.06	0–0	0.12–0.2	<LOQ	0.01–0.02	0.06–0.07	<LOQ	<LOQ	0.02–0.04	0.3–0.32
Succinic acid	<LOQ	<LOQ	<LOQ	<LOQ	0.07–0.09	<LOQ	0.21–0.28	<LOQ	<LOQ	0.55–0.62	<LOQ	<LOQ	<LOQ	0.5–0.54
Sorbitol	0.14–0.14	0.13–0.24	0.1–0.22	0.08–0.1	0.11–0.13	0.49–0.49	0.87–0.93	0.08–0.09	0.06–0.07	0.7–0.77	<LOQ	<LOQ	0.08–0.1	2–2.02
Trihydroxyoctadecenoic acid (I)	ND	ND–<LOQ	ND–<LOQ	ND	ND	ND	ND	ND	ND–<LOQ	ND	ND	ND	ND–0.03	0.04–0.05
Trihydroxyoctadecenoic acid (II)	ND–<LOQ	ND–<LOQ	ND–<LOQ	<LOQ	<LOQ	0.01–0.01	ND–<LOQ	ND–<LOQ	<LOQ	0–0.01	ND	ND	0.01–0.02	0–0.01
Trihydroxyoctadecenoic acid (III)	ND–<LOQ	ND–<LOQ	<LOQ	<LOQ	0.01–0.02	0.03–0.03	ND–0.02	0.01–0.02	0.05–0.05	0.18–0.22	ND	ND	0.01–0.01	0.03–0.05
Verbascoside	ND	ND–0.03	ND–0.02	0.02–0.04	0.14–0.16	0.09–0.1	2.8–3.05	ND–0.02	0.78–0.81	0.75–3.7	ND–0.02	ND–0.03	47.43–51.19	16.96–18.26
Hydroxy-verbascoside (I)	ND	ND	ND	ND	ND–0.02	ND	0.06–0.07	ND	0.15–0.16	0.07–0.18	ND	ND–0.04	2.99–3.1	0.75–0.88
Hydroxy-verbascoside (II)	ND	ND	ND	ND	ND–0.03	ND	0.38–0.43	ND	0.06–0.06	0.05–0.12	ND	ND	3.09–3.17	0.75–0.83
3,4-Dihydroxy-phenylglycol	<LOQ	<LOQ	<LOQ	0.07–0.08	0.01–0.02	0.11–0.11	0.01–0.02	<LOQ	ND–<LOQ	0.11–0.13	<LOQ	0.12–0.14	0.3–0.38	0.1–0.1
Hydroxytyrosol	<LOQ	<LOQ	<LOQ	0.14–0.16	0.2–0.27	0.05–0.06	0.13–0.17	0.09–0.13	LOQ–0.06	17.15–21.46	5.99–8.82	74.83–88.2	14.94–20.06	9.14–10.14
Hydroxytyrosol glucoside (I)	ND–<LOQ	<LOQ	<LOQ	LOQ–0.02	<LOQ	<LOQ	0.5–0.64	<LOQ	0.12–0.17	0.58–0.66	LOQ–0.47	0.17–0.2	1.71–3.03	0.55–0.75
Hydroxytyrosol glucoside (II)	<LOQ	<LOQ	<LOQ	LOQ–0.02	0.07–0.1	0.2–0.21	2.2–2.39	<LOQ	0.18–0.26	0.78–0.92	LOQ–0.17	0.08–0.12	1.38–2.05	0.56–0.69
Tyrosol	0.04–0.04	0.04–0.05	0.04–0.04	ND	0.03–0.05	0.05–0.06	ND–0.21	0.07–0.08	ND–0.07	2.28–2.66	ND	2.17–2.38	4.93–5.8	3.2–3.25
Tyrosol glucoside	ND	ND–<LOQ	ND–<LOQ	ND	0.33–0.37	ND	1.76–2.35	ND	0.57–0.77	0.4–0.56	ND	ND	10.73–11.98	4.64–6.4
Oleuropein	<LOQ	LOQ–0.01	<LOQ	0.13–0.14	0.42–0.61	0.37–0.39	16.49–19.22	<LOQ	15.64–17.4	71.76–79.09	<LOQ	LOQ–0.08	3.9–4.86	0.17–0.22
Oleuropein isomer (I)	<LOQ	ND–<LOQ	<LOQ	ND–<LOQ	ND–<LOQ	ND	0.01–0.05	ND–<LOQ	ND–0.08	0.21–0.28	<LOQ	ND–<LOQ	0.01–0.01	LOQ–0.01
Oleuropein isomer (II)	<LOQ	<LOQ	<LOQ	LOQ–0.01	<LOQ	<LOQ	0.72–0.79	ND–<LOQ	0.21–0.26	2.93–3.77	<LOQ	<LOQ	0.11–0.13	0.06–0.07
Elenolic acid	<LOQ	<LOQ	ND	0.01–0.01	<LOQ	0.03–0.04	<LOQ	<LOQ	<LOQ	0.1–0.11	ND–<LOQ	ND	1.21–1.37	0.41–0.45
DEDA	<LOQ	<LOQ	LOQ–0.51	<LOQ	<LOQ	<LOQ	<LOQ	LOQ–0.01	<LOQ	0.24–0.26	LOQ–0.17	2.78–3.16	0.73–0.83	1.84–2.13
Hydroxy-DEDA	<LOQ	<LOQ	<LOQ	<LOQ	<LOQ	0.19–0.21	<LOQ	<LOQ	<LOQ	<LOQ	LOQ–0.04	0.09–0.09	0.14–0.16	1.59–1.76
DEDA hydrated (I)	<LOQ	<LOQ	<LOQ	<LOQ	<LOQ	<LOQ	<LOQ	<LOQ	<LOQ	0.11–0.12	LOQ–0.12	0.94–1.33	0.09–0.1	0.06–0.09
DEDA hydrated (II)	ND–<LOQ	ND–<LOQ	ND–<LOQ	<LOQ	<LOQ	<LOQ	<LOQ	<LOQ	<LOQ	<LOQ	<LOQ	0.11–0.13	<LOQ	<LOQ

Abbreviations: DEDA, Decarboxymethyl elenolic acid dialdehyde; LOQ, limit of quantification; ND, not detected.

**Table 4:** Summary of the characteristics of the supplements included in the present study.

Product	Part Used	Pharmaceutical Form	Recommended Daily Intake Dose by the Supplier	Presence of Other Plant Extracts	Boasted Activity	Origin	Physical State	Extraction Product
K	buds	Drops	100 drops (5.28 g)	-	Hypotensive, cholesterol-lowering	Italy	liquid	glyceric macerate
D	buds	Drops	80 drops (2.13 g)	-	Protective vascular functionality (vasodilator, hypotensive); promotes correct lipid and carbohydrate metabolism	Italy	liquid	glyceric macerate
F	buds	Drops	75 drops (2.70 g)	-	Protective vascular functionality (vasodilator, hypotensive); promotes correct lipid and carbohydrate metabolism	Italy	liquid	glyceric macerate
J	leaves	Liquid	70 mL (72.8 g)	Calendula	Antioxidant, promotes correct lipid and carbohydrate metabolism, maintain normal blood pressure	Italy	liquid	liquid extract
M	leaves	Drops	75 drops (1.98 g)	Passiflora, hawthorn, pilosella, fumaria	Hypotensive	Italy	liquid	liquid extract
L	leaves	Drops	75 drops (1.98 g)	-	Promotes correct lipid and carbohydrate metabolism	Italy	liquid	liquid extract
C	leaves	Capsules	4 cps (2 g)	Hawthorn, goji	Maintain normal blood pressure	Italy	powder	dry extract
O	leaves and fruit	Raw material	400 mg (0.4 g)	-	Protective vascular functionality, anti-diabetic action	Spain	powder	dry extract
E	leaves and fruit	Capsules	2 cps (1.2 g)	Hawthorn, salvia	Maintain normal blood pressure	Italy	powder	dry extract
I	leaves and fruit	Raw material	400 mg (0.4 g)	-	Hypotensive, antioxidant, anti-inflammatory, cholesterol-lowering, anti-diabetic action	Spain	powder	dry extract
N	fruit	Capsules	2 cps (1.28 g)	-	Antioxidant, anti-aggregant, anti-inflammatory	Italy	powder	dry extract
H	fruit	Raw material	25 mg (0.025 g)	-	Antioxidant (cholesterol LDL)	Spain	powder	dry extract
A	fruit	Raw material	400 mg (0.4 g)	-	Antioxidant, protective vascular functionality	France	powder	dry extract
G	fruit	Raw material	400 mg (0.4 g)	-	Antioxidant, protective ultraviolet (UV) rays	France	powder	dry extract

## Supplementary Materials

The following are available online at https://www.mdpi.com/2218-1989/10/12/516/s1, Table S1: Unknown compounds detected in study samples, Table S2: MS/MS fragments of detected compounds by LTQ-Orbitrap, Figure S1: Scores plots of the samples in the space described by the first two principal components based on data obtained in untargeted analysis, Figure S2: Heat map of discriminant features according to the part of the plant used, Figure S3: Boxplots of selected compounds characteristic of products made from a particular part of the olive plant, Figure S4: Pie charts of the total amount of compounds by product, Figure S5: Pie charts of the proportion of phenolic and non-phenolic compounds present in each product, Table S3: Estimated daily intake (mg/day) of the quantified compounds according to the recommended dose, Table S4: Parameters used for data processing with XCMS, Table S5: Analytical standards used for the targeted analyses.
