# Supplementary material for: Exploratory Analysis of Commercial Olive-Based Dietary Supplements Using Untargeted and Targeted Metabolomics
Source: Metabolites. 2020 Dec 19;10(12):516. doi: 10.3390/metabo10120516 (PMC7766617; doi:10.3390/metabo10120516)
Supplement: Supplementary file 1 [file metabolites-10-00516-s001.pdf]

metabolites

Supplementary Material

Exploratory analysis of commercial olive-based dietary supplements using untargeted and targeted metabolomics

Mar Garcia-Aloy [1], Nelli Groff [1], Domenico Masuero [1], Mauro Nisi [2], Antonio Franco [3], Furio Battelini [2], Urska Vrhovsek [1] and Fulvio Mattivi [1,4,*]

[1] Metabolomics Unit, Food Quality and Nutrition Department, Research and Innovation Centre Fondazione Edmund Mach, San Michele all'Adige, Italy
[2] Agraria Riva del Garda S.C.A., Riva del Garda, Italy
[3] Ethifenol S.R.L., Bergamo, Italy
[4] Department of Cellular, Computational and Integrative Biology - CIBIO, University of Trento, San Michele all'Adige, Italy

Table S1. Unknown compounds detected in study samples.

C	Compound	Formula	RT	Ions	LI
C203	Unknown 001 (glucoside)	$C_{12}H_{23}NO_7$	78	294.1551 $[M+H]^+$	IV
C204	Unknown 002 (glucoside)	$C_{14}H_{26}O_8$	82	323.1704 $[M+H]^+$	IV
C205	Unknown 003 (glucoside)	$C_{13}H_{18}O_8$	96	347.0982 $[M-H+HCOOH]^-$	IV
C206	Unknown 004 glucoside	$C_{15}H_{24}O_8$	257	377.1453 $[M-H+HCOOH]^-$; 333.1546 $[M+H]^+$; 350.1812 $[M+NH_4]^+$; 153.091 $[M+H\text{-hexose-}H_2O]^+$	IV
C207	Unknown 004	$C_9H_{14}O_3$	282	171.1015 $[M+H]^+$; 172.1049 $^{13}C[M+H]^+$; 188.1283 $[M+NH_4]^+$; 153.091 $[M+H-H_2O]^+$; 111.0803 $[M+H-C_2H_4O_2]^+$	IV
C208	Unknown 005 (glucoside)	$C_{18}H_{30}O_{11}$	278	467.1769 $[M-H+HCOOH]^-$; 468.1803 $^{13}C[M-H+HCOOH]^-$; 423.1862 $[M+H]^+$; 440.2126 $[M+NH_4]^+$; 441.2163 $^{13}C[M+NH_4]^+$; 261.1335 $[M+H\text{-hexose}]^+$; 243.1228 $[M+H\text{-hexose-}H_2O]^+$	IV
C209	Unknown 006 (glucoside)	$C_{19}H_{30}O_8$	287	387.2015 $[M+H]^+$	IV
C210	Unknown 007 (glucoside)	$C_{19}H_{32}O_9$	316	403.1974 $[M-H]^-$; 422.2386 $[M+NH_4]^+$	IV
C211	Unknown 008 (glucoside) (I)	$C_{25}H_{30}O_{12}$	346	523.1808 $[M+H]^+$	IV
C212	Unknown 008 (glucoside) (II)	$C_{25}H_{30}O_{12}$	369	521.1665 $[M-H]^-$; 567.1719 $[M-H+HCOOH]^-$	IV
C213	Unknown 009 (glucoside)	$C_{25}H_{30}O_{14}$	366	553.1559 $[M-H]^-$	IV
C214	Unknown 010 (glucoside)	$C_{33}H_{40}O_{16}$	398	691.2243 $[M-H]^-$	IV
C215	Unknown 011 glucoside	$C_{27}H_{36}O_{14}$	403	583.2032 $[M-H]^-$; 584.2066 $^{13}C[M-H]^-$; 585.2085 (2)$^{13}C[M-H]^-$; 602.2443 $[M+NH_4]^+$; 603.2477 $^{13}C[M+NH_4]^+$; 423.165 $[M+H\text{-hexose}]^+$; 377.1233 $[M+H\text{-Hexose-}H_2O\text{-}C_2H_4O]^+$; 378.1265 $^{13}C[M+H\text{-Hexose-}H_2O\text{-}C_2H_4O]^+$; 225.0758 [+]; 359.1127 [+]; 539.1758 [+]	IV
C216	Unknown 011	$C_{21}H_{26}O_9$	521	421.1506 $[M-H]^-$; 422.1539 $^{13}C[M-H]^-$	IV
C217	Unknown 012 (glucoside)	$C_{34}H_{50}O_{17}$	414	729.2974 $[M-H]^-$	IV
C218	Unknown 013 (I)	$C_{13}H_{17}NO_5$	81	268.1181 $[M+H]^+$	IV
C219	Unknown 013 (II)	$C_{13}H_{17}NO_5$	174	268.1181 $[M+H]^+$	IV
C220	Unknown 014 (I)	$C_9H_{16}O_4$	112	233.1031 $[M-H+HCOOH]^-$; 189.1122 $[M+H]^+$; 206.1388 $[M+NH_4]^+$	IV
C221	Unknown 014 (II)	$C_9H_{16}O_4$	143	233.103 $[M-H+HCOOH]^-$; 189.1122 $[M+H]^+$; 234.1702 $[M+C_2H_8N]^+$	IV
C222	Unknown 014 (III)	$C_9H_{16}O_4$	169	187.0977 $[M-H]^-$	IV
C223	Unknown 014 (IV)	$C_9H_{16}O_4$	205	187.0975 $[M-H]^-$	IV
C224	Unknown 015 (I)	$C_9H_{14}O_4$	218	185.0819 $[M-H]^-$	IV
C225	Unknown 015 (II)	$C_9H_{14}O_4$	301	185.082 $[M-H]^-$; 187.0965 $[M+H]^+$	IV
C226	Unknown 016 (I)	$C_{19}H_{28}O_{10}$	263	461.1665 $[M-H+HCOOH]^-$; 434.2021 $[M+NH_4]^+$	IV
C227	Unknown 016 (II)	$C_{19}H_{28}O_{10}$	308	461.1664 $[M-H+HCOOH]^-$; 462.1696 $^{13}C[M-H+HCOOH]^-$; 434.2019 $[M+NH_4]^+$; 435.2054 $^{13}C[M+NH_4]^+$	IV

C	Compound	Formula	RT	Ions	LI
C228	Unknown 017 (I)	$C_{26}H_{34}O_{12}$	288	583.2032 [M-H+HCOOH]$^-$	IV
C229	Unknown 017 (II)	$C_{26}H_{34}O_{12}$	407	539.2126 [M+H]$^+$	IV
C230	Unknown 018 (I)	$C_{24}H_{42}O_{11}$	338	551.2709 [M-H+HCOOH]$^-$	IV
C231	Unknown 018 (II)	$C_{24}H_{42}O_{11}$	344	551.2709 [M-H+HCOOH]$^-$; 524.3065 [M+NH$_4$]$^+$	IV
C232	Unknown 019 (I)	$C_{19}H_{24}O_7$	356	365.1596 [M+H]$^+$	IV
C233	Unknown 019 (II)	$C_{19}H_{24}O_7$	374	365.1597 [M+H]$^+$	IV
C234	Unknown 020 (I)	$C_{11}H_{16}O_3$	360	197.1172 [M+H]$^+$	IV
C235	Unknown 020 (II)	$C_{11}H_{16}O_3$	370	197.1173 [M+H]$^+$	IV
C236	Unknown 021 (I)	$C_{28}H_{36}O_{13}$	360	579.2083 [M-H]$^-$; 293.103 [frag]$^-$	IV
C237	Unknown 021 (II)	$C_{28}H_{36}O_{13}$	372	579.2083 [M-H]$^-$	IV
C238	Unknown 022 (I)	$C_{17}H_{22}O_5$	361	307.1542 [M+H]$^+$	IV
C239	Unknown 022 (II)	$C_{17}H_{22}O_5$	497	307.1542 [M+H]$^+$	IV
C240	Unknown 023 (I)	$C_{19}H_{24}O_6$	411	349.165 [M+H]$^+$	IV
C241	Unknown 023 (II)	$C_{19}H_{24}O_6$	539	349.1647 [M+H]$^+$; 417.1885 [+]	IV
C242	Unknown 024 (I)	$C_{26}H_{30}O_9$	455	485.1817 [M-H]$^-$	IV
C243	Unknown 024 (II)	$C_{26}H_{30}O_9$	461	485.1819 [M-H]$^-$	IV
C244	Unknown 025 (I)	$C_{12}H_{20}O_4$	486	227.1288 [M-H]$^-$	IV
C245	Unknown 025 (II)	$C_{12}H_{20}O_4$	548	227.1289 [M-H]$^-$	IV
C246	Unknown 026 (I)	$C_{23}H_{46}O_{16}$	616	577.2687 [M-H]$^-$	IV
C247	Unknown 026 (II)	$C_{23}H_{46}O_{16}$	623	577.2688 [M-H]$^-$; 578.272 ^{13}C[M-H]$^-$; 596.3098 [M+NH$_4$]$^+$	IV
C248	Unknown 027	273.9661	46	272.9589 [M-H]$^-$; 158.9786 [frag]$^-$; 288.9363 [-]	V
C249	Unknown 028	101.0265	47	102.0338 [M+H]$^+$; 81.5204 [frag]$^+$; 90.5258 [frag]$^+$; 84.9596 [+]; 86.9926 [+]; 94.045 [+]; 102.9702 [+]; 105.0032 [+]; 110.0086 [+]; 112.0062 [+]; 125.9862 [+]; 128.0192 [+]; 151.0353 [+]; 152.0364 [+]; 153.0329 [+]; 156.0142 [+]; 167.0129 [+]; 171.9918 [+]; 214.9179 [+]; 216.951 [+]; 223.9893 [+]; 239.9668 [+]; 255.9446 [+]; 265.016 [+]	V
C250	Unknown 029	297.8896	50	296.8823 [M-H]$^-$; 128.9598 [frag]$^-$; 212.9211 [frag]$^-$	V
C251	Unknown 030	291.8383	50	292.8456 [M+H]$^+$; 122.9244 frag[+]; 124.9225 frag[+]; 206.886 frag[+]; 290.8475 [+]	V
C252	Unknown 031	103.0996	52	104.1069 [M+H]$^+$	V
C253	Unknown 032	289.0688	52	290.0761 [M+H]$^+$	V
C254	Unknown 033	$C_{10}H_{18}N_2O_8$	53	295.1142 [M+H]$^+$; 252.1082 [+]; 266.1237 [+]	IV

C	Compound	Formula	RT	Ions	LI
C255	Unknown 034	$C_5H_6O_2$	54	116.0705 $[M+NH_4]^+$	IV
C256	Unknown 035	$C_{11}H_{12}O_3$	54	191.073 $[M-H]^-$	IV
C257	Unknown 036	$C_8H_4N_6O_4$	54	231.0267 $[M+H-H_2O]^+$	IV
C258	Unknown 037	$C_{12}H_{14}O_{10}$	55	317.0552 $[M-H]^-$; 125.0013 [-]	IV
C259	Unknown 038	H_3PO_4	55	96.9699 $[M-H]^-$; 98.9840 $[M+H]^+$	IV
C260	Unknown 039	$C_{12}H_{20}O_{12}$	56	355.0884 $[M-H]^-$	IV
C261	Unknown 040	$C_5H_8O_2$	56	118.0862 $[M+NH_4]^+$	IV
C262	Unknown 041	$C_{33}H_{12}O_2$	58	439.0774 $[M-H]^-$	IV
C263	Unknown 042	$C_{15}H_{22}O_5$	62	305.1346 [M+Na]+; 305.1346 $[M+Na]^+$	IV
C264	Unknown 043	$C_{20}H_{30}O_{14}$	74	512.1974 $[M+NH_4]^+$	IV
C265	Unknown 044	$C_{14}H_{20}O_9$	75	350.1449 $[M+NH_4]^+$	IV
C266	Unknown 045	$C_{14}H_{22}O_9$	76	333.1191 $[M-H]^-$; 352.1605 $[M+NH_4]^+$	IV
C267	Unknown 046	$C_5H_8O_5$	76	147.0300 $[M-H]^-$	IV
C268	Unknown 047	$C_4H_4O_4$	77	115.0039 $[M-H]^-$	IV
C269	Unknown 048	$C_{12}H_{14}O_3$	82	224.1283 $[M+NH_4]^+$	IV
C270	Unknown 049	$C_{14}H_{22}O_8$	84	363.1295 $[M-H+HCOOH]^-$; 319.1391 $[M+H]^+$	IV
C271	Unknown 050	$C_9H_{14}O_6$	85	217.0717 $[M-H]^-$	IV
C272	Unknown 051	$C_{14}H_{24}O_8$	88	365.1454 $[M-H+HCOOH]^-$; 321.1547 $[M+H]^+$; 338.1813 $[M+NH_4]^+$	IV
C273	Unknown 052	$C_{21}H_{25}NO_8$	91	420.1653 $[M+H]^+$	IV
C274	Unknown 053	$C_{11}H_{20}O_9$	94	295.1033 $[M-H]^-$; 314.1449 $[M+NH_4]^+$	IV
C275	Unknown 054	$C_{21}H_{38}O_{15}$	95	529.2139 $[M-H]^-$	IV
C276	Unknown 055	$C_{13}H_{14}O_4$	109	252.1231 $[M+NH_4]^+$	IV
C277	Unknown 056	$C_{12}H_{16}O_8$	110	333.0825 $[M-H+HCOOH]^-$; 306.1186 $[M+NH_4]^+$	IV
C278	Unknown 057	$C_{11}H_{20}O_4$	112	234.1702 $[M+NH_4]^+$	IV
C279	Unknown 058	$C_9H_{18}O_3$	128	175.1329 $[M+H]^+$	IV
C280	Unknown 059	$C_{14}H_{17}NO_7$	132	310.0931 $[M-H]^-$; 312.108 $[M+H]^+$	IV
C281	Unknown 060	$C_{11}H_{16}O_8$	135	275.0771 $[M-H]^-$	IV
C282	Unknown 061	$C_6H_{10}O_5$	152	161.0455 $[M-H]^-$	IV
C283	Unknown 062	$C_6H_{12}O_4$	154	147.0664 $[M-H]^-$	IV

C	Compound	Formula	RT	Ions	LI
C284	Unknown 063	$C_{14}H_{19}NO_5$	166	282.1338 [M+H]$^+$	IV
C285	Unknown 064	$C_{15}H_{26}O_{11}$	168	381.1404 [M-H]$^-$	IV
C286	Unknown 065	$C_{15}H_{19}NO_7$	170	326.1238 [M+H]$^+$	IV
C287	Unknown 066	$C_8H_6O_3$	172	149.0251 [M-H]$^-$	IV
C288	Unknown 067	$C_{16}H_{24}O_{10}$	197	375.1294 [M-H]$^-$	IV
C289	Unknown 068	$C_{32}H_{52}O_{20}$	207	755.2981 [M-H]$^-$; 447.1145 [-]	IV
C290	Unknown 069	$C_7H_{12}O_3$	209	145.0859 [M+H]$^+$	IV
C291	Unknown 070	$C_9H_{10}O_4$	213	181.0506 [M-H]$^-$; 200.0918 [M+NH$_4$]$^+$	IV
C292	Unknown 071	$C_{15}H_{22}O_8$	218	331.1391 [M+H]$^+$; 348.1656 [M+NH$_4$]$^+$	IV
C293	Unknown 072	C_6H_8O	225	114.0912 [M+NH$_4$]$^+$	IV
C294	Unknown 073	$C_{10}H_{10}O_3$	234	196.0969 [M+NH$_4$]$^+$	IV
C295	Unknown 074	$C_{14}H_{18}O_3$	238	252.1595 [M+NH$_4$]$^+$	IV
C296	Unknown 075	$C_7H_{12}O_5$	241	175.0613 [M-H]$^-$	IV
C297	Unknown 076	$C_{10}H_{14}O_6$	241	229.0719 [M-H]$^-$	IV
C298	Unknown 077	$C_{19}H_{28}O_{11}$	243	431.156 [M-H]$^-$; 477.1613 [M-H+HCOOH]$^-$; 450.1969 [M+NH$_4$]$^+$	IV
C299	Unknown 078	$C_{15}H_{22}O_8$	246	375.1296 [M-H+HCOOH]$^-$; 348.1656 [M+NH$_4$]$^+$	IV
C300	Unknown 079	$C_{14}H_{16}O_4$	247	266.1388 [M+NH$_4$]$^+$	IV
C301	Unknown 080	C_8H_{16}	248	130.1589 [M+NH$_4$]$^+$	IV
C302	Unknown 081	$C_{15}H_{18}O_8$	255	344.1341 [M+NH$_4$]$^+$	IV
C303	Unknown 082	$C_{17}H_{24}O_9$	259	390.176 [M+NH$_4$]$^+$; 193.086 [+]	IV
C304	Unknown 083	$C_{24}H_{36}O_{18}$	271	611.1826 [M-H]$^-$	IV
C305	Unknown 084	$C_9H_{12}O_2$	271	153.0909 [M+H]$^+$	IV
C306	Unknown 085	$C_{18}H_{20}O_4$	275	318.1701 [M+NH$_4$]$^+$	IV
C307	Unknown 086	$C_{18}H_{26}O_{10}$	275	447.1508 [M-H+HCOOH]$^-$; 420.1865 [M+NH$_4$]$^+$; 421.19 ^{13}C[M+NH$_4$]$^+$	IV
C308	Unknown 087	$C_8H_{18}O_{12}$	275	305.0699 [M-H]$^-$	IV
C309	Unknown 088	$C_{22}H_{32}O_{14}$	276	519.1718 [M-H]$^-$	IV
C310	Unknown 089	$C_{10}H_{16}O_3$	285	185.1172 [M+H]$^+$	IV
C311	Unknown 090	$C_{17}H_{26}O_{11}$	310	424.1812 [M+NH$_4$]$^+$	IV
C312	Unknown 091	$C_{14}H_{20}O_6$	312	302.1599 [M+NH$_4$]$^+$; 422.1657 [+]	IV

C	Compound	Formula	RT	Ions	LI
C313	Unknown 092	$C_{19}H_{30}O_9$	313	401.1817 [M-H]$^-$; 420.2229 [M+NH$_4$]$^+$	IV
C314	Unknown 093	$C_{17}H_{30}O_{10}$	313	412.2178 [M+NH$_4$]$^+$	IV
C315	Unknown 094	$C_{18}H_{32}O_{12}$	314	439.1822 [M-H]$^-$	IV
C316	Unknown 095	$C_{19}H_{32}O_{11}$	314	454.2281 [M+NH$_4$]$^+$	IV
C317	Unknown 096	$C_{10}H_{14}O_3$	315	183.1016 [M+H]$^+$	IV
C318	Unknown 097	$C_{26}H_{32}O_{13}$	319	551.1772 [M-H]$^-$	IV
C319	Unknown 098	$C_{23}H_{24}O_8$	319	446.1808 [M+NH$_4$]$^+$	IV
C320	Unknown 099	$C_{22}H_{40}O_{13}$	321	557.2453 [M-H+HCOOH]$^-$; 513.2541 [M+H]$^+$	IV
C321	Unknown 100	$C_9H_{14}O_2$	325	155.1066 [M+H]$^+$	IV
C322	Unknown 101	$C_{12}H_{14}O_6$	328	253.0719 [M-H]$^-$	IV
C323	Unknown 102	$C_{12}H_{16}O_5$	331	241.1072 [M+H]$^+$	IV
C324	Unknown 103	$C_{21}H_{30}O_{10}$	337	197.082 [dialdehydic elenolic ester decarboxymethyl - H]$^-$	IV
C325	Unknown 104	$C_{33}H_{44}O_{21}$	340	775.2301 [M-H]$^-$	IV
C326	Unknown 105	$C_{23}H_{24}O_{11}$	344	477.1388 [M+H]$^+$	IV
C327	Unknown 106	$C_{43}H_{30}O_5$	345	625.2036 [M-H]$^-$	IV
C328	Unknown 107	$C_{25}H_{36}O_{13}$	346	543.2081 [M-H]$^-$; 562.2494 [M+NH$_4$]$^+$	IV
C329	Unknown 108	$C_{27}H_{34}O_{11}$	347	533.2028 [M-H]$^-$	IV
C330	Unknown 109	$C_{17}H_{18}O_6$	347	317.103 [M-H]$^-$	IV
C331	Unknown 110	$C_{20}H_{16}O_5$	353	354.1335 [M+NH$_4$]$^+$	IV
C332	Unknown 111	$C_{22}H_{24}O_7$	355	401.1595 [M+H]$^+$; 598.2492 [+]	IV
C333	Unknown 112	$C_{16}H_{18}O_5$	362	291.1229 [M+H]$^+$	IV
C334	Unknown 113	$C_{23}H_{26}O_{10}$	364	461.1453 [M-H]$^-$	IV
C335	Unknown 114	$C_{28}H_{34}O_{13}$	365	623.1982 [M-H+HCOOH]$^-$; 596.2333 [M+NH$_4$]$^+$	IV
C336	Unknown 115	$C_{36}H_{46}O_{19}$	372	781.2561 [M-H]$^-$	IV
C337	Unknown 116	$C_{24}H_{28}O_{11}$	372	491.156 [M-H]$^-$; 493.1701 [M+H]$^+$; 339.1076 [+]	IV
C338	Unknown 117	$C_{34}H_{46}O_{21}$	373	789.2457 [M-H]$^-$	IV
C339	Unknown 118	$C_{30}H_{40}O_{14}$	374	623.2346 [M-H]$^-$; 381.1556 [frag]$^-$	IV
C340	Unknown 119	$C_{23}H_{30}O_{10}$	381	484.2177 [M+NH$_4$]$^+$	IV
C341	Unknown 120	$C_{39}H_{26}O_3$	381	541.1822 [M-H]$^-$; 560.2239 [M+NH$_4$]$^+$	IV

C	Compound	Formula	RT	Ions	LI
C342	Unknown 121	$C_{27}H_{22}O_{12}$	388	539.118 $[M+H]^+$	IV
C343	Unknown 122	$C_{34}H_{40}O_{13}$	388	655.2401 $[M-H]^-$	IV
C344	Unknown 123	$C_{36}H_{30}O_{16}$	388	717.1461 $[M-H]^-$; 718.1492 $^{13}C[M-H]^-$; 719.1518 (2)$^{13}C[M-H]^-$; 736.1864 $[M+NH_4]^+$; 737.19 $^{13}C[M+NH_4]^+$	IV
C345	Unknown 124	$C_{46}H_{39}NO_6$	390	700.269 $[M-H]^-$	IV
C346	Unknown 125	$C_{36}H_{40}O_{15}$	397	713.2446 $[M+H]^+$	IV
C347	Unknown 126	$C_{15}H_{24}O$	397	221.1901 $[M+H]^+$; 441.2458 [+]	IV
C348	Unknown 127	$C_{21}H_{38}O_8$	397	463.2549 $[M-H+HCOOH]^-$; 419.264 $[M+H]^+$	IV
C349	Unknown 128	$C_{38}H_{50}O_{18}$	402	793.2923 $[M-H]^-$	IV
C350	Unknown 129	$C_{27}H_{38}O_{15}$	405	601.2134 $[M-H]^-$; 602.2168 $^{13}C[M-H]^-$	IV
C351	Unknown 140	$C_{38}H_{46}O_{18}$	409	789.2613 $[M-H]^-$	IV
C352	Unknown 141	$C_{20}H_{26}O_9$	409	428.1918 $[M+NH_4]^+$; 456.2227 [+]	IV
C353	Unknown 142	$C_{17}H_{20}O_4$	424	289.1436 $[M+H]^+$	IV
C354	Unknown 143	$C_{20}H_{23}NO_7$	430	390.1548 $[M+H]^+$	IV
C355	Unknown 144	$C_{25}H_{30}O_8$	442	321.1341 $[M-H-H_2O-C_8H_6O]^-$	IV
C356	Unknown 145	$C_{19}H_{24}O_9$	444	397.1495 $[M+H]^+$	IV
C357	Unknown 146	$C_{19}H_{22}O_9$	450	393.1192 $[M-H]^-$	IV
C358	Unknown 147	$C_{11}H_{16}O_4$	450	213.1122 $[M+H]^+$	IV
C359	Unknown 148	$C_{19}H_{26}O_8$	463	349.1294 $[M-H-CH_3OH]^-$	IV
C360	Unknown 149	$C_{26}H_{44}O_{11}$	470	577.2867 $[M-H+HCOOH]^-$; 533.2953 $[M+H]^+$	IV
C361	Unknown 150	$C_{25}H_{28}O_9$	472	471.166 $[M-H]^-$; 490.2068 $[M+NH_4]^+$	IV
C362	Unknown 151	$C_{31}H_{26}O_7$	483	319.1187 $[DOA-H]^-$	IV
C363	Unknown 152	$C_{18}H_{36}O_5$	485	331.2492 $[M-H]^-$; 333.2638 $[M+H]^+$	IV
C364	Unknown 153	$C_{33}H_{38}O_{11}$	495	609.234 $[M-H]^-$	IV
C365	Unknown 154	358.1393	499	359.1466 $[M+H]^+$	V
C366	Unknown 155	$C_{20}H_{28}O_7$	508	379.1763 $[M-H]^-$	IV
C367	Unknown 156	$C_{16}H_{32}O_4$	509	287.2226 $[M-H]^-$; 289.2374 $[M+H]^+$	IV
C368	Unknown 157	$C_{13}H_{18}O_6$	516	271.1178 $[M+H]^+$; 239.0915 $[M+H-CH_3OH]^+$	IV
C369	Unknown 158	$C_{18}H_{22}O_5$	520	319.1543 $[M+H]^+$	IV
C370	Unknown 159	$C_{11}H_{16}O_2$	521	181.1223 $[M+H]^+$	IV

C	Compound	Formula	RT	Ions	LI
C371	Unknown 160	$C_{19}H_{20}O_8$	521	377.1234 [M+H]$^+$; 440.1917 [+]; 445.1469 [+]	IV
C372	Unknown 161	$C_{20}H_{26}O_5$	529	345.1708 [M-H]$^-$	IV
C373	Unknown 162	$C_{27}H_{34}O_8$	546	485.2181 [M-H]$^-$	IV
C374	Unknown 163	$C_{34}H_{58}O_{16}$	575	721.3654 [M-H]$^-$	IV
C375	Unknown 164	$C_{20}H_{40}O_2$	588	330.3368 [M+NH$_4$]$^+$	IV
C376	Unknown 165	$C_{22}H_{42}O_2$	594	356.3524 [M+NH$_4$]$^+$	IV
C377	Unknown 166	$C_{28}H_{47}N_3O_6$	622	566.3462 [M-H+HCOOH]$^-$; 522.3554 [M+H]$^+$	IV
C378	Unknown 167	$C_{28}H_{44}O_{11}$	709	555.2842 [M-H]$^-$; 556.2875 ^{13}C[M-H]$^-$; 574.3257 [M+NH$_4$]$^+$	IV

Table S2. MS/MS fragments of detected compounds by LTQ-Orbitrap.

C	Compound	MS^2 and pseudo-MS^n spectra
C001	Gluconic acid	195.0511 $[M-H]^-$: 129.0195 (100), 177.0405 (12)
C002	Quinic acid	191.0564 $[M-H]^-$: 85.0299 (38), 93.0350 (24), 109.0299 (11), 111.0456 (14), 127.0405 (31), 173.0459 (19), 191.0564 (100)
C003	Malic acid	133.0146 $[M-H]^-$: 115.0040 (100)
C004	Isocitric acid	191.0200 $[M-H]^-$: 111.0090 (100), 173.0093 (10)
C005	Citric acid	191.0199 $[M-H]^-$: 111.0090 (100), 173.0093 (11)
C006	Succinic acid	117.0197 [M-H]-: 73.0299 (76), 99.0091 (25), 117.0196 (100)
C007	Sugar alcohol	181.072 $[M-H]^-$: 59.0143 (22), 71.0143 (23), 85.0299 (10), 89.0248 (36), 101.0248 (100), 113.0248 (23), 119.0353 (13), 131.0353 (16), 143.0352 (10), 163.0614 (78), 181.0721 (42)<br>183.0865 $[M+H]^+$: 69.0333 (19), 129.0544 (34), 147.0651 (82), 165.0755 (100)
C008	Pentose acid	165.041 $[M-H]^-$: 75.0091 (13), 87.0091 (10), 99.0091 (16), 101.0247 (23), 105.0196 (76), 129.0196 (45), 147.0301 (100)
C009	Hexose	179.0565 $[M-H]^-$: 75.0090 (16), 81.0349 (14), 99.0091 (20), 143.0351 (100), 161.0457 (44)<br>225.062 $[M-H+HCOOH]^-$: 89.0247 (19), 161.0458 (11), 179.0562 (100)
C010	Di-hexose	341.1094 $[M-H]^-$: 101.0246 (24), 161.0457 (100), 179.0565 (25)<br>387.1148 $[M-H+HCOOH]^-$: 161.0457 (43), 179.0562 (14), 341.1088 (100)<br>360.1505 $[M+NH_4]^+$: 163.0600 (13), 325.1128 (100)
C011	Tri-hexose	549.1677 $[M-H+HCOOH]^-$: 161.0457 (12), 341.1087 (40), 443.1406 (10), 503.1619 (100)<br>522.2028 $[M+NH_4]^+$: 325.1129 (100), 487.1655 (67), 505.1773 (15)
C012	Tetra-hexose	711.2203 $[M-H+HCOOH]^-$: 665.2143 (100)
C013	Glycerol	93.0500 $[M+H]^+$: 57.0333 (78), 75.0439 (100)
C014	Trihydroxy-octadecadienoic acid	327.2179 $[M-H]^-$: 171.1028 (100), 201.1132 (12), 211.1340 (27), 221.1183 (11), 229.1447 (51), 239.1289 (11), 291.1966 (44), 309.2072 (16)
C015	Trihydroxyoctadecenoic acid (I)	329.2338 $[M-H]^-$: 171.1028 (28), 201.1132 (100), 293.2122 (23), 311.2225 (46)<br>331.2481 $[M+H]^+$: 277.2164 (26), 295.2267 (100), 313.2375 (53)
C016	Trihydroxyoctadecenoic acid (II)	329.2337 $[M-H]^-$: 171.1028 (100), 211.1340 (70), 229.1446 (92), 293.2122 (31), 311.2228 (38)
C017	Apigenin glucoside (I)	431.0991 $[M-H]^-$: 311.0560 (100), 341.0668 (28)<br>433.1129 $[M+H]^+$: 313.0708 (22), 337.0707 (15), 367.0814 (38), 379.0811 (11), 397.0917 (31), 415.1020 (100)

C	Compound	MS² and pseudo-MSⁿ spectra
C018	Apigenin glucoside (II)	431.0991 [M-H]⁻: 268.0378 (12), 269.0456 (100)<br>433.113 [M+H]⁺: 271.0601 (100)
C019	Apigenin rutinoside (I)	577.1566 [M-H]⁻: 293.0455 (28), 413.0877 (100)<br>579.1716 [M+H]⁺: 271.0601 (4), 415.1022 (18), 433.1126 (100)
C020	Apigenin rutinoside (II)	577.1566 [M-H]⁻: 269.0455 (100)<br>579.1714 [M+H]⁺: 271.0601 (18), 433.1130 (100)
C021	Apigenin rhamnosyl acetyl-glucoside (I)	621.1814 [M+H]⁺: 271.0604 (8), 313.0710 (11), 415.1023 (15), 433.1128 (100)
C022	Apigenin rhamnosyl acetyl-glucoside (II)	619.1674 [M-H]⁻: 293.0451 (32), 413.0876 (100), 455.0993 (14), 559.1456 (28), 577.1561 (11)<br>621.1816 [M+H]⁺: 271.0599 (5), 313.0709 (14), 415.1023 (17), 433.1127 (100)
C023	Apigenin rhamnosyl acetyl-glucoside (III)	619.1672 [M-H]⁻: 293.0456 (36), 413.0879 (100)<br>621.1815 [M+H]⁺: 271.0602 (6), 415.1023 (14), 433.1128 (100)
C024	Apiin	563.1413 [M-H]⁻: 353.0666 (43), 383.0773 (32), 443.0985 (100), 473.1090 (75), 503.1194 (20), 545.1298 (16)
C025	Methoxy-apigenin glucoside	461.1096 [M-H]⁻: 284.0323 (11), 298.0481 (14), 299.0560 (100), 446.0853 (74)<br>463.124 [M+H]⁺: 301.0705 (100)
C026	Luteolin	285.0405 [M-H]⁻: 133.0297 (16), 149.0246 (10), 151.0037 (47), 175.0401 (100), 197.0608 (21), 198.0321 (11), 199.0400 (86), 201.0194 (23), 213.0558 (24), 217.0505 (66), 223.0401 (14), 241.0507 (95), 243.0298 (56), 257.0457 (18), 267.0292 (12), 285.0416 (2)<br>287.0553 [M+H]⁺: 153.0182 (45), 177.0675 (100), 287.0560 (4)
C027	Luteolin glucoside (I)	447.0936 [M-H]⁻: 285.0405 (100)<br>449.1085 [M+H]⁺: 287.0550 (100)<br>287.055 [M+H-hexose]⁺: 135.0439 (13), 153.0181 (95), 161.0229 (10), 177.0674 (100), 287.0561 (4)
C028	Luteolin glucoside (II)	447.0934 [M-H]⁻: 285.0404 (100)<br>449.1082 [M+H]⁺: 287.0551 (100)
C029	Luteolin rutinoside	593.1517 [M-H]⁻: 285.0402 (100)<br>595.1654 [M+H]⁺: 287.0545 (12), 449.1082 (100)
C030	Quercetin glucoside	463.0881 [M-H]⁻: 300.0277 (33), 301.0353 (100)<br>465.1025 [M+H]⁺: 303.0500 (100)<br>303.05 [M+H-hexose]⁺: 137.0229 (19), 153.0187 (20), 165.0182 (63), 229.0495 (92), 247.0602 (30), 257.0443 (100), 285.0388 (51)
C031	Quercetin rutinoside	609.1467 [M-H]⁻: 300.0276 (34), 301.0353 (100)<br>611.1611 [M+H]⁺: 303.0501 (100), 465.1025 (30)

C	Compound	MS^2 and pseudo-MS^n spectra
C032	Caffeic acid hexoside	341.0882 $[M-H]^-$: 135.0454 (11), 179.0351 (100), 251.0559 (14), 281.0668 (20)
C033	Caffeic acid rutinoside	487.1464 $[M-H]^-$: 179.0350 (100)
C034	Caffeic acid ethyl ester	207.0664 $[M-H]^-$: 135.0452 (23), 161.0246 (18), 179.0350 (100)
C035	Caffeoyl-threonic acid (I)	297.0616 $[M-H]^-$: 135.0301 (100), 135.0457 (2), 179.0350 (19)
C036	Caffeoyl-threonic acid (II)	297.0617 $[M-H]^-$: 135.0301 (100), 135.0458 (2), 179.0351 (21)
C037	Calceolarioside B	477.1411 $[M-H]^-$: 161.0246 (100), 315.1085 (19)
		479.155 $[M+H]^+$: 163.0387 (28), 263.0916 (19), 299.1123 (15), 325.0920 (41), 443.1335 (33), 461.1445 (100)
C038	Rosmarinic acid	359.0777 $[M-H]^-$: 161.0246 (100), 179.0350 (23), 197.0456 (25)
C039	Coumaroylquinic acid	337.0931 $[M-H]^-$: 191.0560 (100)
		339.1077 $[M+H]^+$: 147.0439 (100)
C040	Cinnamic acid hexoside	309.0984 $[M-H]^-$: 111.0454 (47), 113.0247 (11), 129.0559 (12), 173.0457 (41), 223.0613 (27), 247.0976 (100), 291.0872 (43)
C041	Neochlorogenic acid	353.088 $[M-H]^-$: 179.0351 (49), 191.0562 (100)
		355.1028 $[M+H]^+$: 163.0388 (100)
C042	Chlorogenic acid	353.088 $[M-H]^-$: 191.0562 (100)
		191.0562 $[quinic-H]^-$: 85.0297 (100), 87.0090 (17), 93.0348 (61), 109.0296 (29), 111.0453 (31), 127.0402 (84), 171.0299 (21), 173.0456 (53)
		355.1031 $[M+H]^+$: 163.0389 (100)
C043	Verbascoside	623.1985 $[M-H]^-$: 315.1083 (2), 461.1662 (100)
C044	Isoverbascoside	623.1991 $[M-H]^-$: 315.1085 (2), 461.1662 (100)
C045	Hydroxy-verbascoside	639.1937 $[M-H]^-$: 459.1509 (10), 487.1463 (3), 529.1565 (14), 621.1830 (100)
C046	Methyl-hydroxy-verbascoside (I)	653.2092 $[M-H]^-$: 459.1509 (3), 621.1825 (100)
C048	Dimethyl-hydroxy-verbascoside	667.225 $[M-H]^-$: 459.1504 (5), 621.1823 (100)
C049	3,4-Dihydroxyphenylglycol	169.051 $[M-H]^-$: 151.0402 (100)
		151.0405 $[M-H-H_2O]^-$: 123.0454 (100)
		171.0652 $[M+H]^+$: 139.0387 (100)
C050	Hydroxytyrosol	153.0562 $[M-H]^-$: 123.0455 (100), 153.0560 (23)
C051	Hydroxytyrosol glucoside (I)	153.056 $[M-H-hexose]^-$: 85.0299 (11), 89.0246 (69), 101.0246 (69), 113.0246 (100), 135.0452 (21), 153.0558 (41)
		317.1237 $[M+H]^+$: 137.0595 (100), 221.0799 (13), 299.1125 (30)

C	Compound	MS^2 and pseudo-MS^n spectra
C052	Hydroxytyrosol glucoside (II)	315.1085 [M-H]$^-$: 123.0455 (10), 135.0453 (34), 153.0559 (100)<br>334.1499 [M+NH_4]$^+$: 137.0596 (79), 155.0702 (10), 155.0718 (1), 251.0914 (10), 263.0916 (46), 281.1020 (35), 299.1128 (22), 317.1232 (100)
C053	Hydroxytyrosol rutinoside	461.1666 [M-H]$^-$: 135.0453 (48), 297.0981 (15), 315.1085 (100)<br>153.0562 [M-H-rutinoside]$^-$: 123.0454 (100)<br>480.2082 [M+NH_4]$^+$: 247.0969 (13), 265.1072 (20), 273.0971 (13), 301.1287 (37), 309.1181 (49), 463.1811 (100)
C054	Dimer of hydroxytyrosol (I)	305.1034 [M-H]$^-$: 123.0454 (2), 135.0455 (1), 153.0560 (10), 245.0823 (2), 275.0925 (100), 287.0925 (6)
C055	Dimer of hydroxytyrosol (II)	305.1032 [M-H]$^-$: 245.0820 (21), 275.0923 (100), 287.0924 (7)
C056	Dimer of hydroxytyrosol (III)	305.1033 [M-H]$^-$: 245.0821 (7), 275.0925 (100), 287.0925 (52)
C057	Dimer of hydroxytyrosol (IV)	305.1032 [M-H]$^-$: 123.0454 (16), 151.0403 (41), 153.0559 (100)<br>324.1446 [M+NH_4]$^+$: 137.0595 (52), 153.0546 (100), 155.0701 (19), 271.0962 (20), 289.1062 (40), 307.1181 (16)
C059	Lactone (ester with hydroxytyrosol) (I)	321.1348 [M-H]$^-$: 185.0819 (100)
C060	Lactone (ester with hydroxytyrosol) (II)	321.1346 [M-H]$^-$: 111.0454 (14), 111.0818 (34), 185.0819 (100), 303.1238 (13)<br>323.1493 [M+H]$^+$: 201.0918 (15), 219.1025 (26), 261.1124 (100), 279.1229 (78)
C061	Lactone (ester with hydroxytyrosol) (III)	321.1348 [M-H]$^-$: 185.0820 (100)<br>323.1498 [M+H]$^+$: 137.0603 (31), 153.0905 (22), 169.0866 (100)
C062	Lactone (ester with hydroxytyrosol) (IV)	321.1346 [M-H]$^-$: 185.0819 (100)<br>323.1493 [M+H]$^+$: 137.0594 (100)
C063	Lactone glucoside (ester with hydroxytyrosol) (I)	483.1877 [M-H]$^-$: 211.1130 (30), 241.1236 (25), 285.1133 (100), 303.1242 (10), 465.1769 (84)<br>485.2014 [M+H]$^+$: 269.1170 (57), 287.1276 (100), 305.1383 (24), 323.1485 (16)
C064	Lactone glucoside (ester with hydroxytyrosol) (II)	483.1876 [M-H]$^-$: 347.1348 (100)
C065	Tyrosol glucoside	299.114 [M-H]$^-$: 89.0247 (66), 101.0247 (70), 113.0247 (100), 119.0353 (42), 119.0505 (20), 125.0247 (11), 131.0353 (23), 143.0351 (81), 161.0457 (33), 179.0563 (64)<br>318.155 [M+NH_4]$^+$: 121.0646 (11), 187.0754 (66), 205.0858 (74), 229.0860 (49), 247.0965 (80), 265.1070 (100), 301.1285 (42)
C066	Homogentisic acid	167.0352 [M-H]$^-$: 123.0454 (100)
C068	Oleuropein isomer (I)	539.1772 [M-H]$^-$: 179.0561 (17), 179.0723 (4), 223.0613 (100), 333.0825 (15), 377.1234 (6), 403.1247 (54)
C069	Oleuropein	539.1773 [M-H]$^-$: 275.0571 (16), 275.0927 (72), 307.0826 (90), 345.0988 (15), 377.1249 (100)<br>377.1245 [M-H-hexose]$^-$: 275.0923 (86), 307.0821 (100), 345.0982 (19)

C	Compound	MS² and pseudo-MSⁿ spectra
		558.2184 $[M+NH_4]^+$: 361.1282 (100), 379.1387 (24)
		361.1284 $[M+H\text{-hexose-}H_2O]^+$: 137.0595 (100), 165.0546 (17), 329.1018 (18)
C070	Oleuropein isomer (II)	539.1774 $[M-H]^-$: 275.0562 (14), 275.0924 (74), 307.0822 (91), 327.0876 (13), 345.0983 (19), 377.1243 (100)
C071	Oleuropein isomer (III)	539.1774 $[M-H]^-$: 275.0562 (23), 275.0923 (63), 307.0821 (100), 345.0982 (10), 377.1243 (45), 403.1248 (21)
		379.1388 $[M+H\text{-hexose}]^+$: 137.0595 (72), 165.0545 (15), 225.0758 (36), 243.0863 (12), 347.1125 (39), 361.1282 (100)
C072	Oleuropein isomer (IV)	539.1773 $[M-H]^-$: 275.0557 (9), 275.0922 (59), 307.0820 (72), 345.0985 (10), 377.1241 (100)
C073	Oleuropein glucoside (I)	701.2301 $[M-H]^-$: 315.1082 (100), 437.1441 (19), 469.1352 (46), 539.1766 (26)
C074	Oleuropein glucoside (II)	701.2306 $[M-H]^-$: 539.1768 (100)
		720.2707 $[M+NH_4]^+$: 347.1128 (11), 361.1285 (44), 379.1387 (100), 541.1918 (44)
C075	Oleuropein glucoside (III)	701.2299 $[M-H]^-$: 275.0559 (29), 275.0921 (75), 307.0819 (100), 327.0873 (11), 345.0978 (16), 377.1239 (85), 539.1776 (6), 565.1769 (46), 669.2029 (11)
C076	Oleuropein aglycone (I)	377.1246 $[M-H]^-$: 241.0718 (100), 275.0925 (2), 307.0819 (2), 345.0985 (1)
		424.1969 $[M+C_2H_8N]^+$: 137.0594 (15), 361.1281 (100)
		361.1283 $[M+H-H_2O]^+$: 137.0595 (100), 165.0547 (15), 329.1023 (15)
C077	Oleuropein aglycone (II)	377.1247 $[M-H]^-$: 275.0554 (2), 275.0923 (86), 307.0821 (100), 345.0981 (18)
		379.1389 $[M+H]^+$: 137.0595 (100), 225.0756 (71), 347.1124 (25), 361.1279 (30)
C078	Oleuropein aglycone (III)	377.1247 $[M-H]^-$: 275.0555 (2), 275.0924 (84), 307.0822 (100), 345.0983 (18)
		379.1389 $[M+H]^+$: 137.0595 (100), 225.0760 (25), 347.1124 (24), 361.1276 (20)
C079	Oleuropein aglycone (IV)	377.1248 $[M-H]^-$: 275.0554 (2), 275.0924 (86), 307.0822 (100), 345.0982 (18)
		379.1389 $[M+H]^+$: 137.0595 (100), 225.0758 (46), 243.0861 (12), 347.1123 (42)
C080	Hydroxy-oleuropein	555.1725 $[M-H]^-$: 223.0613 (16), 291.0512 (1), 291.0871 (12), 323.0771 (16), 393.1193 (31), 403.1248 (48), 537.1612 (100)
C081	Methyl-oleuropein aglycone (I)	391.1403 $[M-H]^-$: 151.0765 (100)
C082	Methyl-oleuropein aglycone (II)	391.1403 $[M-H]^-$: 223.0612 (7), 255.0875 (100)
C083	Methyl-oleuropein aglycone (III)	391.1401 $[M-H]^-$: 211.0976 (19), 223.0612 (4), 255.0874 (100), 359.1137 (10)
C084	Hydroxy-methyl-oleuropein	569.1882 $[M-H]^-$: 223.0613 (11), 403.1247 (75), 407.1348 (13), 537.1611 (100)
C085	Demethyloleuropein	525.162 $[M-H]^-$: 165.0558 (21), 195.0663 (100), 249.0767 (12), 301.1081 (23), 319.1187 (28), 363.1088 (14), 389.1089 (44), 481.1721 (62)
C086	DOA (I)	319.119 $[M-H]^-$: 123.0454 (8), 135.0454 (1), 153.0559 (49), 165.0558 (4), 257.1185 (19), 259.0976 (81), 275.1289 (100)
		321.1334 $[M+H]^+$: 137.0594 (2), 249.0755 (23), 285.1120 (14), 303.1227 (100)
C087	DOA (II)	321.1336 $[M+H]^+$: 137.0595 (100), 303.1230 (16)

C	Compound	MS^2 and pseudo-MS^n spectra
C088	DOA (III)	319.1193 [M-H]⁻: 123.0454 (10), 135.0454 (1), 153.0559 (51), 165.0558 (4), 257.1185 (16), 259.0977 (74), 275.1289 (100)<br>321.1334 [M+H]⁺: 137.0596 (6), 167.0699 (1), 249.0759 (13), 285.1124 (12), 303.1230 (100)
C089	DOA (IV)	319.1191 [M-H]⁻: 165.0558 (21), 195.0662 (100), 301.1081 (12)<br>321.1336 [M+H]⁺: 137.0594 (16), 261.1127 (13), 285.1132 (11), 303.1230 (100)
C090	DOA (V)	319.1191 [M-H]⁻: 139.0767 (21), 183.0664 (100)<br>321.1336 [M+H]⁺: 137.0595 (100), 185.0807 (4)
C091	DOA (VI)	319.1191 [M-H]⁻: 139.0767 (8), 183.0663 (100)<br>321.1335 [M+H]⁺: 137.0595 (100), 303.1228 (14)
C092	DOA linked to hydroxytyrosol (I)	455.1719 [M-H]⁻: 205.0872 (17), 257.1183 (35), 275.1294 (13), 301.1083 (25), 319.1188 (100), 385.1291 (33), 425.1603 (20), 437.1607 (51)
C093	DOA linked to hydroxytyrosol (II)	455.1715 [M-H]⁻: 153.0560 (24), 257.1191 (14), 259.0973 (17), 275.0926 (18), 275.1294 (19), 301.1073 (11), 319.1187 (85), 385.1291 (100), 437.1602 (20)<br>457.1864 [M+H]⁺: 137.0596 (17), 303.1231 (53), 321.1329 (10), 385.1284 (29), 439.1755 (100)
C094	DOA linked to hydroxytyrosol (III)	455.1714 [M-H]⁻: 153.0559 (25), 259.0975 (16), 275.0929 (18), 275.1282 (15), 319.1186 (77), 385.1292 (100), 437.1606 (13)<br>457.1869 [M+H]⁺: 137.0597 (20), 303.1233 (55), 385.1283 (32), 439.1756 (100)
C095	Hydroxy-DOA	335.114 [M-H]⁻: 199.0613 (100)<br>337.1284 [M+H]⁺: 137.0595 (32), 277.1063 (17), 301.1082 (13), 319.1176 (100)
C096	Hydrated-DOA (I)	337.1295 [M-H]⁻: 201.0769 (100)<br>339.144 [M+H]⁺: 137.0595 (100), 309.1331 (13)
C097	Hydrated-DOA (II)	337.1296 [M-H]⁻: 201.0769 (100)
C098	Hydrated-DOA (III)	337.1296 [M-H]⁻: 123.0453 (27), 153.0558 (100), 165.0557 (48), 183.0662 (19), 199.0616 (11), 319.1186 (48)
C099	Hydrated-DOA linked to hydroxytyrosol (I)	473.1823 [M-H]⁻: 153.0558 (26), 249.0768 (14), 257.1185 (14), 275.1292 (11), 319.1186 (69), 403.1402 (74), 413.1608 (19), 455.1713 (100)
C100	Hydrated-DOA linked to hydroxytyrosol (II)	473.1822 [M-H]⁻: 201.0768 (16), 337.1293 (100)<br>492.2228 [M+NH_4]⁺: 165.0546 (17), 321.1330 (100), 457.1866 (15)
C101	Hydrated-DOA linked to hydroxytyrosol (III)	473.1819 [M-H]⁻: 337.1293 (100)
C102	Hydrated-DOA linked to hydroxytyrosol glucoside	635.2351 [M-H]⁻: 195.0662 (83), 301.1079 (13), 319.1186 (100), 473.1815 (45), 545.2031 (18), 575.2122 (20)<br>654.2756 [M+NH_4]⁺: 303.1227 (30), 321.1336 (51), 457.1861 (40), 475.1960 (100), 619.2387 (19)
C103	Methyl-DOA	333.1346 [M-H]⁻: 153.0921 (16), 197.0819 (100)<br>335.1488 [M+H]⁺: 137.0595 (100), 190.9978 (57), 317.1375 (13)
C104	Acetal of DOA (I)	365.1607 [M-H]⁻: 183.0663 (44), 229.1081 (100), 319.1182 (46)

C	Compound	MS² and pseudo-MSⁿ spectra
C105	Acetal of DOA (II)	229.1084 [M-H-hydroxytyrosol]⁻: 121.0662 (37), 137.0610 (14), 153.0922 (100), 185.1183 (64), 201.1131 (11)
C106	Acetal of DOA (III)	365.1608 [M-H]⁻: 229.1082 (100)
C107	Acetal of DOA (IV)	365.161 [M-H]⁻: 195.0664 (30), 229.1083 (100), 319.1190 (11)<br>384.2021 [M+NH$_4$]⁺: 121.0656 (19), 133.1012 (22), 133.1233 (30), 195.1016 (56), 213.1124 (100)
C108	Acetal of DOA linked to hydroxytyrosol	501.2132 [M-H]⁻: 365.1605 (100)
C109	Ligstroside (I)	523.1829 [M-H]⁻: 259.0978 (35), 291.0873 (56), 361.1296 (100)<br>361.1299 [M-H-hexose]⁻: 259.0976 (75), 291.0871 (100)<br>542.2246 [M+NH$_4$]⁺: 225.0758 (29), 387.1285 (37), 507.1857 (100)
C110	Ligstroside (II)	523.1829 [M-H]⁻: 259.0975 (16), 291.0871 (26), 361.1292 (29), 421.1501 (37), 453.1401 (100)<br>525.1974 [M+H]⁺: 225.0751 (16), 347.1142 (10), 475.1619 (34), 507.1837 (100)
C111	Ligstroside glucoside	685.2349 [M-H]⁻: 299.1137 (13), 421.1501 (24), 453.1401 (65), 523.1822 (100)<br>704.2764 [M+NH$_4$]⁺: 369.1182 (37), 475.1596 (14), 489.1758 (99), 507.1858 (100)
C112	Ligstroside aglycone	361.1299 [M-H]⁻: 259.0976 (73), 291.0871 (100)<br>363.1443 [M+H]⁺: 121.0646 (100), 225.0762 (22), 331.1182 (17)
C113	Decarboxymethyl ligstroside aglycone	349.1295 [M-H+HCOOH]⁻: 139.0766 (11), 169.0871 (18), 213.0768 (100)<br>305.1389 [M+H]⁺: 137.0595 (19), 169.0859 (100)
C114	Hydroxy-decarboxymethyl-ligstroside aglycone	319.1189 [M-H]⁻: 111.0090 (40), 121.0298 (16), 153.0560 (10), 181.0507 (100), 199.0612 (16)<br>321.1335 [M+H]⁺: 121.0646 (100), 137.0595 (24), 151.0755 (15), 303.1229 (15)
C115	Oleoside	389.1097 [M-H]⁻: 121.0297 (12), 121.0662 (25), 165.0558 (73), 183.0663 (100), 209.0459 (14), 227.0562 (81), 345.1200 (4)<br>391.1238 [M+H]⁺: 211.0601 (60), 229.0707 (100)
C116	Secologanoside	389.1096 [M-H]⁻: 121.0662 (15), 165.0559 (20), 183.0664 (6), 209.0457 (32), 345.1193 (100)<br>408.1502 [M+NH$_4$]⁺: 211.0599 (40), 229.0706 (100)
C117	Oleoside aglycone	227.0564 [M-H]⁻: 139.0767 (100), 165.0558 (100), 183.0663 (51)<br>229.0709 [M+H]⁺: 193.0494 (13), 211.0600 (100)
C118	Caffeoyl-oleoside	551.1409 [M-H]⁻: 179.0351 (11), 251.0562 (16), 281.0668 (24), 341.0879 (30), 345.1193 (10), 389.1092 (24), 507.1508 (100), 533.1305 (18)<br>553.1561 [M+H]⁺: 163.0390 (85), 181.0498 (26), 211.0601 (11), 325.0922 (100), 535.1448 (88)
C119	Caffeoyl-oleoside glucoside	713.194 [M-H]⁻: 323.0771 (13), 345.1180 (2), 443.1196 (20), 489.1402 (28), 507.1507 (69), 533.1305 (67), 551.1406 (100), 669.2031 (12)

C	Compound	MS² and pseudo-MSⁿ spectra
C120	Coumaroyl-oleoside	535.1465 [M-H]⁻: 235.0615 (12), 265.0718 (20), 345.1193 (16), 389.1092 (22), 491.1559 (100)<br>537.1609 [M+H]⁺: 147.0439 (40), 165.0546 (26), 291.0859 (13), 309.0971 (100), 519.1494 (76)
C121	Dimethyl-hydroxy-octenoyloxy-secologanoside (I)	557.2247 [M-H]⁻: 165.0557 (30), 183.0662 (45), 185.1183 (68), 199.1341 (25), 209.0455 (18), 227.0562 (41), 227.1287 (37), 257.1393 (12), 371.0980 (100), 389.1086 (28)
C122	Dimethyl-hydroxy-octenoyloxy-secologanoside (II)	557.2251 [M-H]⁻: 185.1183 (13), 199.1340 (13), 227.1290 (16), 345.1192 (29), 513.2345 (100)<br>559.2401 [M+H]⁺: 211.0601 (23), 295.1547 (14), 331.1752 (60), 361.1643 (17), 379.1752 (14), 505.2069 (42), 523.2173 (76), 541.2285 (100)
C124	Elenolic acid (II)	241.0723 [M-H]⁻: 95.0506 (60), 101.0247 (29), 121.0299 (18), 127.0403 (82), 139.0402 (100), 165.0558 (29), 197.0822 (11), 209.0456 (14)<br>243.0867 [M+H]⁺: 165.0546 (22), 207.0652 (17), 211.0600 (20), 225.0758 (100)
C125	Elenolic acid (III)	241.0723 [M-H]⁻: 95.0505 (1), 127.0404 (1), 139.0403 (1), 165.0558 (100)<br>243.0866 [M+H]⁺: 193.0494 (11), 207.0651 (17), 211.0600 (22), 225.0758 (100)
C126	Elenolic acid (IV)	241.0721 [M-H]⁻: 95.0506 (58), 101.0247 (32), 121.0299 (21), 127.0403 (82), 139.0402 (100), 165.0558 (32), 209.0456 (15)<br>243.0865 [M+H]⁺: 139.0388 (14), 165.0545 (16), 211.0600 (100), 225.0758 (13)
C127	Elenolic acid (V)	241.0721 [M-H]⁻: 95.0506 (58), 101.0247 (31), 121.0298 (21), 127.0403 (83), 139.0402 (100), 165.0558 (30), 209.0457 (16)<br>243.0864 [M+H]⁺: 211.0600 (100), 225.0757 (94)
C128	Elenolic acid glucoside (I)	403.1252 [M-H]⁻: 179.0561 (30), 179.0720 (2), 223.0612 (100), 333.0825 (15), 359.1348 (14), 371.0981 (18)
C129	Elenolic acid glucoside (II)	403.1253 [M-H]⁻: 179.0562 (26), 179.0721 (7), 223.0613 (59), 371.0984 (100)
C130	Elenolic acid diglucoside	611.1834 [M-H+HCOOH]⁻: 403.1246 (100)<br>403.1251 [M-H-hexose]⁻: 179.0560 (27), 223.0612 (100), 333.0815 (12), 359.1339 (16), 371.0981 (17)
C131	Desoxy-elenolic acid	225.0771 [M-H]⁻: 89.0247 (100)<br>227.0916 [M+H]⁺: 109.0280 (26), 141.0544 (62), 155.0702 (25), 177.0543 (12), 183.0654 (11), 185.0807 (62), 195.0650 (30), 209.0807 (100)
C132	Hydroxy-elenolic acid (I)	257.0669 [M-H]⁻: 137.0610 (100), 181.0507 (4), 213.0769 (4), 225.0406 (14)<br>259.0818 [M+H]⁺: 227.0549 (100), 241.0708 (58)
C133	Hydroxy-elenolic acid (II)	257.0671 [M-H]⁻: 137.0610 (65), 181.0507 (100), 213.0769 (81), 225.0406 (11)<br>259.0815 [M+H]⁺: 227.0551 (61), 241.0706 (100)
C134	Decarboxy-hydroxy-elenolic acid (I)	213.077 [M-H]⁻: 151.0777 (0), 169.0871 (100)<br>215.0917 [M+H]⁺: 155.0704 (17), 197.0810 (100)
C135	Decarboxy-hydroxy-elenolic acid (II)	213.0773 [M-H]⁻: 107.0869 (52), 139.0767 (50), 151.0766 (3), 169.0872 (100)<br>215.0915 [M+H]⁺: 197.0806 (100)

C	Compound	MS² and pseudo-MSⁿ spectra
C136	Decarboxy-hydroxy-elenolic acid (III)	213.0771 [M-H]⁻: 139.0767 (4), 151.0766 (100), 169.0871 (10), 183.0664 (29), 195.0665 (11)<br>215.0917 [M+H]⁺: 155.0702 (100), 197.0807 (26)
C137	Decarboxy-hydroxy-elenolic acid (IV)	213.0772 [M-H]⁻: 107.0869 (14), 139.0767 (25), 151.0766 (100), 169.0872 (12), 183.0664 (70), 195.0664 (19)<br>215.0914 [M+H]⁺: 155.0701 (100), 197.0807 (29)
C138	Decarboxy-hydroxy-elenolic acid (V)	213.0771 [M-H]⁻: 139.0763 (1), 151.0766 (100), 169.0878 (1)<br>215.0917 [M+H]⁺: 155.0702 (100), 197.0808 (24)
C139	Decarboxy-hydroxy-elenolic acid (VI)	213.0772 [M-H]⁻: 139.0767 (9), 151.0765 (100), 169.0871 (75), 183.0663 (51)<br>215.0914 [M+H]⁺: 155.0702 (100), 197.0808 (36)
C142	Decarboxy-hydroxy-elenolic acid linked to hydroxytyrosol (III)	349.1296 [M-H]⁻: 213.0769 (100), 331.1190 (19)<br>213.0772 [M-H-hydroxytyrosol]⁻: 151.0765 (100), 169.0870 (12), 183.0663 (33), 195.0663 (13)<br>351.1442 [M+H]⁺: 137.0596 (100)
C143	Decarboxy-hydroxy-elenolic acid linked to hydroxytyrosol (IV)	349.1295 [M-H]⁻: 151.0765 (41), 213.0769 (14), 331.1188 (100)<br>213.0771 [M-H-hydroxytyrosol]⁻: 151.0765 (100), 195.0663 (12)<br>351.1442 [M+H]⁺: 137.0596 (100), 333.1335 (46)
C144	Decarboxy-hydroxy-elenolic acid linked to hydroxytyrosol (V)	349.1297 [M-H]⁻: 151.0765 (25), 213.0769 (20), 331.1189 (100)<br>351.1436 [M+H]⁺: 137.0594 (100), 215.0913 (36)
C145	Methyl-elenolic acid (I)	255.0877 [M-H]⁻: 95.0505 (73), 115.0403 (37), 121.0298 (24), 139.0402 (98), 141.0559 (100), 165.0557 (36), 209.0456 (23)<br>257.102 [M+H]⁺: 211.0600 (100), 239.0913 (89)
C146	Methyl-elenolic acid (II)	257.1022 [M+H]⁺: 225.0758 (100)
C147	Aldehydic form of decarboxymethyl elenolic acid (I)	217.1074 [M+H]⁺: 121.0646 (32), 169.0860 (12), 187.0961 (19), 199.0963 (100)
C148	Aldehydic form of decarboxymethyl elenolic acid (II)	217.1071 [M+H]⁺: 121.0644 (45), 169.0858 (38), 187.0962 (97), 199.0962 (100)
C149	Aldehydic form of decarboxymethyl elenolic acid (III)	215.0928 [M-H]⁻: 139.0767 (22), 183.0664 (100)<br>217.1073 [M+H]⁺: 199.0965 (100)
C150	Aldehydic form of decarboxymethyl elenolic acid glucoside	377.1459 [M-H]⁻: 153.0923 (24), 197.0820 (100)<br>379.1602 [M+H]⁺: 199.0965 (100), 217.1075 (19)
C151	Elenolic acid dialdehyde epimer linked to hydroxytyrosol glucoside	541.1929 [M-H]⁻: 225.0768 (10), 361.1294 (100)<br>363.144 [M+H-hexose-H_2O]⁺: 137.0596 (100)

C	Compound	MS² and pseudo-MSⁿ spectra
C152	Elenolic acid dialdehyde epimer linked to hydroxytyrosol (I)	379.1403 [M-H]⁻: 151.0401 (23), 199.0612 (100), 333.0979 (27)
C153	Elenolic acid dialdehyde epimer linked to hydroxytyrosol (II)	379.1405 [M-H]⁻: 211.0614 (27), 243.0876 (100)<br>381.154 [M+H]⁺: 137.0594 (48), 227.0913 (16), 245.1015 (10), 349.1281 (100)
C154	Hydrated product of methyl-decarboxy-hydroxy-elenolic acid (I)	245.1035 [M-H]⁻: 107.0869 (20), 139.0767 (14), 151.0765 (16), 169.0871 (35), 183.0663 (31), 213.0769 (100), 215.0924 (24), 227.0926 (13)
C155	Hydrated product of methyl-decarboxy-hydroxy-elenolic acid (II)	245.1032 [M-H]⁻: 111.0818 (29), 155.0715 (14), 201.1132 (100)
C156	Hydrated product of methyl-decarboxy-hydroxy-elenolic acid (III)	245.1032 [M-H]⁻: 111.0818 (37), 155.0716 (24), 201.1133 (100), 227.0926 (12)
C158	Cannizzaro-like product of elenolic acid dialdehyde (II)	259.0826 [M-H]⁻: 95.0505 (61), 101.0246 (15), 113.0611 (97), 127.0403 (16), 139.0402 (11), 165.0558 (14), 215.0563 (24), 227.0562 (11), 241.0718 (100)
C159	DEDA (I)	183.0664 [M-H]⁻: 95.0868 (23), 139.0768 (100)<br>185.081 [M+H]⁺: 121.0645 (60), 125.0594 (31), 149.0595 (7), 167.0701 (100)
C160	DEDA (II)	183.0666 [M-H]⁻: 59.0142 (28), 95.0872 (5), 139.0765 (17), 165.0559 (100)
C161	DEDA (III)	183.0665 [M-H]⁻: 95.0870 (7), 139.0767 (100)<br>185.0809 [M+H]⁺: 95.0854 (26), 121.0647 (4), 123.0440 (15), 149.0597 (9), 167.0703 (100), 185.0809 (13)
C162	DEDA (IV)	183.0665 [M-H]⁻: 95.0870 (41), 139.0767 (100), 183.0664 (14)<br>185.081 [M+H]⁺: 121.0646 (100), 149.0598 (0), 167.0703 (5)
C163	Hydroxy-DEDA	199.0614 [M-H]⁻: 111.0091 (26), 111.0818 (100), 155.0715 (83), 181.0507 (32)<br>201.0761 [M+H]⁺: 165.0545 (44), 183.0650 (100)
C164	DEDA hydrated (I)	201.077 [M-H]⁻: 95.0869 (19), 113.0611 (15), 139.0767 (31), 157.0871 (100), 183.0663 (20)
C166	DEDA hydrated (III)	201.0771 [M-H]⁻: 139.0766 (35), 157.0872 (100), 183.0664 (24)<br>220.118 [M+NH₄]⁺: 90.0547 (46), 184.0968 (29), 202.1073 (100)
C167	DEDA hydrated (IV)	201.0772 [M-H]⁻: 139.0767 (39), 157.0872 (100), 183.0664 (27)
C168	DEDA ester (I)	197.0822 [M-H]⁻: 151.0402 (100)
C169	DEDA ester (II)	197.0822 [M-H]⁻: 153.0922 (100)
C170	DEDA ester (III)	197.082 [M-H]⁻: 109.1024 (20), 153.0921 (100)

C	Compound	MS^2 and pseudo-MS^n spectra
C171	DEDA alditol (I)	347.1352 $[M-H]^-$: 139.0768 (18), 163.0613 (13), 181.0719 (100), 183.0665 (1), 277.0924 (10)<br>349.1495 $[M+H]^+$: 121.0645 (16), 185.0811 (26), 313.1286 (58), 331.1393 (100)<br>366.1758 $[M+NH_4]^+$: 331.1391 (100)
C172	DEDA alditol (II)	347.1352 $[M-H]^-$: 123.0454 (16), 139.0767 (58), 163.0613 (29), 165.0558 (13), 181.0719 (100), 183.0662 (4), 277.0928 (16), 329.1240 (22)
C173	DEDA alditol (III)	347.1352 $[M-H]^-$: 123.0452 (10), 139.0767 (56), 163.0613 (19), 165.0558 (54), 181.0719 (100), 183.0664 (21), 277.0930 (31), 329.1247 (17)<br>349.1501 $[M+H]^+$: 121.0648 (34), 185.0809 (24), 313.1286 (56), 331.1392 (100)
C174	DEDA alditol (IV)	347.1352 $[M-H]^-$: 139.0768 (46), 163.0608 (26), 165.0559 (50), 181.0717 (100), 183.0667 (47), 185.0817 (13), 277.0924 (17), 329.1237 (22)<br>349.1497 $[M+H]^+$: 121.0646 (33), 185.0808 (24), 313.1283 (54), 331.1388 (100)
C175	DEDA alditol (V)	393.1413 $[M-H+HCOOH]^-$: 139.0763 (2), 183.0662 (100)<br>349.1499 $[M+H]^+$: 121.0646 (22), 185.0808 (14), 313.1283 (100), 331.1390 (52)
C176	Loganin	435.1511 $[M-H+HCOOH]^-$: 313.1294 (100), 357.1194 (54), 375.1299 (15)<br>391.1603 $[M+H]^+$: 133.0647 (5), 151.0748 (6), 169.0857 (5), 211.0964 (100), 229.1071 (27)<br>229.1074 $[M+H-hexose]^+$: 155.0703 (23), 169.0859 (100), 179.0701 (1), 197.0809 (11), 211.0964 (34)
C177	Loganin aglycone (I)	229.1073 $[M+H]^+$: 151.0751 (5), 155.0702 (20), 169.0858 (21), 179.0703 (3), 197.0808 (8), 211.0965 (100)<br>211.0968 $[M+H-H_2O]^+$: 133.0647 (31), 137.0596 (44), 151.0753 (100), 155.0704 (1), 169.0859 (5), 179.0703 (75), 181.0859 (14), 183.1016 (15), 193.0859 (51), 197.0811 (1)
C178	Loganin aglycone (II)	229.1072 $[M+H]^+$: 155.0701 (66), 169.0858 (100), 197.0805 (17), 211.0964 (41)<br>211.0968 $[M+H-H_2O]^+$: 133.0648 (37), 151.0752 (39), 161.0596 (26), 169.0858 (2), 179.0704 (35), 181.0860 (18), 193.0859 (100), 197.0805 (2)
C179	Loganin aglycone (III)	229.1073 $[M+H]^+$: 155.0702 (2), 169.0857 (12), 179.0696 (1), 197.0804 (1), 211.0965 (100)<br>211.0966 $[M+H-H_2O]^+$: 127.0387 (19), 141.0544 (20), 153.0544 (11), 171.0650 (100)
C180	Loganin aglycone (IV)	227.093 $[M-H]^-$: 111.0091 (15), 181.0505 (100)<br>229.1073 $[M+H]^+$: 165.0545 (34), 183.0649 (32), 211.0963 (100)
C181	Hydrated product of loganin	407.1566 $[M-H]^-$: 313.1295 (38), 357.1195 (52), 375.1302 (56), 377.1455 (19), 389.1455 (100)<br>409.1707 $[M+H]^+$: 169.0859 (31), 211.0966 (22), 229.1071 (100)<br>229.1073 $[M+H-hexose-H_2O]^+$: 155.0701 (35), 169.0858 (92), 197.0807 (13), 211.0964 (100)
C183	Hydroxytyrosol derivative 02	447.1512 $[M-H]^-$: 135.0453 (33), 315.1084 (100)
C184	Hydroxytyrosol derivative 03 (I)	323.1503 $[M-H]^-$: 187.0975 (100)
C185	Hydroxytyrosol derivative 03 (II)	323.1505 $[M-H]^-$: 139.0765 (13), 187.0976 (100)<br>325.1646 $[M+H]^+$: 137.0595 (100)

C	Compound	MS^2 and pseudo-MS^n spectra
C186	Hydroxytyrosol derivative 04	463.125 $[M-H]^-$: 291.0507 (100), 327.0719 (30)
C187	Hydroxytyrosol derivative 05	279.1241 $[M-H]^-$: 143.0715 (100)
C188	Hydroxytyrosol derivative 06	453.1558 $[M-H]^-$: 151.0402 (17), 203.0713 (11), 255.1027 (100), 273.1134 (65), 275.0924 (13), 299.0922 (23), 317.1032 (67), 383.1138 (67), 435.1454 (29)
C189	Hydroxytyrosol derivative 07	393.1923 $[M-H]^-$: 257.1394 (100)
C190	DEDA derivative 01	483.1879 $[M-H]^-$: 165.0558 (99), 183.0664 (100)
C191	DEDA derivative 02	357.1197 $[M-H]^-$: 165.0559 (100), 183.0664 (43), 339.1083 (11)<br>376.1606 $[M+NH_4]^+$: 107.0482 (12), 167.0702 (100), 171.1022 (18), 199.1169 (21)
C192	DEDA derivative 03 (I)	499.1822 $[M-H]^-$: 199.0611 (100)
C193	DEDA derivative 03 (II)	518.2235 $[M+NH_4]^+$: 167.0700 (9), 185.0806 (7), 303.1227 (14), 321.1329 (100), 329.1232 (11), 339.1433 (25)
C194	DEDA derivative 04 (I)	367.1402 $[M-H]^-$: 139.0767 (14), 183.0663 (100)
C195	DEDA derivative 04 (II)	367.1402 $[M-H]^-$: 139.0766 (15), 153.0558 (36), 183.0663 (66), 197.0455 (46), 321.1343 (24), 349.1284 (100)
C196	DEDA derivative 05	351.1455 $[M-H]^-$: 139.0766 (4), 165.0556 (1), 183.0663 (41), 215.0924 (83), 319.1186 (100)
C197	DEDA derivative 05 linked to hydroxytyrosol	487.1976 [M-H]-: 165.0558 (0), 183.0668 (0), 351.1451 (100)
C198	Elenolic acid derivative 01	159.0667 $[M-H+HCOOH]^-$: 95.0505 (10), 113.0610 (26), 121.0298 (100), 123.0454 (59), 141.0559 (38)<br>132.1018 $[M+NH_4]^+$: 86.0962 (100)
C199	Elenolic acid derivative 02 (I)	274.0926 $[M+NH_4]^+$: 130.0497 (15), 226.0712 (36), 238.0711 (100), 256.0817 (89)
C200	Elenolic acid derivative 02 (II)	255.0512 $[M-H]^-$: 165.0558 (100), 193.0506 (25), 209.0460 (1)
C201	Elenolic acid derivative 03	555.209 $[M-H]^-$: 165.0559 (3), 183.1027 (15), 197.1183 (12), 209.0456 (12), 225.1132 (15), 327.1083 (14), 345.1192 (45), 511.2188 (100)
C202	Elenolic acid derivative 04	493.1353 $[M-H]^-$: 135.0453 (11), 165.0558 (10), 209.0456 (17), 327.1084 (22), 449.1452 (100)<br>495.1507 $[M+H]^+$: 151.0387 (53), 211.0600 (61), 229.0708 (35), 231.0655 (16), 249.0753 (25), 267.0864 (100), 477.1394 (78)
C203	Unknown 001 (glucoside)	292.1404 $[M-H]^-$: 130.0877 (81), 191.1074 (28), 202.1085 (100), 244.1188 (55)<br>294.1549 $[M+H]^+$: 276.1442 (100)
C204	Unknown 002 (glucoside)	323.1707 $[M+H]^+$: 143.1066 (76), 305.1600 (100)
C205	Unknown 003 (glucoside)	301.093 $[M-H]^-$: 101.0246 (21), 113.0246 (16), 124.0168 (23), 139.0402 (63), 159.0303 (10), 161.0457 (100)
C206	Unknown 004 glucoside	333.1547 $[M+H]^+$: 111.0802 (15), 153.0909 (91), 171.1016 (100)
C208	Unknown 005 (glucoside)	467.1775 $[M-H+HCOOH]^-$: 371.1351 (100)<br>423.1867 $[M+H]^+$: 243.1231 (100)

C	Compound	MS² and pseudo-MSⁿ spectra
C209	Unknown 006 (glucoside)	387.2015 $[M+H]^+$: 189.1268 (17), 207.1380 (100), 369.1906 (21)
C210	Unknown 007 (glucoside)	403.1978 $[M-H]^-$: 161.0457 (13), 223.1340 (20), 241.1445 (100)
C211	Unknown 008 (glucoside) (I)	523.181 $[M+H]^+$: 361.1283 (100), 491.1547 (40)
C213	Unknown 009 (glucoside)	553.157 $[M-H]^-$: 167.0350 (84), 209.0456 (85), 289.0352 (13), 289.0714 (39), 321.0613 (90), 341.0671 (22), 359.0770 (76), 373.0932 (23), 391.1035 (100), 521.1313 (30)
C214	Unknown 010 (glucoside)	691.2249 $[M-H]^-$: 377.1238 (12), 403.1244 (14), 459.1296 (25), 497.1452 (16), 511.1603 (2), 529.1714 (18), 539.1766 (100)
C215	Unknown 011 glucoside	583.2038 $[M-H]^-$: 223.0614 (13), 403.1249 (79), 421.1505 (17), 537.1613 (100)
C216	Unknown 011	421.1508 $[M-H]^-$: 151.0400 (23), 223.0614 (12), 319.1185 (94), 351.1083 (100), 389.1243 (34)
C217	Unknown 012 (glucoside)	729.2982 $[M-H]^-$: 387.1810 (51), 549.2337 (100), 567.2433 (6)
C218	Unknown 013 (I)	266.1037 $[M-H]^-$: 222.1136 (100)<br>268.1185 $[M+H]^+$: 120.0807 (38), 138.0912 (100)
C219	Unknown 013 (II)	266.1035 $[M-H]^-$: 178.0874 (45), 222.0773 (100)<br>268.1184 $[M+H]^+$: 236.0918 (16), 250.1076 (100)
C221	Unknown 014 (II)	189.1122 $[M+H]^+$: 141.0909 (65), 159.1011 (85), 171.1014 (100)
C222	Unknown 014 (III)	187.0978 $[M-H]^-$: 95.0869 (40), 169.0871 (100)
C223	Unknown 014 (IV)	187.0977 $[M-H]^-$: 95.0869 (37), 169.0871 (100)
C224	Unknown 015 (I)	185.0820 $[M-H]^-$: 111.0818 (100)
C225	Unknown 015 (II)	185.0822 $[M-H]^-$: 141.0923 (100)
C226	Unknown 016 (I)	461.1672 $[M-H+HCOOH]^-$: 179.0713 (35), 415.1612 (100)<br>434.2038 $[M+NH_4]^+$: 416.1707 (100)
C227	Unknown 016 (II)	415.1617 $[M-H]^-$: 131.0352 (28), 149.0457 (100), 191.0563 (26), 251.0775 (12), 293.0878 (15), 311.0982 (12)
C228	Unknown 017 (I)	583.2034 $[M-H+HCOOH]^-$: 375.1450 (11), 537.1974 (100)
C230	Unknown 018 (I)	507.2805 $[M+H]^+$: 165.0551 (13), 225.0756 (100), 475.1602 (44)
C231	Unknown 018 (II)	505.2648 $[M-H]^-$: 373.2230 (100)
C232	Unknown 019 (I)	363.1452 $[M-H]^-$: 191.0971 (14), 191.1177 (18), 191.1352 (11), 201.1653 (25), 231.1755 (50), 245.1542 (16), 275.1651 (100), 319.1546 (77)<br>365.1599 $[M+H]^+$: 221.0807 (14), 283.1330 (14), 301.1434 (70), 305.1385 (65), 319.1543 (86), 329.1385 (68), 347.1488 (100)
C234	Unknown 020 (I)	197.1174 $[M+H]^+$: 135.1167 (68), 161.0960 (16), 179.1067 (100)

C	Compound	MS^2 and pseudo-MS^n spectra
C235	Unknown 020 (II)	197.1176 $[M+H]^+$: 151.0753 (100)
C236	Unknown 021 (I)	579.2092 $[M-H]^-$: 241.0724 (12), 293.1028 (41), 337.1290 (100)
C238	Unknown 022 (I)	307.1544 $[M+H]^+$: 137.0595 (100), 171.1015 (66)
C239	Unknown 022 (II)	305.1394 $[M-H]^-$: 89.0056 (12), 89.0089 (11), 89.0537 (11), 123.0819 (93), 137.0610 (100), 167.0718 (24), 185.0822 (77), 191.1026 (17), 191.1262 (15)<br>307.1542 $[M+H]^+$: 85.0641 (11), 121.0646 (100), 271.0413 (12)
C241	Unknown 023 (II)	349.1644 $[M+H]^+$: 137.0594 (100), 167.0702 (15), 195.1012 (12), 213.1121 (32), 277.1067 (17), 303.1227 (73), 331.1546 (42), 340.1951 (16)
C242	Unknown 024 (I)	485.1827 $[M-H]^-$: 227.1079 (23), 243.1392 (100), 271.0977 (13), 287.1288 (67), 407.1502 (23), 423.1811 (13), 467.1717 (12)
C243	Unknown 024 (II)	485.1827 $[M-H]^-$: 227.1077 (26), 243.1392 (100), 271.0978 (13), 287.1288 (68), 407.1502 (17)
C244	Unknown 025 (I)	227.1290 $[M-H]^-$: 165.1287 (100), 183.1391 (82)
C245	Unknown 025 (II)	227.1293 $[M-H]^-$: 183.1391 (100), 209.1185 (23)<br>229.1439 $[M+H]^+$: 193.1223 (37), 211.1330 (100)
C246	Unknown 026 (I)	577.2690 $[M-H]^-$: 184.0926 (10), 184.1798 (11), 225.0075 (100), 243.0180 (12), 299.0438 (47), 342.1432 (22)
C247	Unknown 026 (II)	577.2690 $[M-H]^-$: 225.0074 (100), 299.0439 (77)
C248	Unknown 027	272.9593 $[M-H]^-$: 158.9787 (100), 228.9691 (17)
C249	Unknown 028	102.0338 $[M+H]^+$: 61.0070 (100), 70.0123 (33), 81.5203 (77), 90.5257 (24)
C253	Unknown 032	290.0765 $[M+H]^+$: 200.0440 (100)
C255	Unknown 034	116.0705 $[M+H]^+$: 56.0082 (10), 56.0138 (16), 56.0257 (15), 70.0648 (100), 85.1075 (13)
C256	Unknown 035	191.0737 $[M-H]^-$: 85.0298 (19), 93.0349 (11), 117.0367 (100), 127.0403 (16)
C258	Unknown 037	317.0549 $[M-H]^-$: 125.0245 (12), 225.0075 (100)
C263	Unknown 042	305.1349 $[M+Na]^+$: 227.1027 (50), 233.0921 (18), 251.1027 (24), 269.1133 (40), 287.1240 (100)
C264	Unknown 043	512.1985 $[M+NH_4]^+$: 171.0651 (100), 297.0974 (12), 333.1180 (28)
C266	Unknown 045	333.1196 $[M-H]^-$: 151.0402 (29), 181.0719 (100)
C267	Unknown 046	147.0303 $[M-H]^-$: 57.0349 (24), 85.0298 (100), 87.0090 (64), 129.0196 (59)
C271	Unknown 050	217.0720 $[M-H]^-$: 129.0560 (11), 173.0457 (100), 199.0613 (22)
C273	Unknown 052	418.1512 $[M-H]^-$: 374.1610 (100)
C276	Unknown 055	252.1234 $[M+NH_4]^+$: 166.0862 (100)

C	Compound	MS^2 and pseudo-MS^n spectra
C280	Unknown 059	310.0936 $[M-H]^-$: 115.0040 (95), 150.0563 (14), 194.0823 (84), 222.1140 (26), 266.1035 (100)<br>312.1089 $[M+H]^+$: 250.1077 (19), 266.1025 (26), 268.1182 (18), 280.0817 (11), 294.0976 (100)
C281	Unknown 060	275.0773 $[M-H]^-$: 155.0351 (41), 199.0611 (100), 229.0719 (23), 243.0509 (14)
C282	Unknown 061	161.0458 $[M-H]^-$: 71.0142 (49), 115.0039 (15), 143.0350 (100)
C283	Unknown 062	147.0665 [M-H]-: 57.0349 (53), 69.1375 (12), 69.1655 (12), 69.1874 (10), 92.0751 (10), 99.0090 (38), 101.0247 (100), 101.0609 (15), 129.0561 (14)
C284	Unknown 063	280.1190 $[M-H]^-$: 150.0562 (27), 194.0823 (100)<br>282.1338 $[M+H]^+$: 178.0862 (29), 196.0968 (100)
C285	Unknown 064	381.1406 $[M-H]^-$: 155.0715 (41), 181.0717 (39), 199.0612 (100), 363.1297 (19)
C286	Unknown 065	326.1242 $[M+H]^+$: 251.0754 (100)
C288	Unknown 067	375.1298 $[M-H]^-$: 331.1398 (100)
C289	Unknown 068	755.2993 $[M-H]^-$: 347.1345 (100), 389.1461 (12), 407.1558 (82)
C291	Unknown 070	181.0509 $[M-H]^-$: 135.0453 (12), 163.0402 (100)<br>183.0577 $[M+H]^+$: 85.0745 (20), 123.0438 (100), 135.0060 (12), 155.0462 (11), 165.0545 (62), 199.1991 (13)
C294	Unknown 073	196.0972 $[M+NH_4]^+$: 178.0864 (100)
C295	Unknown 074	252.1600 $[M+NH_4]^+$: 120.0806 (78), 166.1225 (100), 206.1176 (22)
C296	Unknown 075	175.0615 $[M-H]^-$: 85.0661 (88), 113.0611 (53), 115.0403 (100), 157.0508 (38)
C298	Unknown 077	431.1567 $[M-H]^-$: 113.0246 (14), 131.0351 (27), 149.0456 (58), 191.0561 (26), 299.1134 (100)<br>433.1607 $[M+H]^+$: 318.1345 (10), 389.1713 (100), 415.1504 (25), 416.1345 (42)
C299	Unknown 078	348.1656 $[M+NH_4]^+$: 151.0753 (100), 169.0859 (16), 265.1072 (10), 277.1072 (36), 295.1176 (40), 331.1389 (34)
C300	Unknown 079	266.1390 $[M+NH_4]^+$: 180.1018 (100)
C302	Unknown 081	344.1343 $[M+NH_4]^+$: 147.0439 (97), 165.0545 (100), 180.0867 (12), 309.0970 (15)
C303	Unknown 082	390.1761 $[M+NH_4]^+$: 161.0595 (27), 193.0857 (100)
C304	Unknown 083	611.1835 $[M-H]^-$: 403.1245 (81), 565.1772 (100)
C306	Unknown 085	318.1703 $[M+NH_4]^+$: 272.1281 (100)
C307	Unknown 086	401.1455 $[M-H]^-$: 161.0458 (20), 269.1030 (100)<br>420.1852 $[M+NH_4]^+$: 115.0388 (51), 133.0494 (62), 223.0965 (74), 241.0704 (13), 253.1076 (18), 259.0813 (70), 295.1024 (100)

C	Compound	MS² and pseudo-MSⁿ spectra
C308	Unknown 087	305.0702 [M-H]⁻: 126.0624 (10), 126.1088 (11), 225.1130 (100)
		307.0852 [M+H]⁺: 85.1032 (19), 85.1163 (14), 131.1372 (12), 191.0825 (17), 192.1650 (18), 199.0713 (22), 199.0949 (14), 199.1530 (22), 227.1277 (100)
C309	Unknown 088	519.1721 [M-H]⁻: 193.0505 (100), 235.0612 (29), 265.0716 (16), 295.0821 (21), 325.1138 (32)
C311	Unknown 090	424.1827 [M+NH4]⁺: 131.1557 (12), 136.0987 (14), 136.1104 (11), 136.1274 (13), 192.1911 (11), 271.0181 (15), 340.1530 (43), 389.1442 (100)
C312	Unknown 091	302.1601 [M+NH4]⁺: 145.0494 (12), 163.0598 (14), 285.1331 (100)
C313	Unknown 092	420.2238 [M+NH4]⁺: 161.1322 (17), 205.1222 (85), 223.1328 (100), 241.1438 (24), 271.0229 (12)
C314	Unknown 093	412.2183 [M+NH4]⁺: 115.0389 (34), 133.0495 (34), 233.1387 (18), 259.0814 (44), 263.1492 (48), 295.1026 (100)
C315	Unknown 094	439.1827 [M-H]⁻: 393.1768 (100)
C317	Unknown 096	183.1017 [M+H]⁺: 123.0438 (10), 151.0752 (100)
C318	Unknown 097	551.1774 [M-H]⁻: 337.0927 (100)
C319	Unknown 098	446.1808 [M+NH4]⁺: 180.1018 (11), 206.0812 (30), 224.0917 (14), 292.1182 (100), 310.1288 (17), 360.1444 (25)
C320	Unknown 099	511.2396 [M-H]⁻: 325.1138 (100), 343.1241 (18)
		513.2547 [M+H]⁺: 277.1433 (10), 295.1539 (28), 313.1648 (22), 331.1753 (100)
C322	Unknown 101	253.0724 [M-H]⁻: 117.0196 (100)
C324	Unknown 103	441.1773 [M-H]⁻: 153.0922 (41), 197.0819 (100), 243.0875 (19)
C325	Unknown 104	775.2317 [M-H]⁻: 345.1189 (46), 371.0979 (18), 389.1089 (12), 403.1241 (12), 499.1463 (13), 525.1245 (16), 537.1605 (13)
C328	Unknown 107	543.2088 [M-H]⁻: 389.1453 (10), 513.1982 (42), 525.1980 (100)
		562.2504 [M+NH4]⁺: 365.1594 (100), 383.1706 (18)
C330	Unknown 109	319.1176 [M+H]⁺: 179.0701 (53), 241.0857 (15), 259.0962 (100), 275.0912 (100), 283.0962 (15), 301.1071 (56)
C332	Unknown 111	401.1597 [M+H]⁺: 167.0702 (22), 351.1229 (17), 369.1335 (17), 383.1489 (100)
C333	Unknown 112	291.1235 [M+H]⁺: 137.0596 (100)
C334	Unknown 113	461.1455 [M-H]⁻: 145.0296 (57), 307.0820 (100), 315.1083 (24)
C335	Unknown 114	623.1987 [M-H+HCOOH]⁻: 191.0909 (15), 191.1109 (17), 191.1265 (14), 342.0139 (26), 577.1924 (100)
		596.2350 [M+NH4]⁺: 187.0760 (10), 192.1430 (11), 199.0282 (14), 199.0901 (14), 199.1710 (14), 203.0703 (16), 233.0806 (100), 339.1219 (15), 357.1341 (41)
C337	Unknown 116	491.1558 [M-H]⁻: 337.0927 (100)
		493.1707 [M+H]⁺: 177.0545 (60), 195.0652 (10), 263.0915 (15), 299.1124 (14), 339.1074 (32), 439.1382 (10), 457.1489 (24), 475.1597 (100)
C338	Unknown 117	789.2477 [M-H]⁻: 342.0599 (30), 371.0975 (11), 403.1242 (100), 445.1353 (28), 557.1467 (12), 595.1657 (16)

C	Compound	MS² and pseudo-MSⁿ spectra
C339	Unknown 118	623.2356 [M-H]⁻: 191.1036 (12), 191.1175 (12), 191.1328 (10), 331.1212 (13), 349.1288 (19), 363.1436 (20), 381.1555 (100), 461.1654 (59)
C340	Unknown 119	484.2184 [M+NH4]⁺: 167.0703 (37), 305.1381 (12), 311.1136 (11), 329.1230 (35), 449.1808 (77), 467.1913 (100)
C342	Unknown 121	539.1257 [M+H]⁺: 231.0288 (14), 269.0810 (11), 495.1255 (10), 521.1074 (100)
C343	Unknown 122	655.2402 [M-H]⁻: 259.0976 (47), 291.0871 (100), 361.1294 (89), 517.1561 (35), 539.1765 (11)
C344	Unknown 123	717.1472 [M-H]⁻: 321.0403 (15), 519.0930 (100)<br>736.1874 [M+NH4]⁺: 521.1078 (100), 666.0363 (12), 719.1621 (29)
C347	Unknown 126	221.1901 [M+H]⁺: 95.0851 (12), 109.1011 (19), 119.0854 (16), 133.1011 (15), 135.1168 (19), 147.1168 (20), 161.1323 (16), 203.1795 (100), 212.1142 (12)
C348	Unknown 127	419.2639 [M+H]⁺: 124.1740 (13), 124.1925 (13), 131.1732 (18), 199.1484 (26), 260.0016 (25), 271.0545 (37), 340.1811 (100), 354.0295 (51)
C350	Unknown 129	601.2142 [M-H]⁻: 197.0820 (100), 403.1249 (20)
C353	Unknown 142	289.1439 [M+H]⁺: 121.0646 (100), 169.0859 (36)
C354	Unknown 143	388.1404 [M-H]⁻: 284.1292 (52), 344.1505 (100), 360.1454 (12)<br>390.1549 [M+H]⁺: 178.0863 (15), 222.0760 (100)
C357	Unknown 146	393.1196 [M-H]⁻: 317.1030 (100), 349.1286 (19), 361.0931 (12)
C361	Unknown 150	471.1665 M-H]⁻: 165.0558 (17), 195.0663 (100), 319.1187 (27)<br>490.2079 [M+NH4]⁺: 137.0594 (38), 271.0309 (10), 303.1230 (100), 321.1333 (31), 455.1699 (11)
C362	Unknown 151	319.1192 [DOA-H]⁻: 165.0558 (22), 195.0663 (100)
C363	Unknown 152	331.2494 [M-H]⁻: 171.1028 (13)
C364	Unknown 153	609.2350 [M-H]⁻: 473.1817 (100)
C367	Unknown 156	287.2229 [M-H]⁻: 267.1966 (14)
C368	Unknown 157	271.1180 [M+H]⁺: 137.0594 (10), 194.0592 (13), 225.0753 (12), 239.0911 (100)
C369	Unknown 158	319.1542 [M+H]⁺: 121.0646 (100)
C372	Unknown 161	345.1710 [M-H]⁻: 301.1806 (100)
C373	Unknown 162	485.2187 [M-H]⁻: 349.1657 (100)
C374	Unknown 163	721.3642 [M-H]⁻: 270.1878 (32), 342.0111 (100), 342.0580 (16), 449.1723 (16), 475.0255 (35), 598.1650 (15), 669.0986 (58)
C378	Unknown 167	555.2849 [M-H]⁻: 225.0075 (100), 299.0440 (62)

Figure S1. Scores plot of the samples in the space described by the first two principal components based on data obtained in untargeted analysis in positive (above) and negative (below) modes (samples indicated in red are the quality controls).

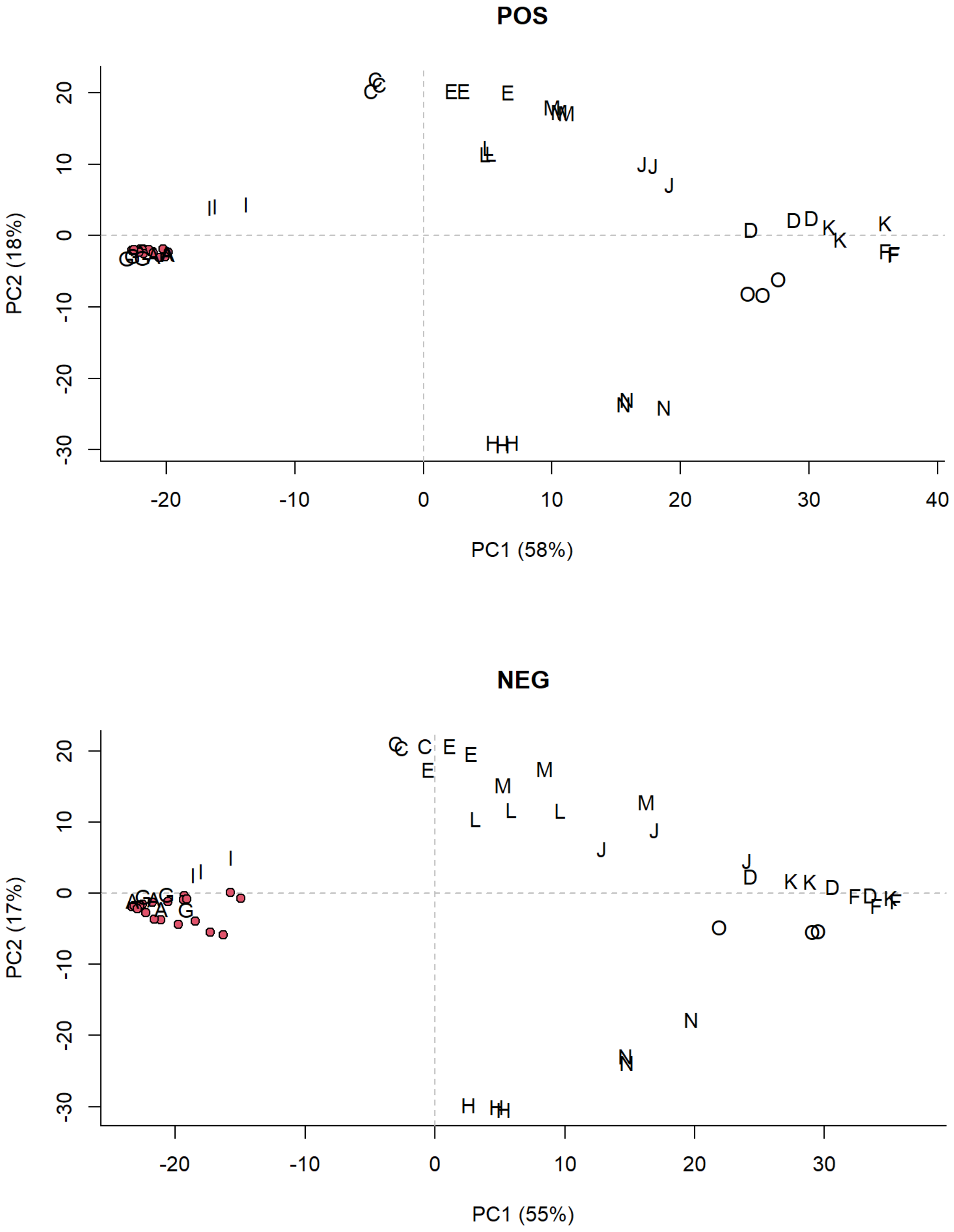

Letters refer to the different analyzed products (each one was prepared in triplicate), which characteristics are in Table 4. Red points refer to quality control samples.

Figure S2. Heat map of discriminant features according to the part of the plant used.

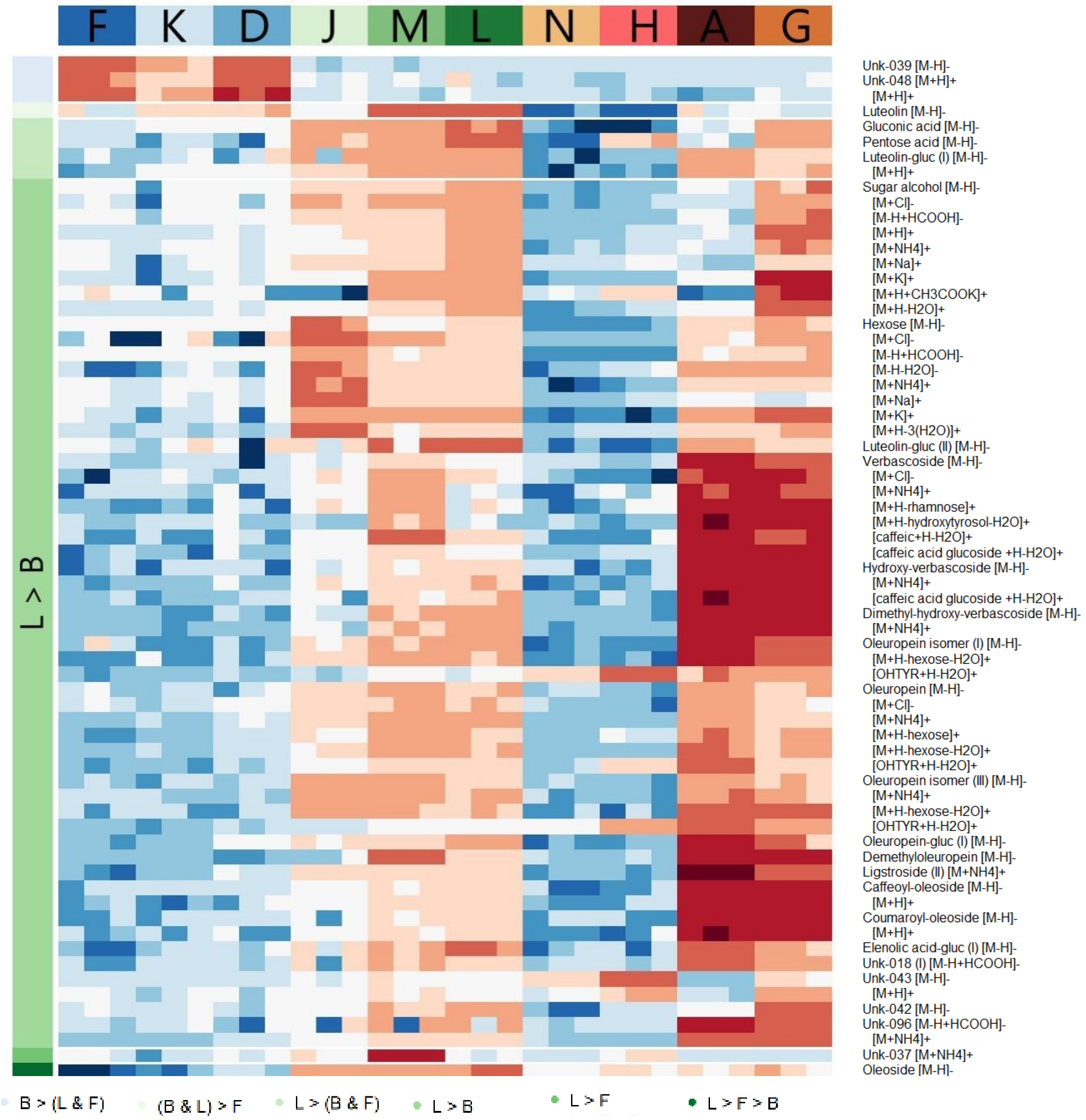

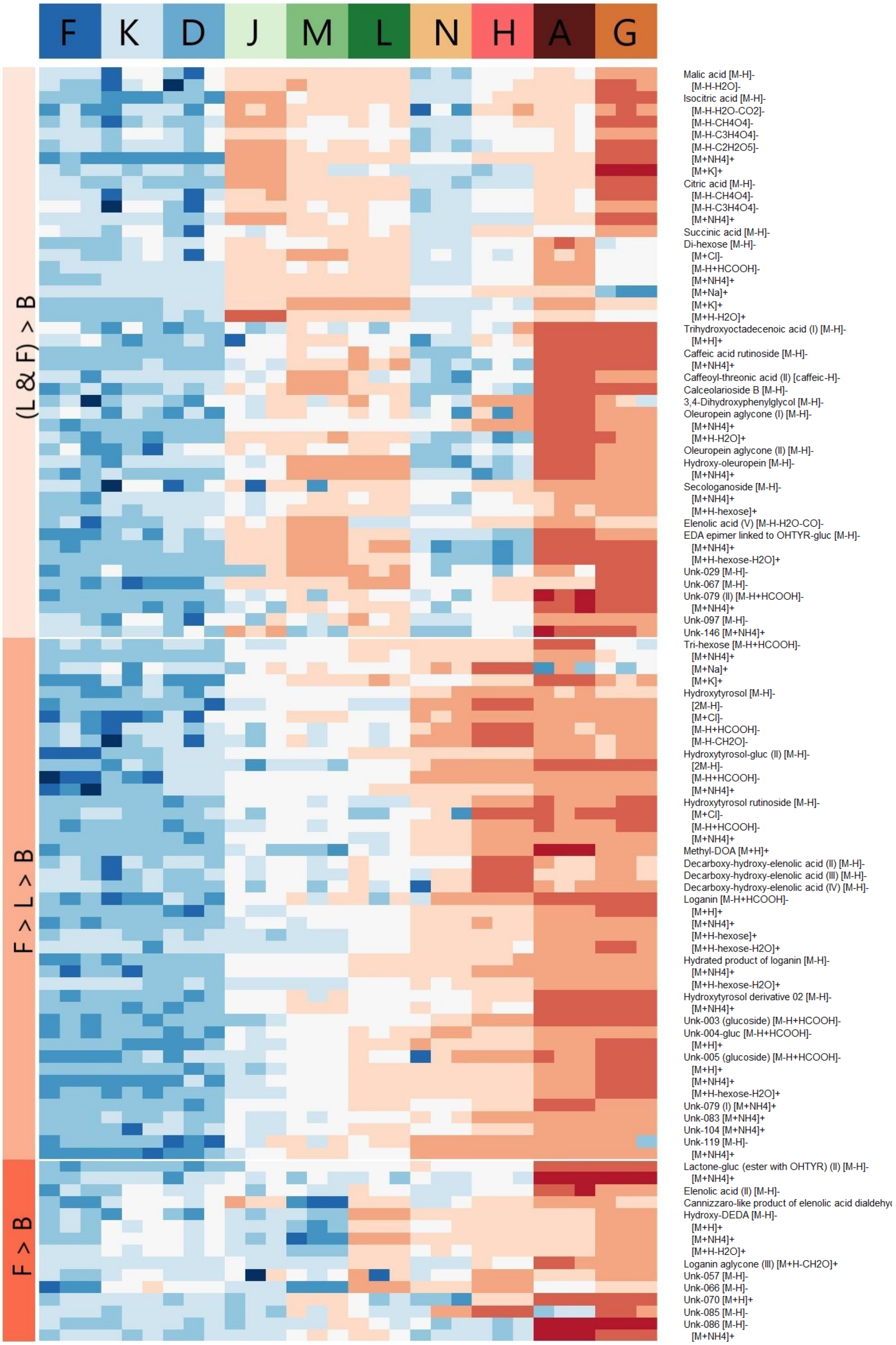

F
K
D
J
M
L
N
H
A
G
(L & F) > B
F > L > B
F > B
Malic acid [M-H]-
[M-H-H2O]-
Isocitric acid [M-H]-
[M-H-H2O-CO2]-
[M-H-CH4O4]-
[M-H-C3H4O4]-
[M-H-C2H2O5]-
[M+NH4]+
[M+K]+
Citric acid [M-H]-
[M-H-CH4O4]-
[M-H-C3H4O4]-
[M+NH4]+
Succinic acid [M-H]-
Di-hexose [M-H]-
[M+Cl]-
[M-H+HCOOH]-
[M+NH4]+
[M+Na]+
[M+K]+
[M+H-H2O]+
Trihydroxyoctadecenoic acid (I) [M-H]-
[M+H]+
Caffeic acid rutinoside [M-H]-
[M+NH4]+
Caffeoyl-threonic acid (II) [caffeic-H]-
Calceolarioside B [M-H]-
3,4-Dihydroxyphenylglycol [M-H]-
Oleuropein aglycone (I) [M-H]-
[M+NH4]+
[M+H-H2O]+
Oleuropein aglycone (II) [M-H]-
Hydroxy-oleuropein [M-H]-
[M+NH4]+
Secologanoside [M-H]-
[M+NH4]+
[M+H-hexose]+
Elenolic acid (V) [M-H-H2O-CO]-
EDA epimer linked to OHTYR-gluc [M-H]-
[M+NH4]+
[M+H-hexose-H2O]+
Unk-029 [M-H]-
Unk-067 [M-H]-
Unk-079 (II) [M-H+HCOOH]-
[M+NH4]+
Unk-097 [M-H]-
Unk-146 [M+NH4]+
Tri-hexose [M-H+HCOOH]-
[M+NH4]+
[M+Na]+
[M+K]+
Hydroxytyrosol [M-H]-
[2M-H]-
[M+Cl]-
[M-H+HCOOH]-
[M-H-CH2O]-
Hydroxytyrosol-gluc (II) [M-H]-
[2M-H]-
[M-H+HCOOH]-
[M+NH4]+
Hydroxytyrosol rutinoside [M-H]-
[M+Cl]-
[M-H+HCOOH]-
[M+NH4]+
Methyl-DOA [M+H]+
Decarboxy-hydroxy-elenolic acid (II) [M-H]-
Decarboxy-hydroxy-elenolic acid (III) [M-H]-
Decarboxy-hydroxy-elenolic acid (IV) [M-H]-
Loganin [M-H+HCOOH]-
[M+H]+
[M+NH4]+
[M+H-hexose]+
[M+H-hexose-H2O]+
Hydrated product of loganin [M-H]-
[M+NH4]+
[M+H-hexose-H2O]+
Hydroxytyrosol derivative 02 [M-H]-
[M+NH4]+
Unk-003 (glucoside) [M-H+HCOOH]-
Unk-004-gluc [M-H+HCOOH]-
[M+H]+
Unk-005 (glucoside) [M-H+HCOOH]-
[M+H]+
[M+NH4]+
[M+H-hexose-H2O]+
Unk-079 (I) [M+NH4]+
Unk-083 [M+NH4]+
Unk-104 [M+NH4]+
Unk-119 [M-H]-
[M+NH4]+
Lactone-gluc (ester with OHTYR) (II) [M-H]-
[M+NH4]+
Elenolic acid (II) [M-H]-
Cannizzaro-like product of elenolic acid dialdehy
Hydroxy-DEDA [M-H]-
[M+H]+
[M+NH4]+
[M+H-H2O]+
Loganin aglycone (III) [M+H-CH2O]+
Unk-057 [M-H]-
Unk-066 [M-H]-
Unk-070 [M+H]+
Unk-085 [M-H]-
Unk-086 [M-H]-
[M+NH4]+

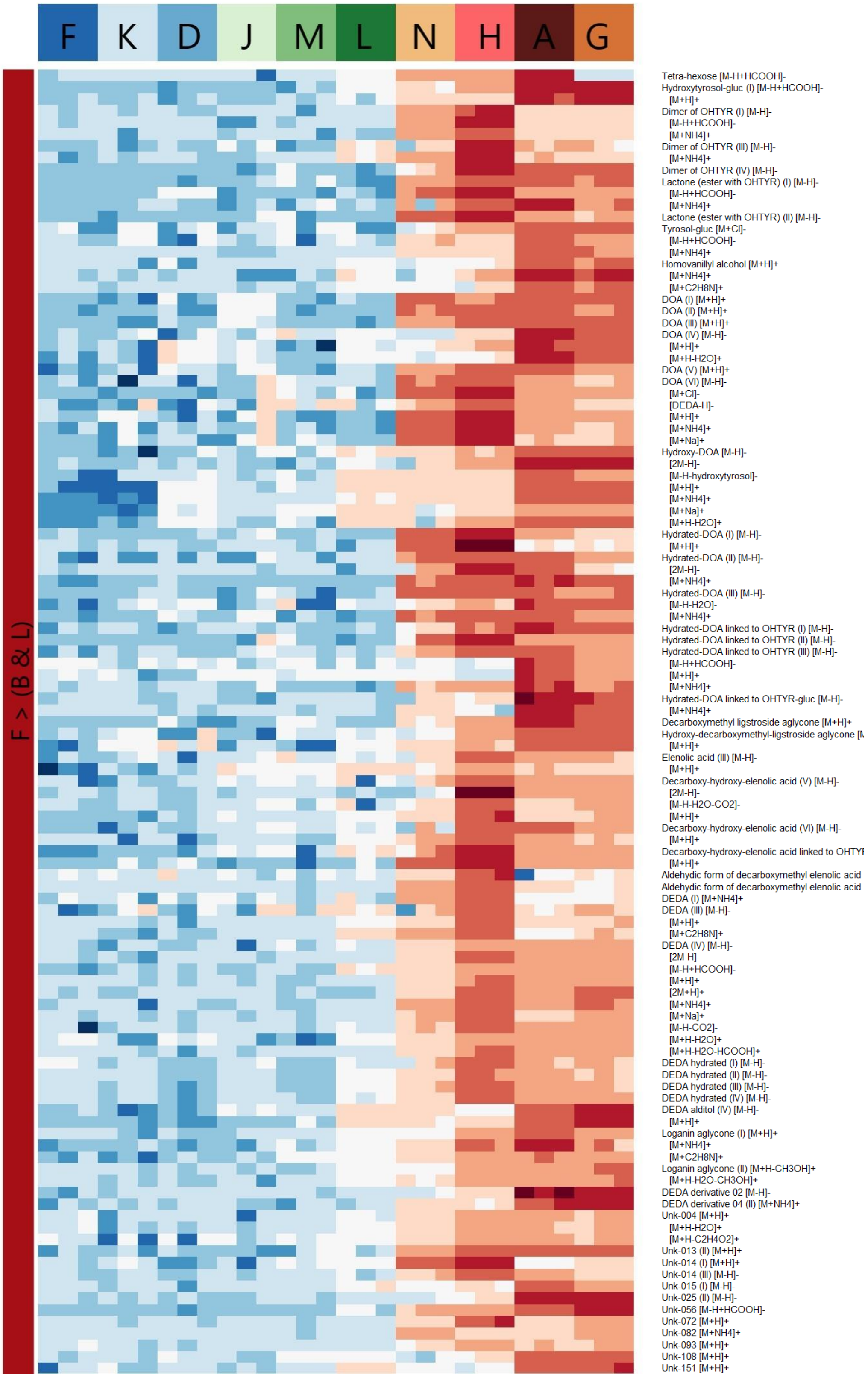

Abbreviations: B, buds-based products; F, fruit-based products; L, leave-based products.
Blue and orange cells correspond to low- and high-metabolite levels, respectively. Columns are samples (codified as indicated in Table 4), and rows are compounds colored by behavior distribution among products' classes (B, F or L). The symbology (e.g., "F > (B & L)") indicates the behavior of the compound distribution among the products' categories according to the Kruskal-Wallis test.

Figure S3. Boxplots of selected compounds characteristic of products made from a particular part of the olive plant.

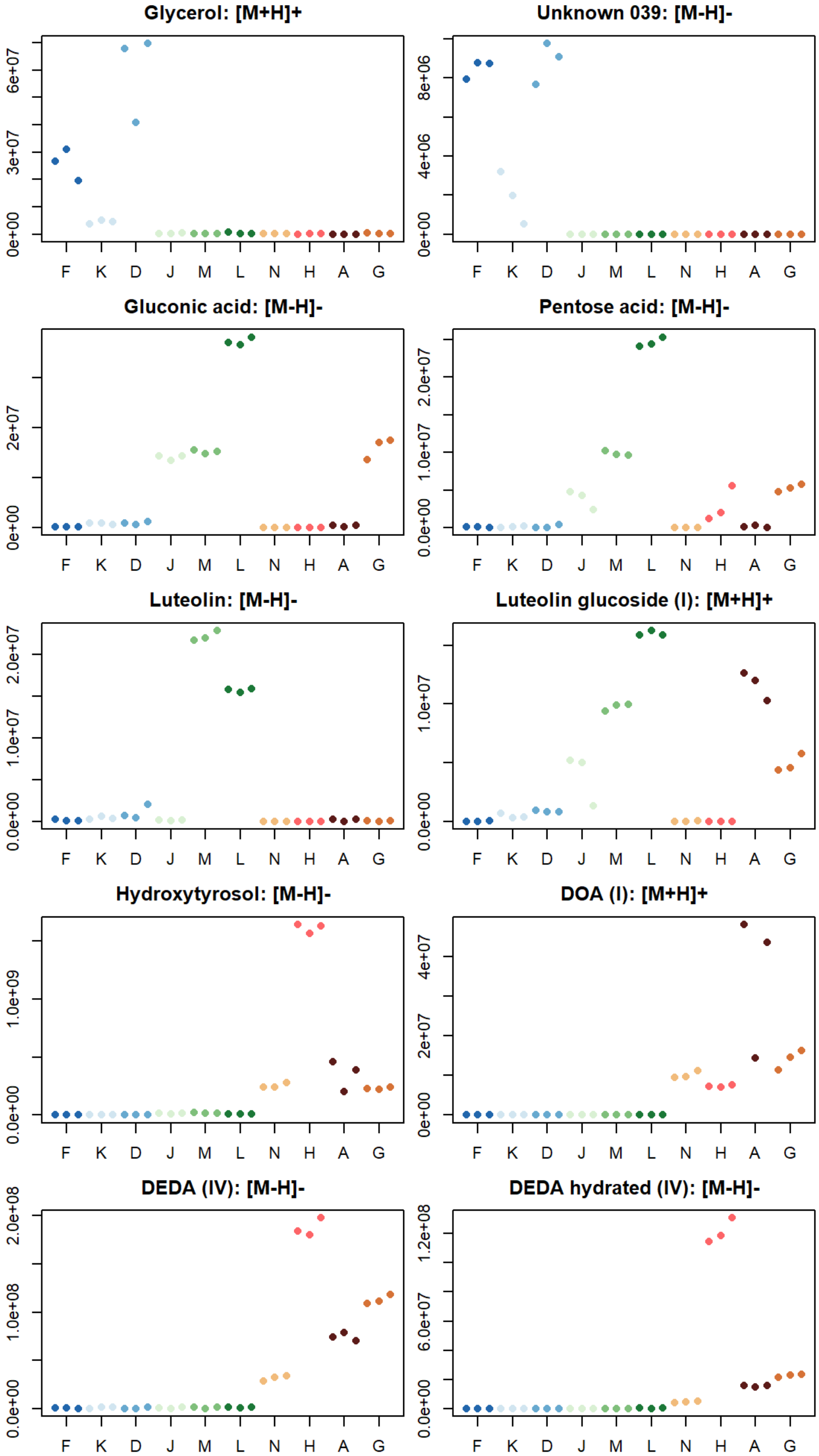

Figure S4. Pie charts of total amount of compounds by product.

product	tar-get	un-tar-get all	un-tar-get ID
K	0.1	0.2	0.2
D	0.2	0.4	0.3
F	0.1	0.2	0.2
J	0.4	2.2	2.5
M	0.7	2.3	2.0
L	0.6	2.8	2.7
C	8.5	11.4	10.6
O	0.1	0.6	0.6
E	3.9	6.0	5.9
I	19.7	17.3	18.1
N	1.4	2.2	2.1
H	16.0	14.5	14.9
A	18.5	22.6	23.5
G	29.9	17.4	16.7

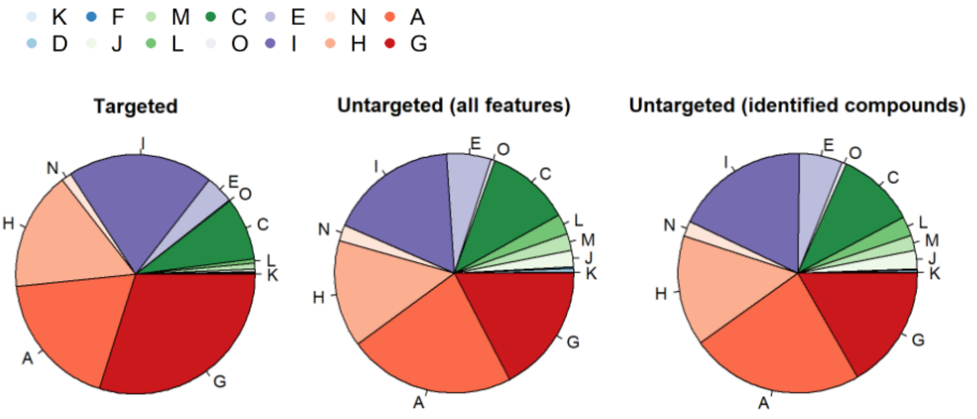

Figure S5. Pie charts of proportion of phenolic and non-phenolic compounds present in each product.

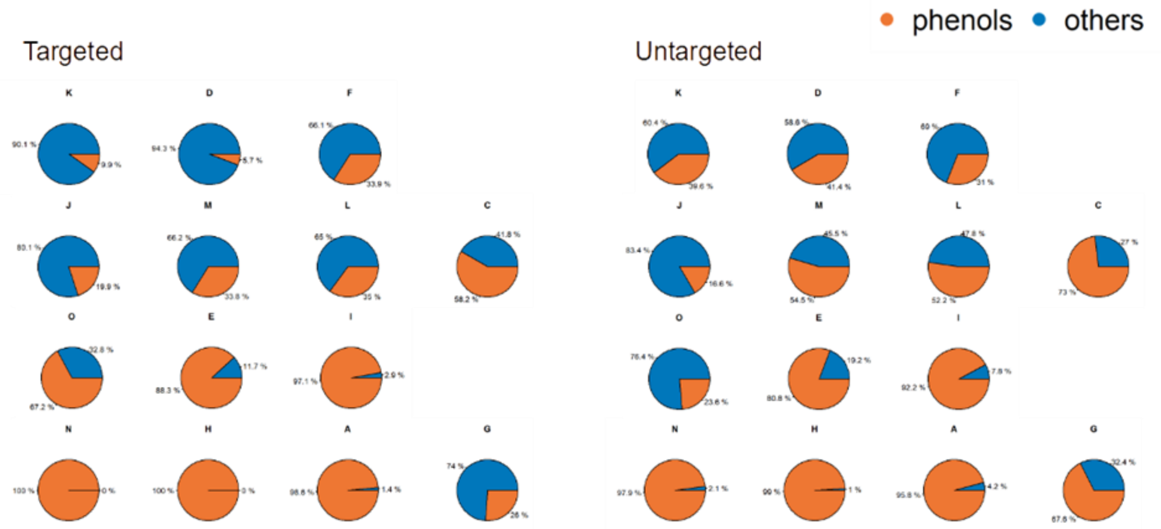

Table S3. Estimated daily intake (mg/day) of the quantified compounds according to the recommended dose.

Compound	K	D	F	J	M	L	C	O	E	I	N	H	A	G
Quinic acid	1-1.1	1.2-1.7	0.29-0.91	25.3-41.6	2.2-3	1.5-1.5	23-30.6	<LOQ	0.77-0.8	0.18-0.28	<LOQ	<LOQ	0.16-0.26	29.9-32
Malic acid	<LOQ	LOQ-0.29	<LOQ	13.4-16	1.5-1.6	0.96-1.1	5.7-7	<LOQ	1.1-1.6	0.16-0.16	<LOQ	<LOQ	0.08-0.15	7.9-9.2
Citric acid	<LOQ	ND - <LOQ	<LOQ	62.1-64.4	0.53-0.54	0.6-0.68	2.2-2.4	<LOQ	0.7-0.84	0.23-0.24	<LOQ	LOQ-0.001	0.16-0.18	7.7-8.4
Isocitric acid	ND	ND - <LOQ	ND - <LOQ	0.31-0.46	0.11-0.12	0.01-0.01	0.25-0.4	<LOQ	0.01-0.03	0.02-0.03	<LOQ	<LOQ	0.01-0.01	0.12-0.13
Succinic acid	<LOQ	<LOQ	<LOQ	<LOQ	0.15-0.17	<LOQ	0.42-0.57	<LOQ	<LOQ	0.22-0.25	<LOQ	<LOQ	<LOQ	0.2-0.22
Sorbitol	0.72-0.74	0.27-0.51	0.27-0.59	6.1-7.5	0.22-0.26	0.96-0.98	1.7-1.9	0.03-0.04	0.07-0.08	0.28-0.31	<LOQ	<LOQ	0.03-0.04	0.8-0.81
Trihydroxyoctadecenoic acid (I)	ND	ND - <LOQ	ND - <LOQ	ND	ND	ND	ND	ND	ND - <LOQ	ND	ND	ND	ND-0.01	0.01-0.02
Trihydroxyoctadecenoic acid (II)	ND - <LOQ	ND - <LOQ	ND - <LOQ	<LOQ	<LOQ	0.02-0.02	ND - <LOQ	ND - <LOQ	<LOQ	0.001-0.003	ND	ND	0.005-0.01	0.001-0.01
Trihydroxyoctadecenoic acid (III)	ND - <LOQ	ND - <LOQ	<LOQ	<LOQ	0.02-0.03	0.06-0.06	ND-0.03	0.003-0.01	0.06-0.07	0.07-0.09	ND	ND	0.01-0.01	0.01-0.02
Verbascoside	ND	ND-0.06	ND-0.05	1.4-2.6	0.28-0.32	0.18-0.19	5.6-6.1	ND-0.01	0.93-0.98	0.3-1.5	ND-0.03	ND-0.001	19-20.5	6.8-7.3
Hydroxy-verbascoside (I)	ND	ND	ND	ND	ND-0.04	ND	0.11-0.13	ND	0.18-0.19	0.03-0.07	ND	ND-0.001	1.2-1.2	0.3-0.35
Hydroxy-verbascoside (II)	ND	ND	ND	ND	ND-0.06	ND	0.77-0.86	ND	0.07-0.07	0.02-0.05	ND	ND	1.2-1.3	0.3-0.33
3,4-Dihydroxyphenylglycol	<LOQ	<LOQ	<LOQ	5.2-5.7	0.02-0.05	0.21-0.22	0.01-0.03	<LOQ	ND - <LOQ	0.04-0.05	<LOQ	0.003-0.004	0.12-0.15	0.04-0.04
Hydroxytyrosol	<LOQ	<LOQ	<LOQ	10.5-11.4	0.4-0.53	0.1-0.12	0.26-0.34	0.03-0.05	LOQ-0.07	6.9-8.6	7.7-11.3	1.9-2.2	6.0-8.0	3.7-4.1
Hydroxytyrosol glucoside (I)	ND - <LOQ	<LOQ	<LOQ	LOQ-1.5	<LOQ	<LOQ	1-1.3	<LOQ	0.14-0.2	0.23-0.26	LOQ-0.6	0.004-0.01	0.68-1.2	0.22-0.3
Hydroxytyrosol glucoside (II)	<LOQ	<LOQ	<LOQ	LOQ-1.4	0.15-0.2	0.39-0.42	4.4-4.8	<LOQ	0.22-0.31	0.31-0.37	LOQ-0.22	0.002-0.003	0.55-0.82	0.22-0.27
Tyrosol	0.19-0.2	0.09-0.1	0.1-0.12	ND	0.07-0.1	0.1-0.11	ND-0.42	0.03-0.03	ND-0.09	0.91-1.1	ND	0.05-0.06	2-2.3	1.3-1.3
Tyrosol glucoside	ND	ND - <LOQ	ND - <LOQ	ND	0.64-0.73	ND	3.5-4.7	ND	0.68-0.92	0.16-0.22	ND	ND	4.3-4.8	1.9-2.6
Oleuropein	<LOQ	LOQ-0.02	<LOQ	9.6-10.4	0.83-1.2	0.74-0.77	33-38.4	<LOQ	18.8-20.9	28.7-31.6	<LOQ	LOQ-0.002	1.6-1.9	0.07-0.09
Oleuropein isomer (I)	<LOQ	ND - <LOQ	<LOQ	ND - <LOQ	ND - <LOQ	ND	0.02-0.1	ND - <LOQ	ND-0.09	0.08-0.11	<LOQ	ND - <LOQ	0.005-0.01	LOQ-0.003
Oleuropein isomer (II)	<LOQ	<LOQ	<LOQ	LOQ-0.48	<LOQ	<LOQ	1.4-1.6	ND - <LOQ	0.25-0.31	1.2-1.5	<LOQ	<LOQ	0.04-0.05	0.02-0.03
Elenolic acid	<LOQ	<LOQ	ND	0.49-0.61	<LOQ	0.06-0.08	<LOQ	<LOQ	<LOQ	0.04-0.05	ND - <LOQ	ND	0.48-0.55	0.17-0.18
DEDA	<LOQ	<LOQ	LOQ-1.4	<LOQ	<LOQ	<LOQ	<LOQ	LOQ-0.004	<LOQ	0.09-0.1	LOQ-0.22	0.07-0.08	0.29-0.33	0.74-0.85
Hydroxy-DEDA	<LOQ	<LOQ	<LOQ	<LOQ	<LOQ	0.38-0.42	<LOQ	<LOQ	<LOQ	<LOQ	LOQ-0.05	0.002-0.002	0.06-0.06	0.64-0.71
DEDA hydrated (I)	<LOQ	<LOQ	<LOQ	<LOQ	<LOQ	<LOQ	<LOQ	<LOQ	<LOQ	0.04-0.05	LOQ-0.15	0.02-0.03	0.03-0.04	0.02-0.04
DEDA hydrated (II)	ND - <LOQ	ND - <LOQ	ND - <LOQ	<LOQ	<LOQ	<LOQ	<LOQ	<LOQ	<LOQ	<LOQ	<LOQ	0.003-0.003	<LOQ	<LOQ

Table S4. Parameters used for data processing with XCMS

Step	Parameter	Value
Peak detection	Method	centWave
	Relative deviation in the m/z dimension (ppm)	20
	Peak width (range in seconds)	2, 40
	Prefilter (scans, intensity)	5, 1,000,000
	Signal-to-noise threshold	100
	Noise	3000
	Binning width in the profile matrix	0.001
	Integration method	2
Peak filtering	Number of data points	5
	Minimal intensity	1,000,000
Peak post-processing	expandRt (seconds)	2
	expandMz (Da)	0.001
	ppm	10
	Intensity proportion	0.66
Aligment	Method	Loess
	Subset adjustment: method, samples	Average, QC
	Degree of smoothing for local polynomial regression fitting	0.3
	Fraction of samples a peak should be found in	1
Peak grouping	Sample group definition	Commercial product
	Fraction of samples a peak should be found in	1
	m/z width	0.02
	Bandwidth of peaks	3/2
Peak filling	Method	ChromPeakAreaParam

Table S5. Analytical standards used for the targeted analyses.

Compound	Molecular Formula	PubChem CID	Chemical structure
Quinic acid	C7H12O6	6508	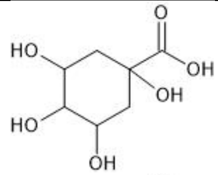
Malic acid	C4H6O5	525	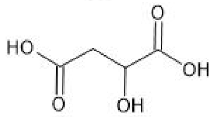
Isocitric acid	C6H8O7	1198	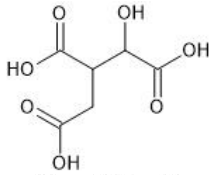
Citric acid	C6H8O7	311	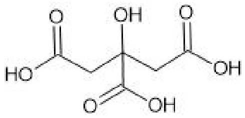
Succinic acid	C4H6O4	1110	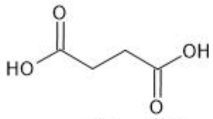
Sorbitol	C6H14O6	5780	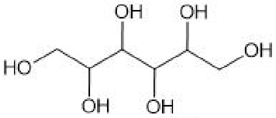
3,4-Dihydroxyphenylglycol	C8H10O4	91528	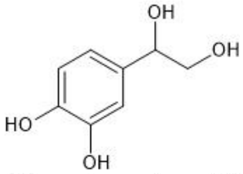
Hydroxytyrosol	C8H10O3	82755	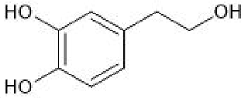
Oleuropein	C25H32O13	5281544	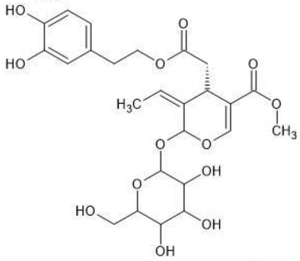
Tyrosol	C8H10O2	10393	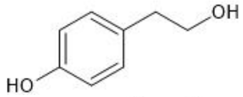
Tyrosol glucoside (salidroside)	C14H20O7	159278	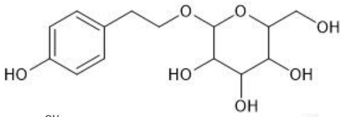
Verbascoside	C29H36O15	5281800	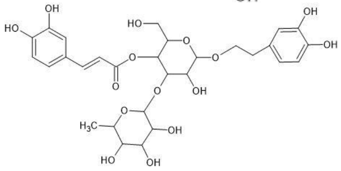
